# Genetic mapping of distal femoral, stifle, and tibial radiographic morphology in dogs with cranial cruciate ligament disease

**DOI:** 10.1371/journal.pone.0223094

**Published:** 2019-10-17

**Authors:** Eleni Healey, Rachel J. Murphy, Jessica J. Hayward, Marta Castelhano, Adam R. Boyko, Kei Hayashi, Ursula Krotscheck, Rory J. Todhunter

**Affiliations:** 1 College of Veterinary Medicine, Cornell University, Ithaca, NY, United States of America; 2 Department of Biomedical Sciences and Cornell Veterinary Biobank, College of Veterinary Medicine, Cornell University, Ithaca, NY, United States of America; 3 Department of Clinical Sciences and Cornell Veterinary Biobank, College of Veterinary Medicine, Cornell University, Ithaca, NY, United States of America; Huazhong Agriculture University, CHINA

## Abstract

Cranial cruciate ligament disease (CCLD) is a complex trait. Ten measurements were made on orthogonal distal pelvic limb radiographs of 161 pure and mixed breed dogs with, and 55 without, cranial cruciate partial or complete ligament rupture. Dogs with CCLD had significantly smaller infrapatellar fat pad width, higher average tibial plateau angle, and were heavier than control dogs. The first PC weightings captured the overall size of the dog’s stifle and PC2 weightings reflected an increasing tibial plateau angle coupled with a smaller fat pad width. Of these dogs, 175 were genotyped, and 144,509 polymorphisms were used in a genome-wide association study with both a mixed linear and a multi-locus model. For both models, significant (p_genome_ <3.46×10^−7^ for the mixed and< 6.9x10^-8^ for the multilocus model) associations were found for PC1, tibial diaphyseal length and width, fat pad base length, and femoral and tibial condyle width at *LCORL*, a known body size-regulating locus. Other body size loci with significant associations were growth hormone 1 (*GH1*), which was associated with the length of the fat pad base and the width of the tibial diaphysis, and a region on CFAX near *IRS4* and *ACSL4* in the multilocus model. The tibial plateau angle was associated significantly with a locus on CFA10 in the linear mixed model with nearest candidate genes *BET1* and *MYH9* and on CFA08 near candidate genes *WDHD1* and *GCH1*. *MYH9* has a major role in osteoclastogenesis. Our study indicated that tibial plateau slope is associated with CCLD and a compressed infrapatellar fat pad, a surrogate for stifle osteoarthritis. Because of the association between tibial plateau slope and CCLD, and pending independent validation, these candidate genes for tibial plateau slope may be tested in breeds susceptible to CCLD before they develop disease or are bred.

## Introduction

Cranial cruciate ligament disease (CCLD), one of the most common orthopedic disorders of dogs, results in partial to complete rupture of the cranial cruciate ligament (CCL), which in turn, results in stifle destabilization, osteoarthritis, and hind limb lameness. Breeds at increased risk include the Newfoundland, rottweiler, Labrador retriever, bulldog, boxer, chow chow, American Staffordshire terrier, St. Bernard, West Highland white terrier, golden retriever, and Yorkshire terrier.[1, 2] In contrast, breeds with the least risk of developing CCLD include the miniature dachshund, dachshund, greyhound, Shih Tzu, miniature schnauzer, and Pekingese.[1] The cocker spaniel was reported in one study to have a reduced risk.[2] Within breeds, increased body weight has been associated with a higher risk of CCLD.[2] Several studies found that the incidence of CCLD increased with age, with the median age at diagnosis of 7–10 years.[3–5]

While studies have shown that spaying and neutering dogs can improve their health and increase their lifespan, these practices have been associated with an increased risk of developing CCLD in both male and female dogs.[1, 6] Some studies have reported a greater association between female dogs and CCLD than male dogs.[5, 7] However, others have noted no difference in disease incidence between the sexes.[6]

In addition to breed, body weight, age, and sex predispositions, there are differing conclusions regarding the role of the tibial plateau angle (TPA), or caudal tibial slope, in the pathogenesis of CCLD. Measurement of this angle is necessary for surgeries performed to correct CCLD in dogs, such as a tibial plateau leveling osteotomy (TPLO), which decreases TPA, and tibial tuberosity advancement, which is designed to neutralize this slope.

Several studies have found an association between increased TPA and CCLD, encouraging the use of TPA as a measurement for CCL strain and to predict CCLD.[8, 9] Other studies have found no association between increased TPA and CCLD, and, as a result, have cautioned against the use of TPA to determine the risk of CCLD.[10] Furthermore, while one study did not conclude TPA to be significantly different between dogs with CCLD and dogs without CCLD, the findings suggested that increased TPA may be associated with increased severity of stifle radiographic osteoarthritis (OA) in dogs with CCLD.[11] In addition, another study found a separate yet related measurement, the anatomical-mechanical axis angle, to more accurately predict CCLD than TPA.[12]

Cranial cruciate ligament disease is a complex trait controlled by genetic factors with environmental regulation. In linkage analysis of 271 Newfoundland dogs, quantitative trait loci (QTL) for CCLD were found on canine chromosome (CFA) 03, 05, 13, and 24[13] and a genome wide association study (GWAS) pointed to loci on CFA 01, 03 and 33 with CCLD in the same pedigree.[14] Single nucleotide polymorphisms (SNPs) were located within genes involved in neurological regulation suggesting the potential effect of neural dysfunction on CCLD onset and progression.[14] Another study employed candidate gene analysis in Newfoundlands, Labrador retrievers, Staffordshire bull terriers, and rottweilers to genotype 196 SNPs across 28 candidate genes, which were selected based on their potential contribution to the structure of the CCL or CCLD progression.[15] No polymorphisms in candidate genes encoding collagen and other components of the extracellular matrix were discovered.[15]

Most recently, a GWAS for CCLD, including 237 Labrador retrievers, reported one SNP within a 5kb haplotype block on CFA24 which met genome-wide significance.[16] Within this block, 9 genes influenced tissue homeostasis and, thus, might impact CCL function.[16] Our most recent GWAS of 670 dogs of different breeds found three SNPs associated with CCLD; one each on chromosomes 7, 8, and 9, with *CLMN* and *DYN* as positional candidate genes.[17] Most recently, Baker[18] reported a multivariate GWAS based model of CCLD, TPA, and tibial tuberosity width in which they identified 3 loci with moderate evidence of association that were not previously associated with CCLD. A locus on CFA01 was associated with both CCLD and tibial tuberosity width located within *ROR2*, a gene implicated in cartilage and bone development. A polymorphism on CFA04 was associated with both CCLD and TPA and was within *DOCK2*, a gene shown to promote immune cell migration and invasion in synovitis, an important predictor of CCLD. A third locus on CFA23 was associated with only CCLD and was near a long non-coding RNA (lncRNA).

Principal component analysis (PCA) reduces many measurements into their correlated components with each PC independent of the others and these PCs can be analyzed like a traditional phenotype. We have used PCA to analyze the genetic basis of other canine orthopedic traits including pelvic morphology.[19] The present study used PCs of 10 pelvic limb radiographic measurements, as well as the individual measurements (some of which are correlated to CCLD and secondary OA), in a linear mixed and a multi-locus model GWAS. We report significant associations of the TPA with loci on chromosomes 8 and 10. In addition, a locus on chromosome 3 (*LCORL*), previously associated with body size in several species, was significantly associated with PC1 and individual pelvic limb measurements. Mutations in candidate genes in these associated genomic regions may provide further insight into the genetic predisposition to CCLD and its environmental regulation. Veterinarians, owners and dog breeders need better tools to inform preventative strategies and breeding decisions, and better therapy to prevent CCLD and the secondary OA that encumbers affected dogs.

## Materials and methods

### Dogs

We measured a subset of stifle radiographs which were used to diagnose stifle OA resulting from CCLD in a previous GWAS[17, 20] and radiographs on additional dogs were added for the PCA. Dogs had stifle radiography at the Cornell University Hospital for Animals and a subset of these dogs had been genotyped. The full set included 161 control dogs and 55 dogs with CCLD. Dogs included in this study represented 38 pure breeds and mixed-breed dogs.

### Radiographic measurements

Some dogs had lateral stifle radiographs taken with the long axes of the femur and stifle at 90° (as is customary for preparation for TPLO surgery), others had lateral radiographs taken with the femur and tibia at ~135° (as is customary for tibial tuberosity advancement surgery), while some radiographs were taken with the femur and tibia at angles in between 90°and 135°.

Both lateral and cranial-caudal radiographic projections of one or both distal pelvic limbs including the stifle, the tarsus and the distal femur were required for inclusion of the dog in this study. If any abnormalities altering bony dimensions or alignment of the femur or tibia were noted, including extensive stifle bony proliferation/osteolysis or healed and malaligned fractures, the dog was excluded. Dogs less than three years of age were excluded due to their low number. If multiple studies were available, the most recent study was selected for analysis, unless the dog was better positioned on earlier radiographs. Radiograph positioning criteria for lateral projections were based on the superposition of the femoral condyles. Positioning criteria for cranial-caudal projections were evaluated on condyle and proximal tibial symmetry and patellar location in the center of the trochlea groove. The best positioned single limb radiograph was used from each dog.

**Fig 1:**
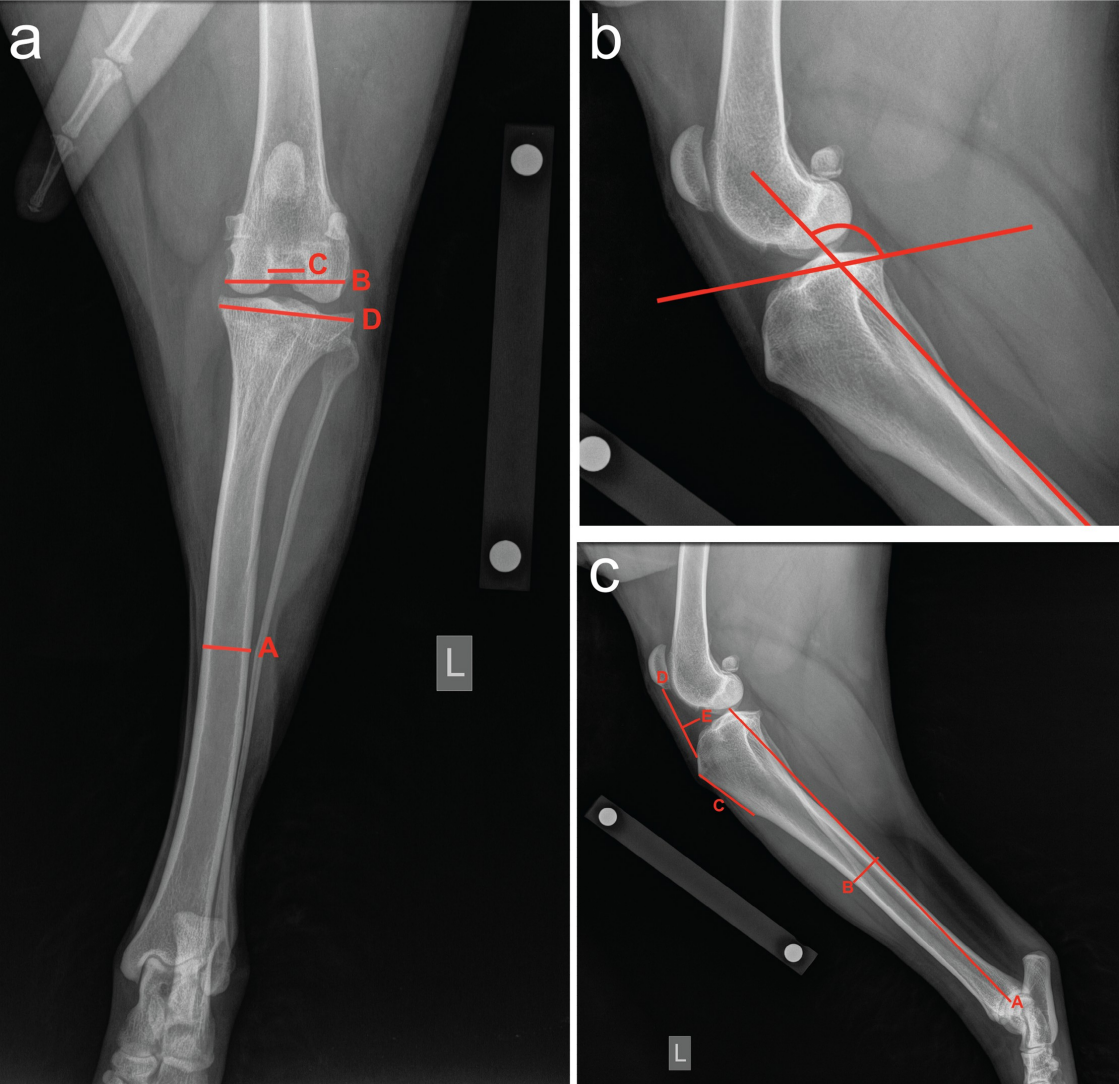
Illustration of radiographic measurements of dog stifles and tibiae. Panel A. Cranial-caudal radiograph shows the mid-diaphyseal tibial width (A), the femoral condyle width (B), the femoral notch width (C), and the proximal tibial width (D). Panel B. Lateral radiograph of proximal tibia illustrating the tibial plateau angle (see Table 1 for description). Panel C. Lateral tibial radiograph illustrating the tibial length (A), the tibial diaphyseal width (B), the tibial tuberosity length (C), the infrapatellar fat pad height (D), and the infrapatellar fat pad width (E).

Radiographs were measured in PACS imaging software (https://www.carestream.com/en/us/pacs-software). Each radiograph was scaled to the original size of the patient using a 100 mm internal calibration tool placed at bone level when the image was taken. Ten measurements were generated for each patient; six measurements on the lateral projection and four on the cranial-caudal projection (Table 1; Fig 1).

**Table 1:** Radiographic measurements of the stifle and tibia used to derive principal components and phenotypes for genome wide association study.

Lateral projection	Radiographic measurement
Tibial plateau angle	One line connecting the center of the talus to the center of the tibial intercondylar eminences. A second line along the tibial condyles connecting the point just caudal the insertion of the cranial cruciate ligament and extending caudally to pass through the caudal tibial plateau as it deviated distally to join the proximal tibial metaphysis. The obtuse angle between these two intersecting lines was the TPA.
Tibia length	A line originating at the center-point of the lateral malleolus of the fibula and ending at the proximal intercondylar eminence.
Tibial diaphyseal width	The distance between the outside cortices at the mid tibial diaphysis
Tibial tuberosity length	A line connecting the proximal and distal tibial tuberosity
Infrapatellar fat pad height	The distance at the caudal edge of the patellar ligament from the distal patella to its insertion on the tibial tuberosity
Infrapatellar fat pad width	At its widest point, a line perpendicular to the height of the infrapatellar fat pad caudal to the edge of the soft tissue opacity within the joint space
**Cranial Caudal Projection**	
Tibial diaphyseal width	At the mid-point of the tibial diaphysis, the width to each outer cortex
Femoral condyle width	A line perpendicular to the long axis of the femur at the level of the base of the intercondylar fossa, extending from the medial to the lateral femoral condyles
Femoral notch width	A line perpendicular to the long axis of the femur at the mid-point of the intercondylar fossa and extending between the axial borders of the femoral condyles
Tibial plateau width	A line perpendicular to the long axis of the tibia at the proximal aspect of the tibial metaphysis

The same person (EH) made all the measurements after instruction from an experienced surgeon (RJT). We tested the TPAs for internal consistency by randomly sampling 20 individual radiographs and remeasuring the TPA, resulting in a correlation of 0.86.

A Shapiro-Wilk test was used to assess if the measurements were normally distributed. All measurements, except the TPA, were not normally distributed (p<0.05). All the measurements, except the TPA, were significantly different in males compared to females based on an unpaired 2-tailed t-test. To adjust each measurement according to the dog’s sex, each measurement was modeled as a linear function of sex. The Box-Cox command in the R package "MASS" was used to transform the measurements (Figure A in S1 File). Unpaired 2-tailed t tests were used to compare body weights, age, TPA, and transformed fat pad height and width between the dogs with and without CCLD. P<0.05 was considered significant.

### Principal component analysis (PCA)

Principal component analysis was performed on 10 sex-adjusted and transformed radiographic measurements of 216 stifles using the prcomp function in R [21] Body weights, expressed as body weight^0.303^ based on a Box-Cox transformation to normalize the distribution of body weight across breeds, were available for 151 dogs with CCLD and 55 control dogs. We regressed PC1 against body weight to determine if there was a significant relationship between them with P<0.05 considered significant.

### Genome-wide association study (GWAS)

DNA was stored at the Cornell Veterinary Biobank (https://www.vet.cornell.edu/departments/centers/cornell-veterinary-biobank). Genotypes were used from previous reports.[17, 20] In brief, genotyping was performed on a semi-custom Illumina 173k CanineHD mapping array, supplemented with 12,143 markers for a total of 185,805 markers [see PLINK genotype files by Hayward[20] that are deposited in Dryad (datadryad.org, doi:10.5061/dryad.266k4)]. PLINKv1.07[22] was used to remove SNPs with a genotyping rate below 95%, discordant SNPs between duplicate samples, SNPs that deviated from Hardy-Weinberg equilibrium, and any mitochondrial or Y chromosome SNPs with heterozygous calls [20]. After these filtering steps, 180,117 SNPs remained for analyses. For GWAS, the PCs and the transformed measurements were analyzed in a linear mixed model using the program GEMMA v.0.94[23] which included a relatedness matrix (estimated using centered genotypes) and the Wald Test was used to determine P-values. In the model Y = Wα + Xß + u + ε, where W is a n × (c+1) matrix of covariates, α is the (c+1)× 1 vector of covariate effects including intercept, X is the genotype data, ß is the effect size, u is a random effect (including the n×n relatedness matrix), ε is a random error term, the fixed effects are W, α, ß and the random effects (u and ε), are assumed to have a normal distribution. We excluded SNPs with a minor allele frequency (MAF) <5%, resulting in 144,509 SNPs remaining for the GWAS, and a significance threshold of P<3.46×10^−7^ (the Bonferroni-adjusted genome wide P-value<0.05) was used.

In addition to the linear mixed model, we implemented a multi-locus model using the R package FarmCPU[24], which reduces false negatives which can be observed in linear mixed model GWAS. We used the default parameters (MAF threshold of 5%, maximum of 10 loops or iterations, Bonferroni-corrected threshold calculated with alpha = 0.01 producing p_genome_ <6.9x10^-8^) and included a population structure matrix as a covariate file. This covariate file consisted of the first 10 PCs from a PCA of the genotypes on all 175 dogs. Manhattan and Quantile-Quantile (QQ) plots were created in R[21]. QQ plots were used to show the distributions of our observed GWAS *P*-values compared to the expected *P*-values. Significant associations of markers and CCLD deviate from a uniform distribution, that is, they do not follow the diagonal X = Y line on a QQ plot.

## Results

### Dogs

The 161 dogs with CCLD and 55 dogs without CCLD included 77 Labrador retrievers, 20 golden retrievers, 18 mixed breed dogs, 17 German shepherd dogs, 16 rottweilers, and 62 other pure breed dogs with less than 10 dogs per breed. Of these, 175 dogs were genotyped. Descriptive statistics for the 10 radiographic measurements (Table A in S1 File) are shown in Table 2.

**Table 2:** Descriptive statistics summary of the 10 radiographic measurements described in Table 1. All measurements except the tibial plateau angle are in mm. The number in parentheses after the column heading is the number used for the Box-cox transformation as described in the Materials and Methods Section.

	Tibial plateau angle	Tibia length (1.9)	Lateral diaphysis (2.0)	Tuberosity length (1.0)	Fatpad base (0.7)	Fatpad height (0.1)	Cranial diaphysis (1.9)	Femoral condyle (1.7)	Femoral notch (1.8)	Tibia condyle (2.0)
Min.	01.0	32.6	0.2	0.9	0.9	0.9	0.3	1.1	0.2	2.6
LQ	13.7	281.7	2.3	2.1	2.3	0.1	2.2	7.8	1.0	15.8
Median	116.2	329.7	2.6	3.3	2.5	0.1	2.8	9.3	1.2	19.3
Mean	116.1	329.2	2.7	3.3	2.4	0.1	2.7	9.3	1.2	19.2
UQ	118. 9	387.4	3.1	3.7	2.7	1.0	3.1	10.5	1.4	23.1
Max.	126.2	829.3	5.4	7.4	3.5	1.1	6.1	17.8	2.6	39.3

Body weight for CCLD dogs (39.1±SD = 11.0 kg) was significantly higher than control dogs (28.1±15.2 kg) (unpaired t-test, t = 5.508, df = 202, P<0.0001). Dogs with CCLD were significantly younger (65±32 months) than control dogs (84±51 months) (unpaired t-test, t = 3.392, df = 201, P = 0.0008). Even though the difference was not large, the TPA was significantly higher in dogs with CCLD (116.6±3.9°) than in control (114.5±4.8°) dogs (unpaired t-test, t = 3.261, df = 214, P = 0.0013). Fat pad width was significantly larger (2.5±0.3 mm) in CCLD dogs than in control (2.3±0.6 mm) dogs (unpaired t-test, t = 3.163, df = 214, P = 0.0018) and fat pad height was significantly smaller (0.98±0.02 mm) in CCLD dogs than in control (1.0±0.03 mm) dogs (unpaired t-test, t = 6.795, df = 214, P = 0.0001).

### Principal component analysis

Body weight and PC1 were significantly correlated (r = 0.79, t = 15.896, df = 164, P<0.001)). The first four PCs explained almost 94% of the total variance (Table 3).

**Table 3:** Eigenvalue, variance, and cumulative variance of the 10 principle components.

	Eigenvalue	Variance	Cumulative Variance
PC1	7.3	73.0	73.0
PC2	1.1	10.8	83.8
PC3	0.8	6.8	90.6
PC4	0.3	3.1	93.7
PC5	0.2	2.1	95.8
PC6	0.1	1.1	97.3
PC7	0.1	0.1	98.2
PC8	0.1	0.9	99.1
PC9	0.1	0.6	99.8
PC10	0.0	0.2	100.0

**Fig 2:**
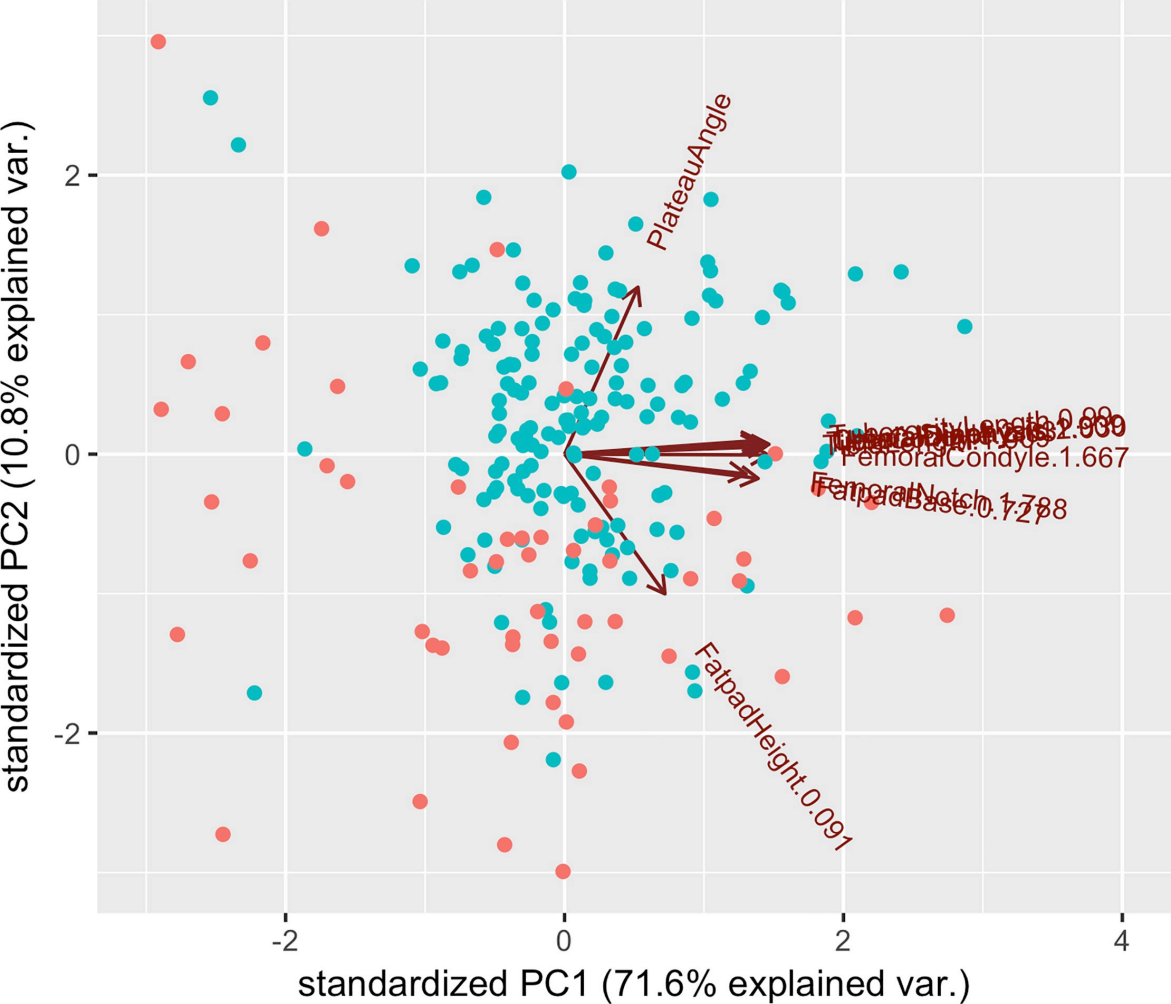
Plot of PC1 and PC2 values according to whether a dog had CCLD (red) or was a control (blue).

**Fig 3:**
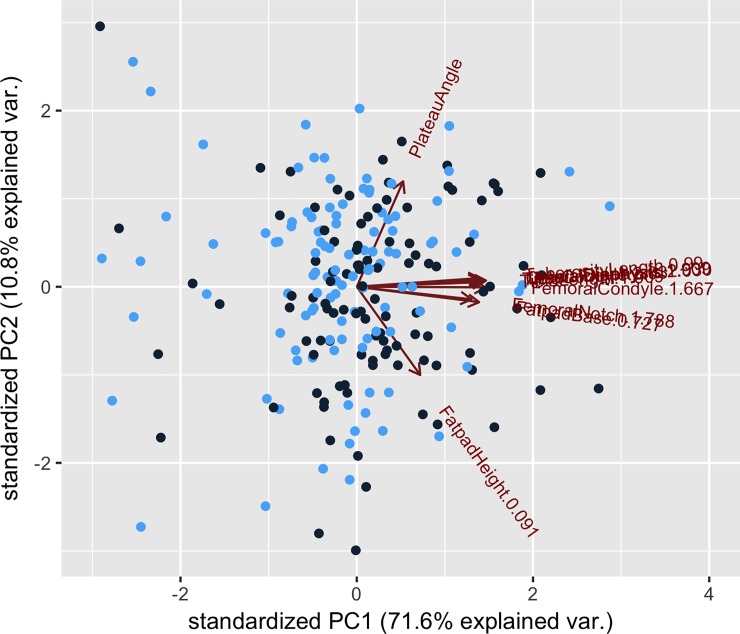
Plot of PC1 against PC2 values for male (black) and female (blue) dogs.

A plot of PC1 against PC2 showed that the dogs with CCLD tended to cluster closer together more than the control group (Fig 2) but not according to sex of the dogs (Fig 3). Each measurement was weighted approximately equally for PC1 except for the TPA and the fat pad height (Table 4).

**Table 4:** Composition of the 10 principal components (PCs) with the weighting of each individual pelvic limb measurement.

Measurement Location	PC 1	PC 2	PC 3	PC 4	PC 5	PC 6	PC 7	PC 8	PC 9	PC 10
Plateau angle	0.13	0.75	-0.63	-0.04	0.07	-0.05	0.03	-0.01	0.00	-0.00
Tibial length 1.91	0.35	0.03	0.02	0.22	-0.40	0.16	-0.19	-0.12	-0.76	-0.07
Lateral diaphysis 2.03	0.35	0.05	0.11	0.12	0.32	0.04	-0.83	-0.03	0.25	0.02
Tuberosity length 0.99	0.34	0.06	0.04	0.41	-0.44	0.35	0.19	0.37	0.48	-0.01
Fat pad base 0.73	0.34	-0.11	-0.01	0.27	-0.14	-0.84	0.12	-0.19	0.13	-0.04
Fat pad height 0.09	0.18	-0.63	-0.71	0.07	0.16	0.16	0.02	-0.00	-0.01	0.06
Cranial diaphysis 1.91	0.34	0.05	0.20	0.12	0.61	-0.05	0.32	0.51	-0.29	0.01
Femoral condyle 1.67	0.36	-0.00	0.11	-0.24	0.15	0.23	0.24	-0.44	0.14	-0.68
Femoral notch 1.79	0.32	-0.09	-0.03	-0.77	-0.29	-0.17	-0.12	0.41	0.00	0.00
Tibia condyle 2.03	0.36	0.04	0.15	-0.19	0.06	0.17	0.24	-0.44	0.14	0.68

The main contributions to PC2 came from the TPA and the fat pad height but in the opposite direction. That is, as the TPA increased the fat pad height decreased as expected because as the TPA increased the dogs were more likely to have CCLD, and hence fat pad compression from synovitis and OA. However, not every dog followed this association because the main weightings on PC3 were from the same two measurements but in this case, lower angles and smaller fat pad height were seen. For PC6, longer tibial tuberosities and wider femoral and tibial condyles were weighted positively while the fat pad base was negatively weighted and it provided the major contribution to PC6.

### Genome wide association study

**Fig 4:**
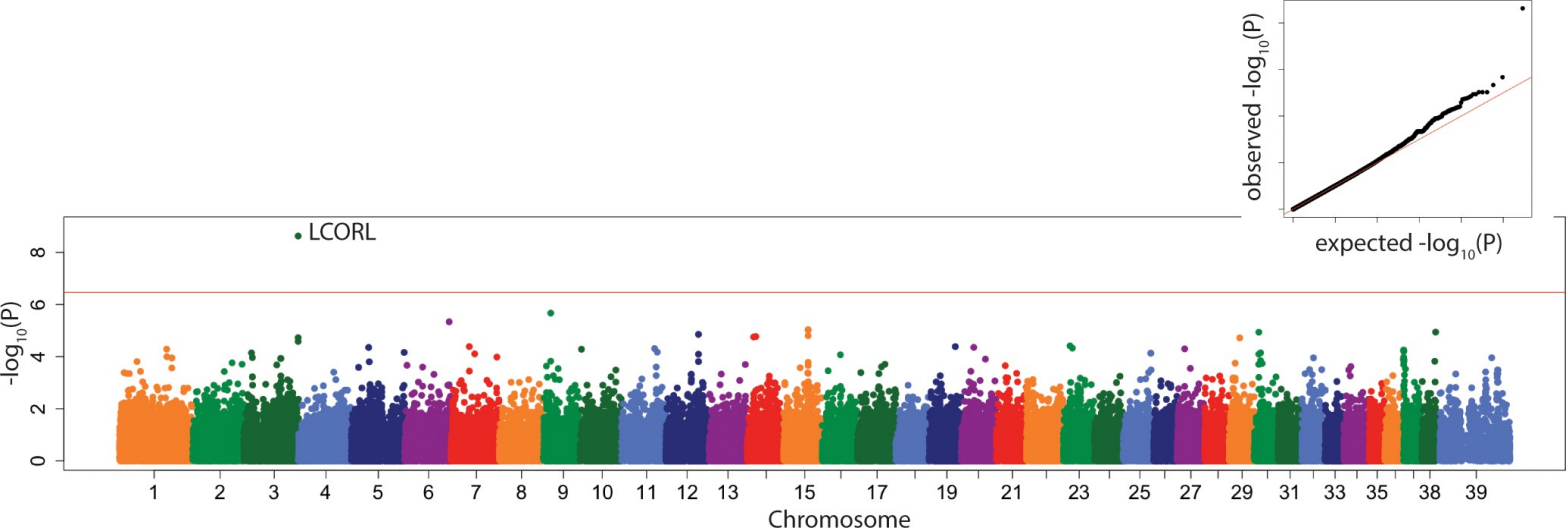
Manhattan plot of linear mixed model GWAS of PC1. Marker position plotted on the X axis against –log_10_(P) on the Y axis. The Bonferroni adjusted genome wide p value threshold is drawn as the red line across the plot. QQ plot of expected –log_10_(P) for no association against observed –log_10_(P) is shown as insert.

In the 175-dog subset with both genotypes and radiographs, a well-recognized locus CFA03:91114590 which contains *LCORL*, a gene which contributes strongly to body size of many species, including the dog,[4, 20, 25, 26] was significantly associated with PC1 (Fig 4), in both the linear mixed model and the multi-locus model.

This locus was also associated significantly with tibial length (Figure B in S1 File), lateral tibial diaphyseal width (Figure C in S1 File), tibial tuberosity length (Figure D in S1 File), fat pad base (Figure E in S1 File), and femoral (Figure F in S1 File) and tibial (Figure G in S1 File) condyle width in both the linear mixed model and the multi-locus model (Tables 5 and 6).

**Table 5:** Genome wide significant marker associations of 10 radiographic morphologic measurements and their principal components, chromosome position, effect size, p value and candidate gene(s) in the associated interval following a linear mixed model GWAS performed in GEMMA.

Phenotype	Chr	Position (bp)	Effect size	*P*-value	Candidate Genes
PC1	3	91114590	1.64	2.37E-09	*LCORL*
PC6	35 23	1776352 11346684	0.31 0.29	8.26E-08 1.41E-07	*FOXQ1* *TRAK1*
Tibial Plateau Angle	10 8	28002796 30830592	2.01 2.40	2.25E-07 3.33E-07	*BET1*, *MYH9* *WDHD1*
Tibial Length	3	91114590	68.29	2.53E-08	*LCORL*
Diaphyseal Width Lateral Projection	3	91114590	0.59	6.13E-08	*LCORL*
Tuberosity Length	23	11389112	0.65	2.16E-07	*TRAK1*
Fat Pad Base	9 3	12074972 91114590	-0.28 0.22	4.47E-08 1.60E-07	*GH1* *LCORL*
Tibial Diaphyseal Width Cranial Caudal Projection	3 9	91114590 12074972	0.56 -0.61	2.75E-09 3.01E-07	*LCORL* *GH1*
Femoral Condyle Width Cranial Caudal Projection	3	91114590	1.65	7.58E-09	*LCORL*
Tibia Condyle Width Cranial Caudal Projection	3	91114590	3.88	4.53E-08	*LCORL*

**Table 6:** Genome wide significant marker associations of 10 radiographic morphologic measurements and their principal components, chromosome position, effect size, p value and candidate genes in the associated interval following a multi-locus model GWAS performed using FarmCPU. Only significant locus associations have candidate genes listed. FarmCPU uses a 1% threshold for significance i.e. 0.01/number of markers used in GWAS = 6.9x10^-8^. # indicates same locus as for the mixed linear model in GEMMA.

Phenotype	Chr	Position (bp)	Effect size	*P*-value	Candidate Genes
PC1	3 X 11 9 6 20	91114590 29270503 59866929 43240534 74671230 12792269	-0.96 0.67 -0.94 0.77 -0.87 0.78	3.11E-11# 5.93E-10 7.65E-10 1.07E-09 7.88E-09 2.61E-08	*LCORL* *NPC2* *-* *MYO18A* *NEGR1* *ITPR1*
PC4	16 7 13 27	30675811 61531148 50399261 37583571	-0.29 0.36 -0.20 -0.13	1.45E-14 2.63E-14 2.46E-10 2.57E-08	*-* *CHST9* *-* *VMN2R*
PC6	19 2 29 24	43088987 34373078 38879394 16282763	0.17 -0.26 -0.25 -0.10	2.54E-14 1.89E-11 1.07E-10 4.37E-08	*SOCS2* *DIP2C* *PDP1* *GPCPD1*
Tibial Length	3 5 11 24 6	91114590 29898790 59196021 43761051 26793945	-51.06 -58.56 48.73 40.55 21.95	1.38E-17# 4.04E-14 5.14E-10 1.69E-09 3.65E-08	*LCORL* - *CYCL2* *TUBB1* *XYLT1*
Tibial Diaphyseal Width Lateral Projection	14 3 11 30	23479385 91114590 36466907 13503286	0.29 -0.33 0.23 0.24	5.14E-12 1.89E-10# 8.90E-10 7.09E-09	*UMAD1* *LCORL* *BNC2* *SEMA6D*
Tuberosity Length	5 23 3 33	29898790 11389112 91114590 29970487	-0.39 -0.43 -0.29 0.27	2.32E-11 4.15E-10# 1.59E-08 2.28E-08	*-* *TRAK1* *LCORL* *MELTF*, *DLG1*
Fat Pad Base	3 1 1 37	91114590 117449774 79362949 672739	-0.15 -0.10 0.19 -0.20	1.23E-12# 3.45E-10 1.47E-08 2.23E-08	*LCORL* *FXYD1*, *LGI4*, *HPN* *TLE4* *PMS1*
Fat Pad Height	16 37	30712079 25059163	-0.01 0.01	1.36E-09 4.93E-08	*-* *SLC11A*, *CATIP*, *CTDSP1*

Another previously observed canine body size locus[27–29], at CFA39:82,673,593 bp near the genes *IRS4* and *ACSL4*, was significantly associated with femoral and tibia condyle width in the multi-locus model only. A locus at CFA23:11,346,684 with the closest candidate genes, *TRAK1* and *CCK*, was associated significantly with tibial tuberosity length in both models, and also with PC6 in the linear mixed model.

**Fig 5:**
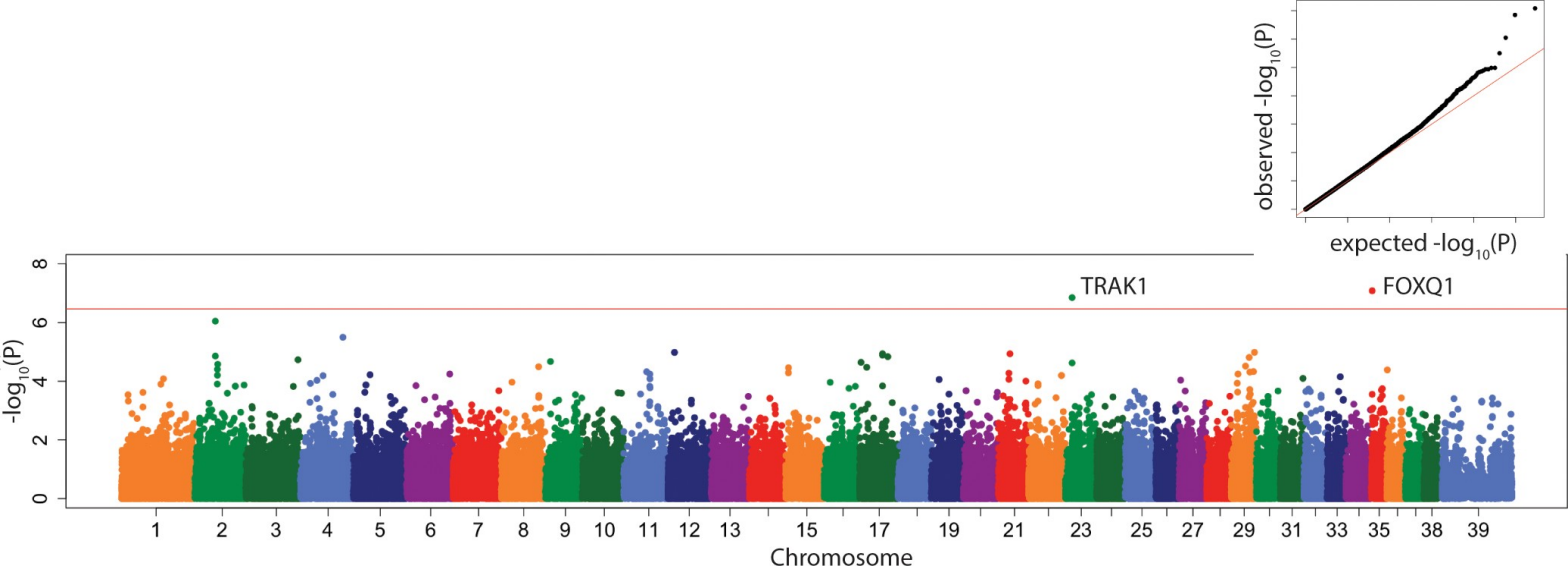
Manhattan plot of linear mixed model GWAS of PC6. Marker position plotted on the X axis against –log_10_(P) on the Y axis. The Bonferroni adjusted genome wide p value threshold is drawn as the red line across the plot. QQ plot of expected –log_10_(P) for no association against observed –log_10_(P) is shown as insert.

In general, we saw many more significant associations using the multi-locus model than the linear mixed model (Figures H-R in S1 File), but there were several significant associations that were only seen in the linear mixed model, as follows. PC6 was significantly associated with locus CFA35:1,776,352 with the closest candidate gene *FOXQ1* (Fig 5).

**Fig 6:**
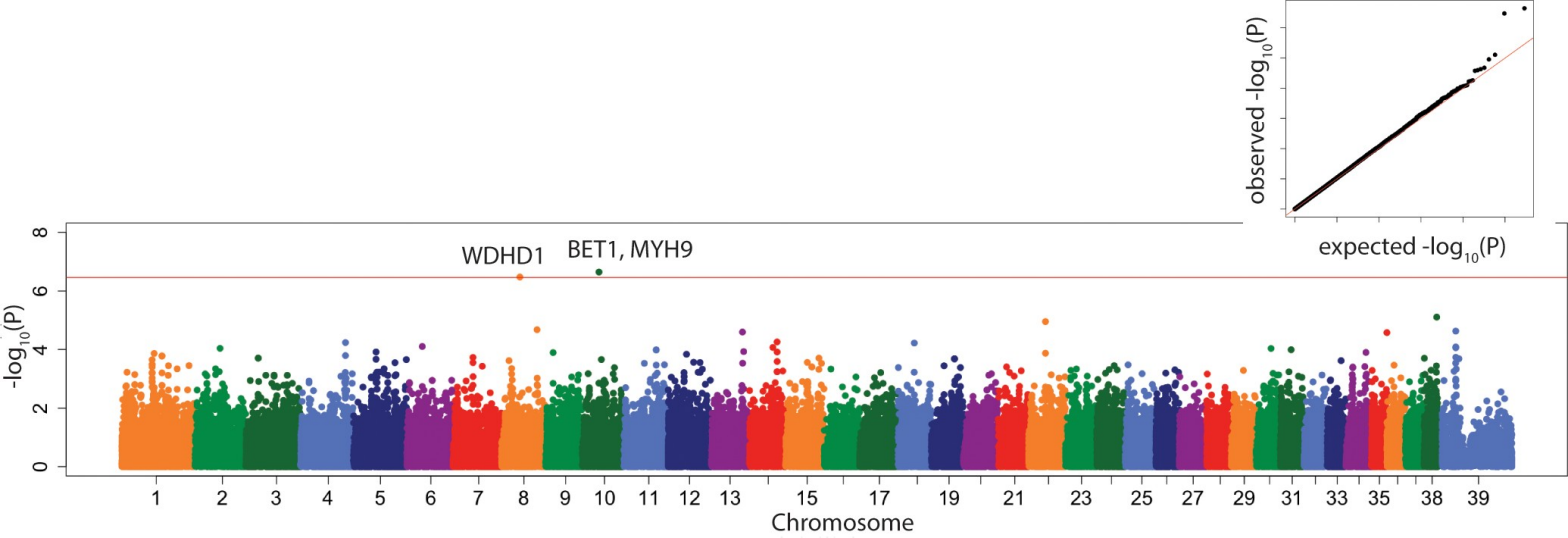
Manhattan plot of linear mixed model GWAS of tibial plateau angle. Marker position plotted on the X axis against –log_10_(P) on the Y axis. The Bonferroni adjusted genome wide p value threshold is drawn as the red line across the plot. QQ plot of expected –log_10_(P) for no association against observed –log_10_(P) is shown as insert.

Tibial plateau angle was significantly associated with a locus on CFA10:28,002,796 with nearest candidate genes *BET1* and *TXN2* about 50kb upstream and *MYH9* (Fig 6) about 80 kb downstream.

The locus CFA08:30,830,592 near candidate gene *WDHD1*, was also associated significantly with TPA. Growth hormone 1 (*GH1*) on CFA09:12074972, which regulates body mass, was associated significantly with the length of the fat pad base and the size of the tibial diaphysis.

**Fig 7:**
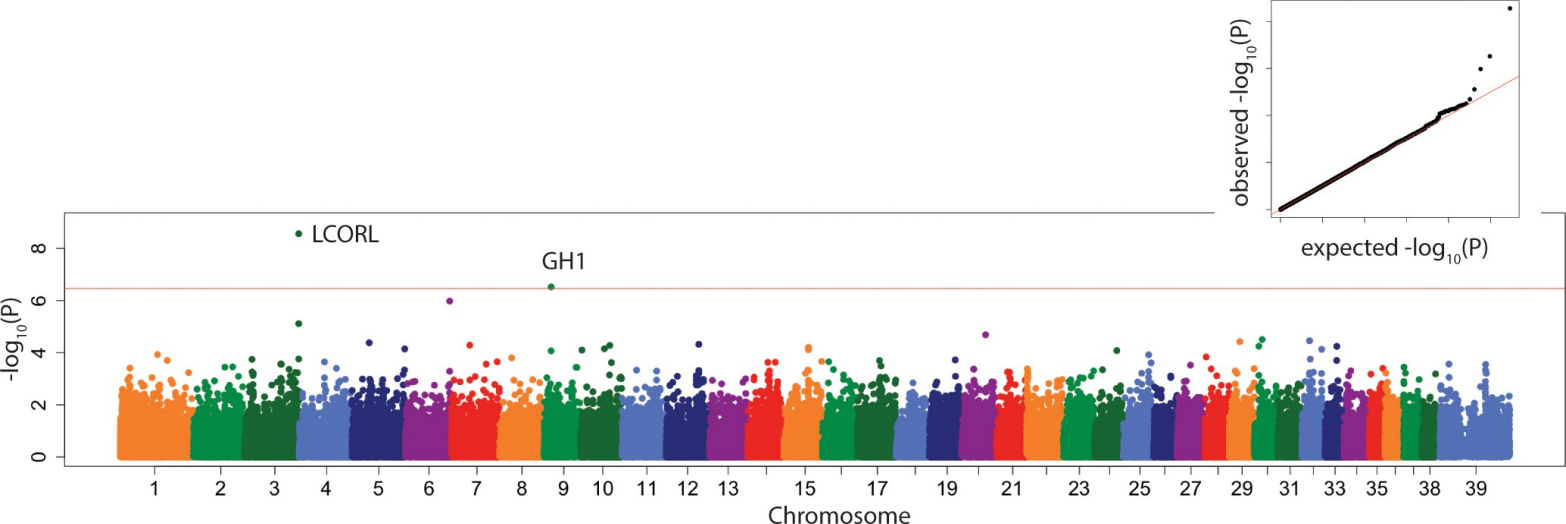
Manhattan plot of linear mixed model of cranial tibial diaphyseal length. Marker position plotted on the X axis against –log_10_(P) on the Y axis. The Bonferroni adjusted genome wide p value threshold is drawn as the red line across the plot. QQ plot of expected –log_10_(P) for no association against observed –log_10_(P) is shown as insert.

Using the multi-locus model, we also saw significant associations on CFA6:74,641,230 with PC1 and the cranial diaphyseal length (Fig 7), on CFA5:29,898,790 with tibial tuberosity length, and on CFA5:12,118,506 with femoral and tibia condyle width (Table 6). All these significant associations are shown in the respective QQ plots as a tail that deviates well above the diagonal X = Y line.

## Discussion

Rupture of the cranial cruciate ligament remains a universal major disorder for mixed and pure breed dogs. In 2005, it was estimated that owners spent $1.32 billion for the treatment of CCLD in the United States,[30]. In 97 primary practices surveyed in England in 2015, ~ 0.5–0.6% of 171,000 dogs suffered from CCLD.[2] Extending this proportion to the approximately 70 million dogs in households in the USA, about 400,000 US dogs might be diagnosed with CCLD annually. In England, about 20% of the affected dogs, especially the heavier, insured dogs, were referred to surgeons for corrective osteotomy procedures. The preference for osteotomy procedures aligns with the preference of USA surgeons.[31] If the approximate total cost of an osteotomy procedure is currently $4,000 in the USA, a likely underestimate, over $3 billion might be spent per year, currently, for surgical correction of CCLD by the American dog-owning public to treat CCLD. This cost ignores the cost of extracapsular and intra-articular repairs, the long-term disability caused by the secondary osteoarthritis, the cost of complications due to the surgical procedures, medical management, and the additional costs for dogs with bilateral CCLD. Therefore, finding a solution to this debilitating trait and its secondary OA is a worthy endeavor.

Because CCLD is a complex trait with heritability in Newfoundland dogs[32] estimated at 0.27, an estimate similar to that of canine hip dysplasia, one approach to reduce the prevalence of the disease is by breeding dogs of improved genetic quality. Unfortunately, CCLD is a late onset disease compared to the earlier age-of-onset of canine hip and elbow dysplasia. Therefore, efforts to reduce the prevalence of the trait require the discovery of other features of the stifle and hind limb that might be used as phenotypic screening tools to find breeding dogs with stifle conformation resistant to CCLD. Similarly, genetic markers and mutations, which are linked or causal for CCLD, will be relevant to improved breeding practices by eliminating dogs with strong genetic susceptibility from the breeding pool. Our goal here was to analyze radiographic measurements of stifle morphology by PCA and to perform a GWAS using published genotypes to find stifle morphologic features that might be under genetic control and thus amenable to practical breeding practices.

Six distinct radiographic dimensions and PC1 in both our GWAS models mapped to the same locus CFA03:91114590 which marks *LCORL*, a size-determining gene in several species. The effect sizes associated with this gene were large (Tables 5 and 6). Multiple measurements mapping to the same locus as PC1 supports the validity of the associations and suggests that *LCORL* is an important determinant of canine distal hind limb morphology. We reported previously that PC1, as a composite average of pelvic radiographic dimensions, mapped to *IGF-1*, derived alleles of which are a major determinant of small body size in dogs.[19, 20, 27, 33] It is unclear why our GWAS of pelvic and stifle morphology showed significant association with two different growth factor-encoding genes, respectively. While IGF-1 is a potent growth factor for chondrocytes and therefore is important for normal growth plate activity, ligand-dependent nuclear receptor corepressor-like (*LCORL*) is a quantitative trait locus for body-size relevant traits in cattle, dogs, and horses. *LCORL* has been linked to arginine metabolism in growth[34] and can interact with C-terminal binding protein 1 (*CTBP1*), another transcriptional regulator.[35] A marker within another C-terminal binding protein (*CTBP2*) has been significantly associated with canine hip dysplasia using two mapping methods.[17,20] The locus CFA24:26793945 also had a large effect size (Table 6). The nearest candidate gene is *XYLT1*, a xylosyltransferase encoding gene, a key conserved regulator of chondrocyte differentiation and skeletal length.[36]

The CFAX:82 locus that was significantly associated with femoral and tibia condyle dimensions in the multi-locus GWAS model, has been fine mapped to the gene insulin receptor substrate 4 (*IRS4*), which also belongs to the IGF-1/growth hormone pathways[37] and acyl-CoA synthetase long-chain family member 4 (*ACSL4*), which has been associated with a muscly or stocky build in pigs and dogs[24, 29, 38, 39]. Other associated loci contain candidate genes which have been associated with skeletal growth and function (Table 6). Variants in *BNC2* (basonuclin 2), a highly conserved protein belonging to the C2H2 zinc finger proteins, have been associated with scoliosis is Japanese [40] and Chinese people.[41] A locus with the nearest candidate gene SEMA6D, an encoding member of the semaphorin family of cell surface or soluble proteins that regulate cell to cell interactions, was associated with tibial diaphyseal width and is expressed in osteoclasts.[42] *FRZB* (frizzled B related protein) is a major component of the WNT signaling pathway and is integral to chondrocyte development.[43]

Growth hormone 1 (*GH1*), the closest candidate gene at CFA09:12074972, which regulates body mass, was associated significantly with the length of the fat pad base and the width of the tibial diaphysis, in the linear mixed model only. The growth hormone-insulin-like growth factor-1 axis is critical to skeletal morphology and endosteal and periosteal osteoblastic activity must be synchronized to increase the width and mass of the diaphyseal cortex.[44–47] *GH1* has been associated with body size in humans and cattle.[33–35]

Fat pad compression, higher body weight, and younger age were all associated with CCLD in this study. Prior studies suggested that the association between body size and CCLD was more prominent within breeds, rather than across breeds,[2] whereas our study included many breeds. Dogs in this study with CCLD were younger than the control population in our study which is not consistent with other reports.[2] For our orthopedic genetic mapping studies, we selected control dogs over 8 years of age as available to allow potential genetic tendencies to be expressed [20], likely explaining the difference to previous reports. Both breed and sex can affect the age at which dogs succumb to CCLD.[48]

Dogs with CCLD had compressed infrapatellar fat pads, a well-recognized radiographic sign of synovitis, effusion, and stifle OA that results from CCLD.[49, 50] A recent study in human medicine found an association between a larger infrapatellar fat pad and decreased knee pain but reduced lateral tibial cartilage volume.[51] However, the infrapatellar fat pad exerts inflammatory effects in the stifle through its release of pro-inflammatory cytokines and alarmins like S100 facilitating the progression of OA. On the contrary, miR-100-5p-abundant exosomes derived from human infrapatellar fat pad mesenchymal stem cells protect articular cartilage via inhibition of mTOR in osteoarthritis which suggests a complex role for this fatty organ in knee/stifle degeneration. [52–54]

In the linear mixed model, PC6 (with a major negative weighting on fat pad base length) was significantly associated with locus CFA35:1,776,352 with the closest candidate gene *FOXQ1* (Fig 5). The volume of the infrapatellar fat pad, including its base length, as well as being related to the size of the dog, is also a function of the extent to which it is compressed by synovial effusion and synovitis that results from CCLD. The human FOXQ1 gene encodes a functional 403 amino acid protein, which has many physiological functions, including promoting epithelial differentiation, inhibiting smooth muscle differentiation, activating T cells and autoimmunity, and controlling mucin gene expression and granule content in stomach surface mucous cells. [55]

In our study, dogs with CCLD had significantly higher TPA, albeit a small elevation on average, compared to control dogs. Our average TPAs were lower than some studies but consistent with others.[56] While many studies have found associations between predictive factors for CCLD, including TPA and intercondylar notch width,[57] another study found that these factors, including TPA, do not predict bilateral rupture.[58] Fifty percent of a group of Labrador retrievers, after initial CCL rupture, tore the contralateral CCL within 5.5 months.[58] At present, clinicians have few useful tools besides body weight regulation, to advise owners about prevention of contralateral CCLD. It may be that a multivariate model which includes TPA might be predictive of CCLD but predicting CCLD risk in an individual dog is difficult with the tools veterinarians now have available.[59, 60] Axial and abaxial[61] TPAs and measures of tibial concavity and femoral condylar convexity might be more sensitive measurements of proclivity for CCLD[62] but require computed tomographic imaging. Lastly, other measures of stifle plateau mechanics, like the anatomic mechanical axis of the tibia, may be a more accurate predictor of CCLD.[12] In this study, even though the anatomic mechanical axis measurement was significantly correlated with the TPA (r = 0.74), the TPA had higher sensitivity and specificity (both above 0.9) and therefore was more accurate for predicting CCLD. One caveat in the referenced study was that CCLD-resistant breeds were compared to CCLD-susceptible breeds possibly exaggerating the potential advantage of this measurement.

Even though no heritability studies of TPA have been reported, the finding of a significant genetic marker association with TPA in the linear mixed model suggests that it may have a genetic basis and may be a path to improve stifle conformation and reduce CCLD risk in dogs. This finding would have to be replicated in order to encourage the application of TPA and/or genetic markers associated with TPA and CCLD in breeding programs to reduce its incidence. Tibial plateau angle was associated with a locus, using the linear mixed model, on CFA10:28,002,796 bp with nearest candidate gene *BET1* (endoplasmic reticulum to Golgi function) and *TXN2* about 50kb upstream and *MYH9* about 80 kb downstream. MYH9-related disease is a rare autosomal-dominant disorder caused by mutations in this gene encoding non-muscle myosin heavy chain IIA. MYH9-related disease has a variable clinical evolution involving thrombocytopenia and possibly sensorineural deafness, cataract, and/or nephropathy often leading to end-stage renal disease. [63] Non-muscle myosin heavy chain IIA was shown to be integral to osteoclastogenesis and functions as a generator of cellular chemomechanical force.[64] Balanced osteoblast-osteoclast activity is important to normal growth plate function and its imbalance might affect TPA. A down-side to this significant association with TPA in our study which contained 77 Labrador retrievers is that we did not replicate the results of Baker *et al*.,[18] who associated a locus on CFA04 with TPA in 237 Labrador Retrievers only. Our study included multiple breeds. Interestingly, rupture of the anterior cruciate ligament in humans was recently associated with several morphological measurements on affected and control stifles including medial tibial posterior slope.[65]

As we have previously discussed[17], we did not obtain (or expect) identical results from application of both models. First of all, the genome wide threshold for the two models differed. The threshold for the FarmCPU method is more stringent at p<0.01 genome-wide. False positives in a GWAS can be effectively controlled by incorporation of genotype-based population structure and kinship among individuals to adjust the effect of a marker association. However, the adjustment for occult population structure can penalize the number of true positive associations. The FarmCPU approach extends previous mixed linear models into two separate stages. The fixed effect component tests each SNP, one at a time, with multiple associated markers (pseudo quantitative trait nucleotides) as covariates to control false positive associations. To avoid model over-fitting, the effect of the associated markers is then estimated as a random effect in the second stage by using them to define kinship. Previously, both real and simulated data analyses demonstrated that FarmCPU improved statistical power compared to current methods, while still controlling inflation [24].

Some limitations of this data included inconsistency of radiographic quality and a relatively small dataset for a PCA and for GWAS. The radiographs varied in positioning because they were used for two different osteotomy surgical planning procedures or for diagnosis of stifle disease, trauma and non-specific lameness. Although fixed traits within and across breeds can be mapped with hundreds of dogs, mapping complex, naturally segregating traits in dogs may require 500–1,000 cases and controls, and denser mapping arrays for adequate mapping power[20]. Because morphology and conformation underlie the fixed traits of breed height and body weight which conform to breed standards, the pure breed dogs in this study may have made identification of morphologic-based, locus associations possible with less that 500–1,000 dogs each group. Although more than two loci likely contribute to TPA, it seems important to replicate the associations of the genomic regions on chromosomes 3 and 10 with TPA in order to understand their effects on CCLD and to select against predisposing risk factors for CCLD.

## Supporting information

S1 File Table A. Excel file of 10 radiographic measurements performed in all 216 dogs with and without cranial cruciate ligament disease (CCLD). Figure A. Distributions of raw (left column) and transformed (right column) phenotypic data. a) tibia length b) lateral diaphysis c) tuberosity length d) fat pad base e) fat pad height f) cranial diaphysis g) femoral condyle h) femoral notch i) tibia condyle. Figure B. Manhattan plot of linear mixed model GWAS of transformed tibial length. Marker position plotted on the X axis against–log_10_(P) on the Y axis. The Bonferroni adjusted genome wide p value threshold is drawn as the red line across the plot. QQ plot of expected–log_10_(P) for no association against observed–log_10_(P) is shown as insert. Figure C. Manhattan plot of linear mixed model GWAS of transformed lateral tibial diaphyseal width. Marker position plotted on the X axis against–log_10_(P) on the Y axis. The Bonferroni adjusted genome wide p value threshold is drawn as the red line across the plot. QQ plot of expected–log_10_(P) for no association against observed–log_10_(P) is shown as insert. Figure D. Manhattan plot linear mixed model GWAS of transformed tuberosity length. Marker position plotted on the X axis against–log_10_(P) on the Y axis. The Bonferroni adjusted genome wide p value threshold is drawn as the red line across the plot. QQ plot of expected–log_10_(P) for no association against observed–log_10_(P) is shown as insert. Figure E. Manhattan plot of linear mixed model GWAS of transformed fat pad base. Marker position plotted on the X axis against–log_10_(P) on the Y axis. The Bonferroni adjusted genome wide p value threshold is drawn as the red line across the plot. QQ plot of expected–log_10_(P) for no association against observed–log_10_(P) is shown as insert. Figure F. Manhattan plot of linear mixed model GWAS of transformed femoral condyle width. Marker position plotted on the X axis against–log_10_(P) on the Y axis. The Bonferroni adjusted genome wide p value threshold is drawn as the red line across the plot. QQ plot of expected–log_10_(P) for no association against observed–log_10_(P) is shown as insert. Figure G. Manhattan plot of linear mixed model GWAS of transformed tibial condyle width. Marker position plotted on the X axis against–log_10_(P) on the Y axis. The Bonferroni adjusted genome wide p value threshold is drawn as the red line across the plot. QQ plot of expected–log_10_(P) for no association against observed–log_10_(P) is shown as insert. Figure H. Manhattan plot of PC1 based on GWAS using FarmCPU modeling software. The Bonferroni adjusted genome wide p value threshold is drawn as the green line across the plot. QQ plot of observed versus expected distribution of p values for PC1 derived from the Farm CPU modeling software. Figure I. Manhattan plot of PC4 based on GWAS using FarmCPU modeling software. The Bonferroni adjusted genome wide p value threshold is drawn as the green line across the plot. QQ plot of observed versus expected distribution of p values for PC4 derived from the Farm CPU modeling software. Figure J. Manhattan plot of PC6 based on GWAS using FarmCPU modeling software. The Bonferroni adjusted genome wide p value threshold is drawn as the green line across the plot. QQ plot of observed versus expected distribution of p values for PC6 derived from the Farm CPU modeling software. Figure K. Manhattan plot of transformed tibial length based on GWAS using FarmCPU modeling software. The Bonferroni adjusted genome wide p value threshold is drawn as the green line across the plot. QQ plot of observed versus expected distribution of p values for tibial length derived from the Farm CPU modeling software. Figure L. Manhattan plot of transformed tibial diaphyseal width based on GWAS using FarmCPU modeling software. The Bonferroni adjusted genome wide p value threshold is drawn as the green line across the plot. QQ plot of observed versus expected distribution of p values for transformed tibial diaphyseal width derived from the Farm CPU modeling software. Figure M. Manhattan plot of transformed tibial tuberosity length based on GWAS using FarmCPU modeling software. The Bonferroni adjusted genome wide p value threshold is drawn as the green line across the plot. QQ plot of observed versus expected distribution of p values for transformed tibial tuberosity length derived from the Farm CPU modeling software. Figure N. Manhattan plot of transformed transformed infrapatellar fat pad height based on GWAS using FarmCPU modeling software. The Bonferroni adjusted genome wide p value threshold is drawn as the green line across the plot. QQ plot of observed versus expected distribution of p values for transformed infrapatellar fat pad height derived from the Farm CPU modeling software. Figure O. Manhattan plot of transformed transformed infrapatellar fat pad width based on GWAS using FarmCPU modeling software. The Bonferroni adjusted genome wide p value threshold is drawn as the green line across the plot. QQ plot of observed versus expected distribution of p values for transformed infrapatellar fat pad width derived from the Farm CPU modeling software.Figure P. Manhattan plot of transformed transformed tibial diaphyseal width on the cranial caudal projection based on GWAS using FarmCPU modeling software. The Bonferroni adjusted genome wide p value threshold is drawn as the green line across the plot. QQ plot of observed versus expected distribution of p values for transformed tibial diaphyseal width on the cranial caudal view derived from the Farm CPU modeling software. Figure Q. Manhattan plot of transformed transformed femoral condyle width on the cranial caudal projection based on GWAS using FarmCPU modeling software. The Bonferroni adjusted genome wide p value threshold is drawn as the green line across the plot. QQ plot of observed versus expected distribution of p values for transformed femoral condyle width on the cranial caudal view derived from the Farm CPU modeling software. Figure R. Manhattan plot of transformed transformed tibial plateau width on the cranial caudal projection based on GWAS using FarmCPU modeling software. The Bonferroni adjusted genome wide p value threshold is drawn as the green line across the plot. QQ plot of observed versus expected distribution of p values for transformed tibial plateau width on the cranial caudal view derived from the Farm CPU modeling software.
(DOCX)
