# Supplementary material for: Genetic mapping of distal femoral, stifle, and tibial radiographic morphology in dogs with cranial cruciate ligament disease
Source: PLoS One. 2019 Oct 17;14(10):e0223094. doi: 10.1371/journal.pone.0223094 (PMC6797204; doi:10.1371/journal.pone.0223094)
Supplement: S1 File — Table A. Excel file of 10 radiographic measurements performed in all 216 dogs with and without cranial cruciate ligament disease (CCLD). Figure A. Distributions of raw (left column) and transformed (right column) phenotypic data. a) tibia length b) lateral diaphysis c) tuberosity length d) fat pad base e) fat pad height f) cranial diaphysis g) femoral condyle h) femoral notch i) tibia condyle. Figure B. Manhattan plot of linear mixed model GWAS of transformed tibial length. Marker position plotted on the X axis against–log10(P) on the Y axis. The Bonferroni adjusted genome wide p value threshold is drawn as the red line across the plot. QQ plot of expected–log10(P) for no association against observed–log10(P) is shown as insert. Figure C. Manhattan plot of linear mixed model GWAS of transformed lateral tibial diaphyseal width. Marker position plotted on the X axis against–log10(P) on the Y axis. The Bonferroni adjusted genome wide p value threshold is drawn as the red line across the plot. QQ plot of expected–log10(P) for no association against observed–log10(P) is shown as insert. Figure D. Manhattan plot linear mixed model GWAS of transformed tuberosity length. Marker position plotted on the X axis against–log10(P) on the Y axis. The Bonferroni adjusted genome wide p value threshold is drawn as the red line across the plot. QQ plot of expected–log10(P) for no association against observed–log10(P) is shown as insert. Figure E. Manhattan plot of linear mixed model GWAS of transformed fat pad base. Marker position plotted on the X axis against–log10(P) on the Y axis. The Bonferroni adjusted genome wide p value threshold is drawn as the red line across the plot. QQ plot of expected–log10(P) for no association against observed–log10(P) is shown as insert. Figure F. Manhattan plot of linear mixed model GWAS of transformed femoral condyle width. Marker position plotted on the X axis against–log10(P) on the Y axis. The Bonferroni adjusted genome wide p value threshold is [file pone.0223094.s001.docx]

Table A in S1 file.

| **pfizerID** | **sex** | **weight^0.303_kgs** | **raw_weight (kg)** | **sex_corr_wt (kg)** | **CCLD disease or not** | **Cornell Veterinary Bioank ID** | **Breed** | **Hospital ID** | **Age** | **Tobial Plateau Angle (degrees)** | **TibiaLength (mm)** | **TibiaLength^1.909 (mm)** | **Lateral Diaphyseal Width (mm)** | **Lateral Diaphyseal Width ^2.030 (mm)** | **Tibial Tuberosity Length (mm)** | **Tibial TuberosityLength^0.99 (mm)** | **Fatpad Base (mm)** | **Fatpad Base^0.727 (mm)** | **Fatpad Height (mm)** | **Fatpad Height^0.091 (mm)** | **Cranial Tibial Diaphysis (mm)** | **Cranial Tibial Diaphysis^1.909 (mm)** | **Femoral Condyle Width (mm)** | **Femoral Condyle Width ^1.667 (mm)** | **Femoral Notch Width (mm)** | **Femoral Notch Width ^1.788 (mm)** | **Tibial Condyle Width (mm)** | **Tibial Condyle Width ^2.03 (mm)** | **PC1_transformed** | **PC2_transformed** | **PC3_transformed** | **PC4_transformed** | **PC5_transformed** | **PC6_transformed** | **PC7_trans formed** | **PC8_tranformed** | **PC9_transformed** | **PC10_tranformed** |
| --- | --- | --- | --- | --- | --- | --- | --- | --- | --- | --- | --- | --- | --- | --- | --- | --- | --- | --- | --- | --- | --- | --- | --- | --- | --- | --- | --- | --- | --- | --- | --- | --- | --- | --- | --- | --- | --- | --- |
| PFZ9H06 | 1 | 2.7297 | 27.4999 | 27.4999 | CCLD+ | 6600 | ChineseSharpei | 209194 | 5y10m | 110.95 | 19.45 | 288.766978 | 1.45 | 2.12606748 | 3.2 | 3.1629948 | 4 | 2.73966596 | 0.6 | 0.95457875 | 1.65 | 2.60121859 | 3.75 | 9.0554518 | 1.05 | 1.09115505 | 4.15 | 17.9737061 | -0.7043451 | -0.3091896 | 1.45327033 | 0.14530412 | -0.3645569 | -0.9061524 | 0.45228795 | -0.1564143 | 0.14302642 | -0.1661277 |
| PFZ9H04 | 2 | 2.0101 | 8.6001 | 10.0165 | CCLD+ | 6132 | WestHighlandWhiteTerrier | 206371 | 8y9m | 122.1 | 8.2 | 55.5227086 | 0.8 | 0.63572995 | 1.1 | 1.09895209 | 1.3 | 1.21014335 | 0.4 | 0.91999922 | 0.9 | 0.81780347 | 1.9 | 2.91529329 | 0.6 | 0.40117553 | 2.1 | 4.50925887 | -6.7939936 | 2.65380989 | -0.4237133 | -0.4811084 | 0.39156887 | 0.31106497 | -0.1800495 | 0.06063744 | -0.1728444 | 0.03482857 |
| PFZ9H01 | 1 | 3.5881 | 67.7984 | 67.7984 | CCLD+ | 489 | Newfoundland | 160918 | 3y0m | 121.7 | 26.4 | 517.418433 | 2.2 | 4.95584873 | 4.2 | 4.14015688 | 4.3 | 2.88756366 | 0.8 | 0.97989872 | 2 | 3.75548699 | 4.8 | 13.6656138 | 1.3 | 1.59856652 | 5.3 | 29.5311266 | 4.15152251 | 1.22087326 | 0.2149778 | -0.2256293 | 0.29781255 | 0.02966182 | -0.9071399 | -0.4652216 | -0.0765759 | -0.0549252 |
| PFZ9G10 | 1 | 3.4401 | 58.9987 | 58.9987 | CCLD+ | 6563 | Rottweiler | 208057 | 3y9m | 114.4 | 22.8 | 391.108832 | 2 | 4.0840485 | 4.2 | 4.14015688 | 4 | 2.73966596 | 1 | 1 | 2 | 3.75548699 | 3.8 | 9.25761798 | 1.1 | 1.18579638 | 4.4 | 20.2399266 | 1.77503333 | -0.5608263 | 0.27561265 | 0.99505088 | 0.5153338 | -0.0880875 | -0.6282045 | 0.64139852 | 0.13611524 | 0.12172612 |
| PFZ9G07 | 2 | 3.1656 | 38.5009 | 44.8420 | CCLD+ | 6136 | Rottweiler | 197679 | 2y6m | 115.4 | 19.9 | 301.654996 | 1.45 | 2.12606748 | 3.5 | 3.4564268 | 3.45 | 2.46033634 | 0.75 | 0.97416063 | 1.7 | 2.75376573 | 3.45 | 7.88033049 | 1 | 1 | 4.05 | 17.1054182 | -0.7495339 | 0.13246151 | 0.24437511 | 0.47357815 | -0.1535295 | -0.208878 | 0.41210512 | 0.29676356 | -0.0013023 | 0.08806765 |
| PFZ9G03 | 2 | 3.1606 | 38.2999 | 44.6079 | CCLD+ | 6058 | Rottweiler | 205386 | 3y7m | 107.1 | 19.1 | 278.928376 | 1.6 | 2.59635196 | 3.1 | 3.0651242 | 3.75 | 2.61409118 | 1.1 | 1.00871095 | 1.7 | 2.75376573 | 3.8 | 9.25761798 | 1.1 | 1.18579638 | 4.3 | 19.3170572 | -0.2149343 | -2.2756383 | 0.64873099 | 0.00440986 | 0.23536704 | -0.2873612 | 0.11409947 | -0.0633174 | 0.18339876 | 0.0892554 |
| PFZ9E09 | 1 | 3.3676 | 54.9980 | 54.9980 | CCLD+ | 6986 | Rottweiler | 209781 | 2y10m | 114.6 | 21.35 | 345.001157 | 1.7 | 2.9363736 | 3.3 | 3.26083482 | 4.3 | 2.88756366 | 1 | 1 | 1.9 | 3.40518431 | 4.1 | 10.5077732 | 1.1 | 1.18579638 | 4.7 | 23.1397494 | 1.2086461 | -0.6963471 | 0.10280213 | 0.21834876 | 0.54753626 | -0.6572335 | 0.3756359 | -0.3087238 | -0.0658553 | 0.12899908 |
| PFZ9E07 | 1 | 3.2954 | 51.2015 | 51.2015 | CCLD+ | 1334 | Rottweiler | 184264 | 3y9m | 121.15 | 25.9 | 498.872138 | 2 | 4.0840485 | 4.9 | 4.822743 | 4.15 | 2.81397974 | 0.85 | 0.9853196 | 2.25 | 4.70236598 | 4.85 | 13.9037349 | 1.2 | 1.38540282 | 5.45 | 31.2525098 | 4.29260514 | 1.12614372 | 0.35340442 | 0.42358732 | 0.63279072 | 0.54716491 | 0.48015764 | 0.03555548 | -0.1164058 | 0.09139443 |
| PFZ9E05 | 1 | 2.6749 | 25.7189 | 25.7189 | CCLD+ | 3499 | Samoyed | 186648 | 11y0m | 119.8 | 19 | 276.147187 | 1.3 | 1.70335435 | 3 | 2.96722201 | 2.8 | 2.11389732 | 0.6 | 0.95457875 | 1.4 | 1.90089628 | 3.4 | 7.69086795 | 1 | 1 | 3.6 | 13.4677199 | -2.0105708 | 1.35818635 | -0.2187053 | -0.1723313 | -0.5066123 | 0.03191191 | 0.1702303 | 0.07874514 | -0.0751551 | -0.2837534 |
| PFZ9E04 | 1 | 3.2974 | 51.3013 | 51.3013 | CCLD+ | 2249 | Rottweiler | 187556 | 4y9m | 116.55 | 22.9 | 394.390037 | 1.9 | 3.68018635 | 4.7 | 4.62782448 | 4.05 | 2.76452042 | 0.8 | 0.97989872 | 1.95 | 3.57829444 | 4.15 | 10.7222561 | 1.2 | 1.38540282 | 4.6 | 22.1512581 | 2.1871237 | 0.27364121 | 0.53322802 | 0.47412083 | -0.1541377 | -0.022549 | -0.1108616 | 0.60658699 | 0.45011141 | -0.0974811 |
| PFZ9D05 | 1 | 2.9341 | 34.8994 | 34.8994 | CCLD+ | 704 | Samoyed | 162982 | 5y3m | 116.1 | 22.25 | 373.29571 | 1.75 | 3.11434871 | 4.05 | 3.9937463 | 3.5 | 2.48620805 | 0.8 | 0.97989872 | 1.65 | 2.60121859 | 3.5 | 8.07163342 | 1 | 1 | 4.05 | 17.1054182 | 0.08292321 | 0.20737881 | 0.10643729 | 0.9675789 | -0.4002702 | 0.13751984 | -0.4491313 | 0.28419029 | 0.13250632 | 0.02014478 |
| PFZ9C11 | 2 | 3.2501 | 41.9981 | 48.9152 | CCLD+ | 7024 | AlaskanMalamute | 203841 | 1y7m | 120.55 | 19.25 | 283.125034 | 1.4 | 1.97988476 | 3.2 | 3.1629948 | 3.25 | 2.35580426 | 0.9 | 0.99045801 | 1.45 | 2.0325981 | 3.6 | 8.45972545 | 0.9 | 0.82829608 | 4 | 16.6794522 | -1.164385 | 0.64828875 | -1.1363251 | 0.44426387 | -0.080374 | 0.05386743 | 0.36950835 | -0.4430325 | 0.14864848 | -0.0590021 |
| PFZ9C08 | 2 | 3.0100 | 32.5997 | 37.9688 | CCLD+ | 2259 | StaffordshireBullTerrier | 189081 | 2y10m | 114.95 | 15.9 | 196.547313 | 1.35 | 1.8389823 | 2.6 | 2.57527502 | 2.85 | 2.14127385 | 0.6 | 0.95457875 | 1.65 | 2.60121859 | 3.25 | 7.13361329 | 0.9 | 0.82829608 | 3.75 | 14.6313188 | -2.3787291 | 0.53162525 | 0.65852172 | 0.05731639 | 0.51177327 | -0.1897422 | 0.36632545 | 0.20635261 | 0.03225336 | 0.05246409 |
| PFZ9B06 | 2 | 2.9875 | 31.8013 | 37.0390 | CCLD+ | 2269 | StaffordshireBullTerrier | 166831 | na | 114.6 | 17.3 | 230.903231 | 1.5 | 2.27753603 | 2.9 | 2.86928718 | 3.3 | 2.38209805 | 0.5 | 0.93887175 | 1.5 | 2.16849394 | 3.3 | 7.31750011 | 1 | 1 | 3.6 | 13.4677199 | -1.9640473 | 0.76421206 | 1.08039181 | 0.02388828 | -0.2324152 | -0.6856194 | -0.1796423 | 0.1717454 | 0.30067904 | -0.2021612 |
| PFZ9A07 | 1 | 3.2718 | 49.9999 | 49.9999 | CCLD- | 7008 | Newfoundland | 211000 | 2y8m | 122.3 | 23.8 | 424.507542 | 1.8 | 3.29763957 | 3.75 | 3.70076029 | 4.25 | 2.86311474 | 1.3 | 1.02416244 | 1.8 | 3.0712503 | 5.05 | 14.8725942 | 1.5 | 2.06467249 | 5.8 | 35.4616408 | 4.04550408 | 0.00292093 | -1.3262001 | -2.0826818 | -0.1369524 | 0.05369246 | 0.52111108 | -0.9325365 | 0.20687587 | 0.42649601 |
| PFZ8H06 | 1 | 2.8847 | 32.9988 | 32.9988 | CCLD+ | 3795 | LabradorRetriever | 186492 | 8y0m | 115.2 | 23 | 397.684294 | 1.8 | 3.29763957 | 4.05 | 3.9937463 | 4.05 | 2.76452042 | 1.15 | 1.01279956 | 1.8 | 3.0712503 | 4.15 | 10.7222561 | 1.25 | 1.49030481 | 4.85 | 24.663558 | 2.04058222 | -0.8674168 | -0.3065707 | -0.1179027 | -0.2027314 | -0.0180669 | -0.0305237 | 0.01708488 | 0.16685979 | 0.24968072 |
| PFZ8H03 | 2 | 3.2665 | 42.6999 | 49.7326 | CCLD+ | 7435 | Rottweiler | 214033 | 2y8m | 116.75 | 20.35 | 314.810686 | 1.75 | 3.11434871 | 3.35 | 3.30974366 | 3.35 | 2.4082833 | 0.85 | 0.9853196 | 2 | 3.75548699 | 3.7 | 8.85507561 | 1.05 | 1.09115505 | 4.25 | 18.863815 | 0.34733322 | 0.19532155 | 0.13325769 | 0.34340933 | 0.98340388 | -0.0043098 | -0.006513 | 0.60330959 | -0.1900779 | 0.10134427 |
| PFZ8G10 | 2 | 3.2734 | 42.9999 | 50.0820 | CCLD+ | 7825 | Rottweiler | 213715 | 2y7m | 110.5 | 21.15 | 338.85781 | 1.6 | 2.59635196 | 3.6 | 3.55418047 | 3.95 | 2.71472654 | 1.15 | 1.01279956 | 1.85 | 3.23616629 | 3.4 | 7.69086795 | 1 | 1 | 3.8 | 15.0300595 | -0.0553006 | -1.7021369 | 0.01994658 | 1.11675679 | 0.1898355 | -0.4350288 | 0.12673724 | 0.61083116 | -0.2023539 | -0.0418488 |
| PFZ8F08 | 2 | n/a | n/a | n/a | CCLD+ | 5340 | Rottweiler | 178848 | na | 116.3 | 23.1 | 400.991595 | 1.7 | 2.9363736 | 4.1 | 4.04255575 | 4.1 | 2.78929125 | 0.9 | 0.99045801 | 2 | 3.75548699 | 3.9 | 9.66728896 | 1 | 1 | 4.6 | 22.1512581 | 1.38304224 | -0.0031738 | 0.19824806 | 1.1204747 | 0.3019015 | -0.1776271 | 0.50408646 | 0.18643593 | -0.2130959 | 0.17441098 |
| PFZ8E11 | 2 | n/a | n/a | n/a | CCLD+ | 5338 | Rottweiler | 167048 | 2y7m | 117.3 | 25.9 | 498.872138 | 2.3 | 5.42384796 | 4.9 | 4.822743 | 5.1 | 3.26891417 | 1 | 1 | 2.3 | 4.90386424 | 4.7 | 13.1943235 | 1.2 | 1.38540282 | 5.3 | 29.5311266 | 5.03447308 | 0.02039327 | 0.64036846 | 1.11631689 | 1.02355012 | -0.3358885 | -0.5912367 | 0.12990667 | 0.24337116 | 0.09964175 |
| PFZ8E10 | 1 | n/a | n/a | n/a | CCLD+ | 3987 | GoldenRetriever | 172118 | 3y2m | 115.5 | 21.5 | 349.643147 | 1.7 | 2.9363736 | 3.4 | 3.3586452 | 3.3 | 2.38209805 | 0.7 | 0.96806367 | 1.5 | 2.16849394 | 3.9 | 9.66728896 | 1.2 | 1.38540282 | 4.3 | 19.3170572 | 0.0287358 | 0.25233284 | 0.45785863 | -0.5766458 | -0.6125301 | 0.04412957 | -0.5080989 | -0.0995511 | 0.12526693 | -0.1311812 |
| PFZ8E07 | 1 | 3.0560 | 39.9161 | 39.9161 | CCLD+ | 8105 | LabradorRetriever | 217001 | 4y1m | 117.25 | 20.25 | 311.864095 | 1.5 | 2.27753603 | 3.4 | 3.3586452 | 3.45 | 2.46033634 | 0.85 | 0.9853196 | 1.7 | 2.75376573 | 3.55 | 8.26476795 | 1.1 | 1.18579638 | 4.15 | 17.9737061 | -0.304516 | 0.1513441 | -0.3061164 | 0.02405039 | -0.1126328 | -0.2121819 | 0.27282329 | 0.32716789 | -0.0537772 | 0.11235313 |
| PFZ8E03 | 2 | 3.0243 | 33.1129 | 38.5666 | CCLD+ | 7924 | GoldenRetriever | 216091 | 3y6m | 125.25 | 18.7 | 267.883292 | 1.4 | 1.97988476 | 3.05 | 3.01617712 | 3.1 | 2.27624996 | 0.7 | 0.96806367 | 1.3 | 1.65012886 | 3.15 | 6.77148145 | 1.1 | 1.18579638 | 3.65 | 13.8501512 | -1.5500778 | 1.91225613 | -1.4762965 | -0.3971955 | -0.6577136 | -0.4229016 | -0.1529198 | 0.19869665 | 0.16962372 | 0.00842508 |
| PFZ8D06 | 2 | 2.7774 | 24.9999 | 29.1174 | CCLD+ | 7248 | BassetHound | 210880 | 3y3m | 118.8 | 10.3 | 85.8037486 | 1.3 | 1.70335435 | 2.7 | 2.67331495 | 2.9 | 2.16851956 | 1.1 | 1.00871095 | 1.9 | 3.40518431 | 3.4 | 7.69086795 | 0.8 | 0.67100367 | 3.5 | 12.7191512 | -2.0891673 | -0.0775261 | -1.2779836 | 0.45907926 | 1.89469101 | -0.0569662 | 1.09251046 | 0.63844201 | 0.5182384 | -0.113528 |
| PFZ8C05 | 2 | 3.0755 | 34.9999 | 40.7644 | CCLD+ | 7556 | Rottweiler | 181768 | 8y0m | 118.2 | 18.8 | 270.624639 | 1.6 | 2.59635196 | 3.1 | 3.0651242 | 3.5 | 2.48620805 | 0.55 | 0.94705021 | 1.7 | 2.75376573 | 3.6 | 8.45972545 | 1 | 1 | 4.1 | 17.5368355 | -0.8021956 | 1.27365691 | 0.63460915 | 0.1475137 | 0.21095373 | -0.5787761 | 0.04956303 | 0.01145787 | 0.17190511 | -0.0366374 |
| PFZ8B05 | 2 | 3.0486 | 33.9999 | 39.5997 | CCLD+ | 8115 | GoldenRetriever | 215951 | 5y6m | 115.85 | 19.2 | 281.722832 | 1.45 | 2.12606748 | 3 | 2.96722201 | 3.3 | 2.38209805 | 0.9 | 0.99045801 | 1.5 | 2.16849394 | 3.3 | 7.31750011 | 1.05 | 1.09115505 | 3.6 | 13.4677199 | -1.3548874 | -0.281631 | -0.5398946 | 0.08357712 | -0.2384335 | -0.3273825 | -0.0836519 | 0.25492711 | -0.0414017 | -0.124995 |
| PFZ8A02 | 2 | 3.2025 | 39.9999 | 46.5879 | CCLD- | 3098 | Newfoundland | 186217 | 2y2m | 119.5 | 24.4 | 445.171321 | 1.6 | 2.59635196 | 3.65 | 3.60304707 | 3.6 | 2.53765114 | 1.25 | 1.02051363 | 1.8 | 3.0712503 | 3.7 | 8.85507561 | 1.1 | 1.18579638 | 4.1 | 17.5368355 | 0.8593584 | -0.2452905 | -1.4579676 | 0.56341754 | -0.1173083 | 0.12524553 | 0.06216559 | 0.33773787 | -0.7874535 | -0.0896258 |
| PFZ7C01 | 1 | 3.2397 | 48.3983 | 48.3983 | CCLD+ | 7928 | LabradorRetriever | 216282 | 3y9m | 116.7 | 22.15 | 370.09946 | 1.9 | 3.68018635 | 4 | 3.94493082 | 3.85 | 2.66458728 | 1 | 1 | 1.7 | 2.75376573 | 4.1 | 10.5077732 | 1.3 | 1.59856652 | 4.7 | 23.1397494 | 1.81302904 | -0.3072956 | -0.2625229 | -0.4736106 | -0.3056571 | -0.0385378 | -0.5762864 | 0.14294834 | 0.47249159 | 0.13586973 |
| PFZ7A02 | 2 | 3.3971 | 48.5978 | 56.6019 | CCLD+ | 792 | Rottweiler | 181068 | 5y10m | 114.7 | 22 | 365.329631 | 1.9 | 3.68018635 | 3.4 | 3.3586452 | 3.3 | 2.38209805 | 0.8 | 0.97989872 | 2.1 | 4.12208206 | 4.3 | 11.3760611 | 1.2 | 1.38540282 | 4.9 | 25.1824536 | 1.68596521 | 0.0007402 | 0.96789328 | -0.4692926 | 1.10790587 | 0.38978778 | -0.1124777 | 0.29304272 | -0.2779912 | 0.1602509 |
| PFZ5H06 | 2 | 3.2360 | 41.3999 | 48.2185 | CCLD+ | 6126 | LabradorRetriever | 200068 | 6y4m | 116.2 | 21.1 | 337.330187 | 1.8 | 3.29763957 | 3.1 | 3.0651242 | 3.8 | 2.63938458 | 0.7 | 0.96806367 | 1.9 | 3.40518431 | 3.8 | 9.25761798 | 1.1 | 1.18579638 | 4.3 | 19.3170572 | 0.48239647 | 0.4136975 | 0.6422034 | 0.11193386 | 0.60028766 | -0.6289691 | -0.3045079 | 0.18243549 | -0.200141 | -0.0192782 |
| PFZ5H05 | 1 | 2.9367 | 34.9999 | 34.9999 | CCLD+ | 4826 | GermanShepherd | 194951 | 6y5m | 121.7 | 22.9 | 394.390037 | 1.8 | 3.29763957 | 3.9 | 3.84728147 | 3.5 | 2.48620805 | 0.9 | 0.99045801 | 1.7 | 2.75376573 | 4.2 | 10.9384697 | 0.9 | 0.82829608 | 4.5 | 21.1846554 | 0.90846898 | 1.02538944 | -0.7473298 | 0.95679504 | 0.26508178 | 0.53850277 | -0.1022325 | -0.6761316 | 0.10437959 | -0.2231791 |
| PFZ5H04 | 1 | 3.0532 | 39.7982 | 39.7982 | CCLD+ | 7050 | LabradorRetriever | 211628 | 4y3m | 115.1 | 20.4 | 316.288928 | 1.8 | 3.29763957 | 3 | 2.96722201 | 3.9 | 2.68970078 | 0.9 | 0.99045801 | 1.8 | 3.0712503 | 3.6 | 8.45972545 | 1.1 | 1.18579638 | 4.2 | 18.4160319 | 0.26182434 | -0.3783657 | 0.05308742 | 0.18554224 | 0.54741278 | -0.7273618 | -0.4841625 | 0.14105459 | -0.0507447 | 0.13419908 |
| PFZ5G12 | 2 | 3.2860 | 43.5459 | 50.7179 | CCLD+ | 3958 | GermanShepherd | 159715 | 5y9m | 117.4 | 23.2 | 404.311936 | 2 | 4.0840485 | 4.6 | 4.53033433 | 3.8 | 2.63938458 | 0.8 | 0.97989872 | 1.9 | 3.40518431 | 4.4 | 11.8204936 | 1.1 | 1.18579638 | 4.9 | 25.1824536 | 2.31451404 | 0.53504866 | 0.5452617 | 0.62541782 | 0.18474402 | 0.50369619 | -0.3112851 | -0.1073741 | 0.53866709 | -0.0174276 |
| PFZ5G05 | 1 | 3.2876 | 50.7999 | 50.7999 | CCLD+ | 4093 | LabradorRetriever | 186072 | 7y11m | 118.4 | 21.4 | 346.545204 | 1.6 | 2.59635196 | 3.7 | 3.65190698 | 3.6 | 2.53765114 | 0.7 | 0.96806367 | 1.8 | 3.0712503 | 3.8 | 9.25761798 | 1.1 | 1.18579638 | 4.3 | 19.3170572 | 0.33752173 | 0.82636065 | 0.19311976 | 0.172511 | -0.1068884 | -0.2151839 | 0.27292644 | 0.29388937 | -0.0469584 | -0.0450018 |
| PFZ5F11 | 1 | 2.8847 | 32.9988 | 32.9988 | CCLD+ | 867 | LabradorRetriever | 181405 | 6y7m | 118.1 | 20.6 | 322.234862 | 1.9 | 3.68018635 | 3.8 | 3.74960708 | 3.7 | 2.58870554 | 0.8 | 0.97989872 | 1.9 | 3.40518431 | 4.3 | 11.3760611 | 1.2 | 1.38540282 | 4.7 | 23.1397494 | 1.60204952 | 0.51209187 | 0.26068849 | -0.3054974 | 0.55028993 | -0.0192524 | -0.1922643 | 0.12483789 | 0.50038468 | -0.0792095 |
| PFZ5F05 | 1 | 3.1475 | 43.9984 | 43.9984 | CCLD+ | 58 | LabradorRetriever | 170520 | 6y1m | 121.1 | 24.15 | 436.504586 | 1.9 | 3.68018635 | 3.55 | 3.50530708 | 4 | 2.73966596 | 0.65 | 0.96155718 | 1.95 | 3.57829444 | 4.55 | 12.4998557 | 1.35 | 1.71016013 | 5.2 | 28.4110175 | 2.74711576 | 1.43059932 | 0.50334504 | -1.1009127 | 0.11546768 | -0.3493921 | -0.1370096 | -0.2378083 | -0.2636543 | 0.11118656 |
| PFZ5F03 | 1 | 3.2052 | 46.7200 | 46.7200 | CCLD+ | 860 | GermanShepherd | 181499 | 3y6m | 113.7 | 26.05 | 504.402177 | 2.15 | 4.72987947 | 4.55 | 4.48158133 | 4.55 | 3.00866813 | 1.15 | 1.01279956 | 1.95 | 3.57829444 | 4.45 | 12.0452589 | 1.15 | 1.28388977 | 5 | 26.2366939 | 3.50251173 | -0.9809901 | 0.33587952 | 1.01427217 | 0.14350386 | 0.11064183 | -0.8331636 | -0.3202617 | 0.0858265 | 0.03998653 |
| PFZ5E05 | 1 | 2.9865 | 36.9999 | 36.9999 | CCLD+ | 6693 | LabradorRetriever | 208959 | 7y7m | 115.1 | 22.5 | 381.343569 | 1.6 | 2.59635196 | 3 | 2.96722201 | 3.3 | 2.38209805 | 0.6 | 0.95457875 | 1.6 | 2.45281643 | 3.4 | 7.69086795 | 1.1 | 1.18579638 | 4.1 | 17.5368355 | -0.6825702 | 0.53129832 | 0.7985924 | -0.0756834 | -0.5007687 | -0.2953562 | -0.4396601 | 0.08297417 | -0.5942261 | 0.11379401 |
| PFZ5E04 | 2 | 3.1681 | 38.6007 | 44.9582 | CCLD+ | 490 | LabradorRetriever | 125607 | na | 119.3 | 20 | 304.55537 | 1.6 | 2.59635196 | 3.3 | 3.26083482 | 3.4 | 2.43436207 | 0.8 | 0.97989872 | 1.8 | 3.0712503 | 4.1 | 10.5077732 | 1.2 | 1.38540282 | 4.5 | 21.1846554 | 0.52523698 | 0.64737489 | -0.2166333 | -0.7234618 | 0.28134323 | -0.0808172 | 0.35272298 | 0.12345966 | 0.07059205 | -0.0800265 |
| PFZ5E02 | 1 | 3.1573 | 44.4529 | 44.4529 | CCLD+ | 4819 | GoldenRetriever | 186040 | 9y1m | 120.3 | 19 | 276.147187 | 1.6 | 2.59635196 | 3.1 | 3.0651242 | 3.4 | 2.43436207 | 0.7 | 0.96806367 | 1.6 | 2.45281643 | 3.7 | 8.85507561 | 1 | 1 | 4.3 | 19.3170572 | -0.5849167 | 1.14604682 | -0.2656667 | 0.0338992 | 0.21139196 | -0.2625209 | 0.05431272 | -0.3317655 | 0.24913792 | 0.10781234 |
| PFZ5E01 | 2 | 2.8744 | 28.0002 | 32.6119 | CCLD+ | 7662 | GermanShepherd | 214639 | 2y9m | 110.2 | 21.5 | 349.643147 | 1.65 | 2.7637096 | 3.75 | 3.70076029 | 3.75 | 2.61409118 | 1.1 | 1.00871095 | 1.5 | 2.16849394 | 4.1 | 10.5077732 | 1.2 | 1.38540282 | 4.8 | 24.1501433 | 0.79066172 | -1.6997079 | 0.2652118 | -0.3401563 | -0.6689881 | 0.19873942 | 0.02769202 | -0.5077186 | 0.41343243 | 0.26333548 |
| PFZ5D12 | 2 | 2.7398 | 23.9000 | 27.8363 | CCLD+ | 331 | LabradorRetriever | 177861 | 0y7m | 121.4 | 19.8 | 298.767841 | 1.55 | 2.43429579 | 2.75 | 2.72232121 | 3 | 2.2226299 | 0.9 | 0.99045801 | 1.3 | 1.65012886 | 4 | 10.0840271 | 1.1 | 1.18579638 | 4.4 | 20.2399266 | -0.6233168 | 0.74440457 | -1.1935317 | -0.8325844 | -0.1393309 | 0.28312594 | -0.1584676 | -0.8971437 | 0.1335656 | -0.0488964 |
| PFZ5D04 | 2 | 3.0621 | 34.4999 | 40.1821 | CCLD+ | 6143 | LabradorRetriever | 195984 | 4y0m | 118.4 | 21.1 | 337.330187 | 1.6 | 2.59635196 | 3.5 | 3.4564268 | 3.2 | 2.3293998 | 0.7 | 0.96806367 | 1.55 | 2.30857087 | 3.45 | 7.88033049 | 1.05 | 1.09115505 | 4.05 | 17.1054182 | -0.619198 | 0.8382201 | -0.077575 | 0.21167635 | -0.4417971 | 0.04196312 | -0.2570807 | 0.15410514 | -0.0082846 | 0.06962521 |
| PFZ5C07 | 1 | 3.2499 | 48.9018 | 48.9018 | CCLD+ | 2610 | GermanShepherd | 179769 | 5y9m | 122.15 | 23.7 | 421.109067 | 2.1 | 4.50925887 | 4.2 | 4.14015688 | 4.35 | 2.91193509 | 0.85 | 0.9853196 | 1.9 | 3.40518431 | 4.5 | 12.271715 | 1.15 | 1.28388977 | 4.85 | 24.663558 | 2.90188079 | 1.14140633 | -0.2901407 | 0.45365161 | 0.42441799 | -0.2338596 | -0.6674217 | -0.3761513 | 0.43780048 | -0.2007803 |
| PFZ5C05 | 2 | 3.2265 | 41.0002 | 47.7529 | CCLD+ | 1095 | LabradorRetriever | 182761 | 7y0m | 118.2 | 21 | 334.284803 | 1.7 | 2.9363736 | 3.1 | 3.0651242 | 3.4 | 2.43436207 | 0.6 | 0.95457875 | 1.8 | 3.0712503 | 4.1 | 10.5077732 | 1.1 | 1.18579638 | 4.7 | 23.1397494 | 0.3877008 | 1.14459518 | 0.74406104 | -0.4022087 | 0.38934589 | -0.0952939 | 0.07787144 | -0.3530607 | -0.1166008 | 0.07623005 |
| PFZ5C04 | 2 | 3.0513 | 34.0999 | 39.7162 | CCLD+ | 6551 | LabradorRetriever | 157985 | 8y10m | 112 | 18.5 | 262.440484 | 1.45 | 2.12606748 | 2.9 | 2.86928718 | 3.2 | 2.3293998 | 0.4 | 0.91999922 | 1.7 | 2.75376573 | 3.75 | 9.0554518 | 1.05 | 1.09115505 | 4.2 | 18.4160319 | -1.3702687 | 0.81831399 | 2.28420322 | -0.4130828 | -0.0773419 | -0.4076239 | 0.34901627 | 0.00482876 | -0.0040532 | -0.130922 |
| PFZ5C01 | 1 | 3.0555 | 39.8980 | 39.8980 | CCLD+ | 7378 | LabradorRetriever | 211321 | 2y1m | 114.15 | 20.1 | 307.468956 | 1.5 | 2.27753603 | 3.25 | 3.21191858 | 3.35 | 2.4082833 | 0.75 | 0.97416063 | 1.55 | 2.30857087 | 3.2 | 6.95160383 | 1 | 1 | 3.65 | 13.8501512 | -1.3237555 | -0.1280966 | 0.22983797 | 0.48659339 | -0.3887548 | -0.3086092 | -0.161452 | 0.32921657 | -0.089552 | -0.0422197 |
| PFZ5B06 | 2 | 3.1379 | 37.3999 | 43.5597 | CCLD+ | 6175 | LabradorRetriever | 205027 | 4y5m | 110.9 | 17.4 | 233.457868 | 1.4 | 1.97988476 | 2.6 | 2.57527502 | 2.95 | 2.19563733 | 0.7 | 0.96806367 | 1.4 | 1.90089628 | 3.4 | 7.69086795 | 0.9 | 0.82829608 | 3.9 | 15.8438657 | -2.322226 | -0.5448124 | 0.81173443 | 0.0774086 | -0.0101864 | 0.00254842 | 0.02852199 | -0.4156015 | 0.10153671 | 0.04787572 |
| PFZ5B05 | 1 | 3.2854 | 50.6889 | 50.6889 | CCLD+ | 4135 | GermanShepherd | 197008 | 3y10m | 123.05 | 21.85 | 360.589272 | 1.75 | 3.11434871 | 3.95 | 3.89610924 | 3.7 | 2.58870554 | 1 | 1 | 1.85 | 3.23616629 | 4.2 | 10.9384697 | 1.1 | 1.18579638 | 4.65 | 22.6427668 | 1.53205676 | 0.93471243 | -1.1319223 | 0.23116015 | 0.39219479 | 0.17512562 | 0.27163836 | -0.081223 | 0.20165926 | -0.0259422 |
| PFZ5B04 | 1 | 2.9367 | 34.9999 | 34.9999 | CCLD+ | 4853 | LabradorRetriever | 155085 | 10y4m | 110.2 | 18.1 | 251.714596 | 1.5 | 2.27753603 | 3.2 | 3.1629948 | 3 | 2.2226299 | 0.7 | 0.96806367 | 1.5 | 2.16849394 | 3.6 | 8.45972545 | 1 | 1 | 4 | 16.6794522 | -1.5298289 | -0.6403166 | 1.07434611 | 0.01658659 | -0.2000757 | 0.21618844 | 0.00742724 | -0.0382408 | 0.34538076 | -0.06086 |
| PFZ5B03 | 2 | 2.9931 | 32.0009 | 37.2715 | CCLD+ | 249 | LabradorRetriever | 156706 | 12y0m | 112.7 | 19.6 | 293.033208 | 1.5 | 2.27753603 | 3.8 | 3.74960708 | 3.6 | 2.53765114 | 0.6 | 0.95457875 | 1.8 | 3.0712503 | 3.8 | 9.25761798 | 1.1 | 1.18579638 | 4.4 | 20.2399266 | -0.1175524 | 0.12392121 | 1.39653615 | 0.06274243 | -0.2502258 | -0.2483639 | 0.62573071 | 0.36300685 | 0.28775461 | 0.05633964 |
| PFZ5B02 | 1 | 2.9865 | 36.9995 | 36.9995 | CCLD+ | 870 | LabradorRetriever | 181524 | 4y1m | 117.95 | 19.85 | 300.209766 | 1.55 | 2.43429579 | 3.45 | 3.40753954 | 3.4 | 2.43436207 | 0.6 | 0.95457875 | 1.7 | 2.75376573 | 4 | 10.0840271 | 1.05 | 1.09115505 | 4.35 | 19.7757604 | -0.2192914 | 1.07489931 | 0.57815893 | -0.0776662 | -0.0204944 | -0.0956241 | 0.39494446 | -0.1532606 | 0.20859816 | -0.1956844 |
| PFZ5B01 | 2 | 3.0459 | 33.9015 | 39.4850 | CCLD+ | 7327 | LabradorRetriever | 212992 | 5y5m | 115.65 | 19.05 | 277.536123 | 1.5 | 2.27753603 | 3.35 | 3.30974366 | 3.3 | 2.38209805 | 0.75 | 0.97416063 | 1.45 | 2.0325981 | 3.45 | 7.88033049 | 0.95 | 0.9123675 | 4 | 16.6794522 | -1.2569073 | 0.16990061 | 0.05438951 | 0.42671967 | -0.3357816 | -0.0643975 | 0.02845 | -0.1850505 | 0.3216586 | 0.05888328 |
| PFZ5A08 | 1 | 2.9865 | 36.9999 | 36.9999 | CCLD+ | 6541 | GoldenRetriever | 208661 | 4y11m | 116.6 | 21.05 | 335.805851 | 1.6 | 2.59635196 | 3.8 | 3.74960708 | 3.45 | 2.46033634 | 1 | 1 | 1.55 | 2.30857087 | 3.8 | 9.25761798 | 1.1 | 1.18579638 | 4.25 | 18.863815 | 0.12751318 | -0.2916201 | -0.5916864 | 0.15920153 | -0.4442229 | 0.21655295 | 0.01730773 | -0.0139642 | 0.27885532 | -0.0210755 |
| PFZ5A07 | 1 | 3.0011 | 37.5999 | 37.5999 | CCLD+ | 6618 | LabradorRetriever | 209190 | 6y11m | 118.35 | 21.3 | 343.460393 | 1.45 | 2.12606748 | 3.25 | 3.21191858 | 3.45 | 2.46033634 | 0.65 | 0.96155718 | 1.5 | 2.16849394 | 3.2 | 6.95160383 | 0.95 | 0.9123675 | 3.75 | 14.6313188 | -1.2697857 | 0.93588459 | -0.0662735 | 0.64300553 | -0.6031345 | -0.4385874 | -0.0430965 | 0.02572879 | -0.2866246 | -0.017587 |
| PFZ5A05 | 2 | 3.1927 | 39.5986 | 46.1205 | CCLD+ | 129 | LabradorRetriever | 176606 | 6y4m | 119.25 | 22.15 | 370.09946 | 1.7 | 2.9363736 | 3.3 | 3.26083482 | 3.45 | 2.46033634 | 0.75 | 0.97416063 | 1.85 | 3.23616629 | 4.05 | 10.2950278 | 1.1 | 1.18579638 | 4.65 | 22.6427668 | 0.76629674 | 0.87566393 | 0.07862407 | -0.1266383 | 0.39480918 | 0.0413205 | 0.11875252 | -0.1687979 | -0.3172431 | 0.09096863 |
| PFZ5A04 | 2 | 3.2963 | 43.9984 | 51.2450 | CCLD+ | 477 | LabradorRetriever | 177492 | na | 118.1 | 22 | 365.329631 | 1.8 | 3.29763957 | 3.7 | 3.65190698 | 3.4 | 2.43436207 | 0.7 | 0.96806367 | 1.9 | 3.40518431 | 4 | 10.0840271 | 1.2 | 1.38540282 | 4.7 | 23.1397494 | 1.17468222 | 0.83308709 | 0.50029414 | -0.3375579 | 0.22822554 | 0.12934336 | -0.1307418 | 0.33535601 | -0.0483293 | 0.19841285 |
| PFZ5A02 | 1 | 2.9006 | 33.5999 | 33.5999 | CCLD+ | 6473 | LabradorRetriever | 180193 | 5y8m | 116.45 | 16.8 | 218.331007 | 1.3 | 1.70335435 | 2.8 | 2.77131856 | 2.9 | 2.16851956 | 0.45 | 0.92991304 | 1.4 | 1.90089628 | 3.1 | 6.59325608 | 0.8 | 0.67100367 | 3.6 | 13.4677199 | -2.9242345 | 1.40270832 | 0.92487311 | 0.4298303 | -0.2294553 | -0.2871768 | 0.20340078 | -0.1550444 | 0.16517528 | -0.029868 |
| PFZ5A01 | 2 | 3.2170 | 40.6010 | 47.2880 | CCLD+ | 7142 | LabradorRetriever | 209166 | 7y0m | 113.45 | 19.35 | 285.93938 | 1.4 | 1.97988476 | 2.8 | 2.77131856 | 3.1 | 2.27624996 | 0.7 | 0.96806367 | 1.65 | 2.60121859 | 3.7 | 8.85507561 | 1 | 1 | 4.25 | 18.863815 | -1.2028964 | -0.0716729 | 0.70289868 | -0.1668034 | 0.15056652 | 0.01353078 | 0.43134021 | -0.2339036 | -0.243528 | 0.06121272 |
| PFZ4H11 | 1 | 3.2875 | 50.7978 | 50.7978 | CCLD+ | 5917 | GermanShepherd | 204025 | 4y3m | 122.5 | 23.5 | 414.351151 | 1.6 | 2.59635196 | 4.4 | 4.33528996 | 3.8 | 2.63938458 | 1.2 | 1.01672966 | 1.7 | 2.75376573 | 4 | 10.0840271 | 1.2 | 1.38540282 | 4.8 | 24.1501433 | 1.78584508 | 0.3725689 | -1.6609428 | 0.09212087 | -0.6338088 | 0.29782491 | 0.48752811 | 0.03531149 | 0.08931516 | 0.33252908 |
| PFZ4H09 | 2 | 2.9988 | 32.2005 | 37.5039 | CCLD+ | 1316 | LabradorRetriever | 184125 | 2y8m | 114.5 | 21.1 | 337.330187 | 1.6 | 2.59635196 | 3.4 | 3.3586452 | 3.4 | 2.43436207 | 0.6 | 0.95457875 | 1.8 | 3.0712503 | 3.9 | 9.66728896 | 1.2 | 1.38540282 | 4.5 | 21.1846554 | 0.2333326 | 0.42934773 | 1.17751254 | -0.5675096 | -0.1728866 | -0.1624138 | 0.18253098 | 0.28758321 | -0.1320342 | 0.05530351 |
| PFZ4H04 | 1 | 3.1277 | 43.0912 | 43.0912 | CCLD+ | 195 | GoldenRetriever | 170268 | 4y8m | 110.1 | 22.2 | 371.695949 | 1.7 | 2.9363736 | 3.6 | 3.55418047 | 3.5 | 2.48620805 | 0.7 | 0.96806367 | 1.8 | 3.0712503 | 4.1 | 10.5077732 | 1.2 | 1.38540282 | 4.8 | 24.1501433 | 0.81386475 | -0.6379981 | 1.61878865 | -0.4180128 | -0.2172484 | 0.15817412 | 0.05226486 | -0.0218384 | -0.085379 | 0.18995953 |
| PFZ4H03 | 2 | 3.0349 | 33.4978 | 39.0149 | CCLD+ | 278 | LabradorRetriever | 177477 | na | 116.2 | 19 | 276.147187 | 1.6 | 2.59635196 | 2.8 | 2.77131856 | 3.3 | 2.38209805 | 0.8 | 0.97989872 | 1.6 | 2.45281643 | 3.6 | 8.45972545 | 1 | 1 | 4.2 | 18.4160319 | -0.8885513 | 0.115639 | -0.0283856 | -0.0015419 | 0.34720833 | -0.1987922 | -0.1148695 | -0.2939948 | 0.03408359 | 0.14285226 |
| PFZ4G11 | 1 | 3.1035 | 41.9999 | 41.9999 | CCLD+ | 6183 | LabradorRetriever | 205950 | 2y1m | 110.7 | 20 | 304.55537 | 1.6 | 2.59635196 | 3 | 2.96722201 | 3.1 | 2.27624996 | 0.5 | 0.93887175 | 1.7 | 2.75376573 | 3.8 | 9.25761798 | 1.1 | 1.18579638 | 4.5 | 21.1846554 | -0.7263366 | 0.17748216 | 2.08917481 | -0.5016732 | -0.0488179 | 0.00268315 | -0.0239672 | -0.092684 | -0.0956408 | 0.1616168 |
| PFZ4G09 | 2 | 3.0072 | 32.4999 | 37.8526 | CCLD+ | 1272 | LabradorRetriever | 173725 | 12y6m | 116.9 | 21.5 | 349.643147 | 1.7 | 2.9363736 | 3.3 | 3.26083482 | 3.9 | 2.68970078 | 0.7 | 0.96806367 | 1.7 | 2.75376573 | 4 | 10.0840271 | 1.3 | 1.59856652 | 4.7 | 23.1397494 | 0.97300023 | 0.4123146 | 0.45120131 | -0.9525834 | -0.367211 | -0.6533349 | -0.1138858 | -0.0389397 | 0.04989357 | 0.17274994 |
| PFZ4G06 | 1 | 2.7711 | 28.8999 | 28.8999 | CCLD+ | 829 | GoldenRetriever | 176500 | 8y1m | 114.7 | 22.65 | 386.211504 | 1.65 | 2.7637096 | 4 | 3.94493082 | 3.5 | 2.48620805 | 0.8 | 0.97989872 | 1.55 | 2.30857087 | 3.8 | 9.25761798 | 1.2 | 1.38540282 | 4.5 | 21.1846554 | 0.55059087 | -0.1434628 | 0.3213662 | -0.1512538 | -0.9763807 | 0.18580398 | -0.1770661 | 0.08496458 | 0.13549023 | 0.16479561 |
| PFZ4G01 | 1 | 3.1146 | 42.5016 | 42.5016 | CCLD+ | 72 | GoldenRetriever | 176144 | na | 114.1 | 23.7 | 421.109067 | 1.7 | 2.9363736 | 3.5 | 3.4564268 | 3.7 | 2.58870554 | 0.9 | 0.99045801 | 1.9 | 3.40518431 | 4.6 | 12.7296747 | 1.4 | 1.82505908 | 4.9 | 25.1824536 | 2.15744408 | -0.5827606 | 0.55374092 | -1.3973784 | -0.1682032 | 0.05182557 | 0.23855616 | 0.08472587 | -0.4121184 | -0.2361531 |
| PFZ4F12 | 1 | 2.9865 | 36.9995 | 36.9995 | CCLD+ | 6999 | LabradorRetriever | 210882 | 5y3m | 109.15 | 18.65 | 266.517603 | 1.5 | 2.27753603 | 2.75 | 2.72232121 | 3.45 | 2.46033634 | 1.05 | 1.00444978 | 1.7 | 2.75376573 | 3.5 | 8.07163342 | 1.1 | 1.18579638 | 4.2 | 18.4160319 | -0.8044916 | -1.8117618 | 0.34050788 | -0.2177441 | 0.33225513 | -0.3044664 | 0.16004053 | 0.13512961 | -0.1184423 | 0.2937173 |
| PFZ4F09 | 2 | 3.2277 | 41.0501 | 47.8110 | CCLD+ | 2056 | GermanShepherd | 187722 | 7y11m | 117.9 | 21.3 | 343.460393 | 1.6 | 2.59635196 | 3.5 | 3.4564268 | 3.3 | 2.38209805 | 1.15 | 1.01279956 | 1.6 | 2.45281643 | 3.85 | 9.46156628 | 0.95 | 0.9123675 | 4.25 | 18.863815 | -0.0644149 | -0.2940182 | -1.0865891 | 0.59949898 | 0.13970357 | 0.47843796 | 0.10301312 | -0.3838021 | 0.00095782 | -0.0366621 |
| PFZ4F04 | 1 | 3.5752 | 67.0001 | 67.0001 | CCLD+ | 223 | GermanShepherd | 172723 | 3y1m | 120.75 | 27.75 | 569.100599 | 2.1 | 4.50925887 | 5.9 | 5.79620172 | 3.75 | 2.61409118 | 0.85 | 0.9853196 | 2 | 3.75548699 | 4.8 | 13.6656138 | 1.1 | 1.18579638 | 5.3 | 29.5311266 | 4.19136593 | 1.20865627 | 0.28710925 | 1.29163118 | -0.4019021 | 1.5435129 | -0.2018605 | -0.1553651 | 0.25978453 | -0.0815798 |
| PFZ4E12 | 1 | 3.1573 | 44.4529 | 44.4529 | CCLD+ | 3760 | GermanShepherd | 194225 | 6y3m | 119 | 22 | 365.329631 | 1.6 | 2.59635196 | 3.5 | 3.4564268 | 3.6 | 2.53765114 | 0.9 | 0.99045801 | 1.6 | 2.45281643 | 3.9 | 9.66728896 | 0.9 | 0.82829608 | 4.3 | 19.3170572 | -0.0062003 | 0.43457017 | -0.6077364 | 0.82225324 | -0.0258155 | 0.09686114 | 0.16730994 | -0.6427647 | -0.0674103 | -0.1161739 |
| PFZ4E07 | 1 | 3.2049 | 46.7018 | 46.7018 | CCLD+ | 1136 | GoldenRetriever | 182971 | 7y0m | 120.5 | 25.25 | 475.244368 | 1.85 | 3.48625065 | 4.75 | 4.67656175 | 3.35 | 2.4082833 | 0.75 | 0.97416063 | 2.05 | 3.93675258 | 4.3 | 11.3760611 | 1.35 | 1.71016013 | 4.8 | 24.1501433 | 2.78066533 | 1.18308186 | 0.22878031 | -0.4297031 | -0.3408619 | 0.70851875 | -0.005627 | 1.04436345 | -0.2751312 | -0.0659115 |
| PFZ4E01 | 2 | 3.2265 | 41.0002 | 47.7529 | CCLD+ | 1207 | GoldenRetriever | 183259 | 5y0m | 119.1 | 23.6 | 417.723601 | 1.7 | 2.9363736 | 3.8 | 3.74960708 | 3.6 | 2.53765114 | 0.6 | 0.95457875 | 1.5 | 2.16849394 | 3.8 | 9.25761798 | 1.1 | 1.18579638 | 4.3 | 19.3170572 | 0.30532119 | 1.27708268 | 0.32115083 | 0.22857752 | -0.9776096 | -0.1109459 | -0.4369802 | -0.2579293 | -0.0783277 | -0.1237607 |
| PFZ4D12 | 1 | 3.1410 | 43.6991 | 43.6991 | CCLD+ | 492 | LabradorRetriever | 178934 | na | 116.95 | 23.205 | 404.478296 | 1.75 | 3.11434871 | 3.6 | 3.55418047 | 3.5 | 2.48620805 | 0.7 | 0.96806367 | 1.65 | 2.60121859 | 4.15 | 10.7222561 | 1.25 | 1.49030481 | 4.55 | 21.6652216 | 0.99421342 | 0.53071422 | 0.46095619 | -0.6320953 | -0.5670236 | 0.04739594 | -0.3679773 | -0.0987381 | -0.1103649 | -0.1685269 |
| PFZ4D08 | 2 | 2.9557 | 30.6991 | 35.7552 | CCLD+ | 1976 | GoldenRetriever | 154117 | 7y5m | 113.25 | 19.75 | 297.329222 | 1.45 | 2.12606748 | 3.45 | 3.40753954 | 2.75 | 2.086387 | 0.65 | 0.96155718 | 1.55 | 2.30857087 | 3.6 | 8.45972545 | 1 | 1 | 4.1 | 17.5368355 | -1.3191302 | 0.13542124 | 0.85507736 | 0.05129801 | -0.3691949 | 0.59317218 | 0.17158737 | 0.0982644 | 0.06138098 | -0.0004855 |
| PFZ4D06 | 2 | 3.2734 | 42.9999 | 50.0820 | CCLD+ | 6543 | LabradorRetriever | 208665 | 4y4m | 117.5 | 19.4 | 287.351522 | 1.6 | 2.59635196 | 3.6 | 3.55418047 | 3.2 | 2.3293998 | 0.8 | 0.97989872 | 1.6 | 2.45281643 | 3.8 | 9.25761798 | 1.1 | 1.18579638 | 4.3 | 19.3170572 | -0.2379427 | 0.37843335 | -0.1457502 | -0.1685336 | -0.1411605 | 0.2219929 | 0.06837396 | 0.07018521 | 0.41903286 | 0.03225257 |
| PFZ4D04 | 2 | 3.2218 | 40.8006 | 47.5205 | CCLD+ | 478 | GoldenRetriever | 178268 | na | 114.4 | 22.2 | 371.695949 | 1.7 | 2.9363736 | 3.4 | 3.3586452 | 4 | 2.73966596 | 0.9 | 0.99045801 | 1.8 | 3.0712503 | 4.1 | 10.5077732 | 1.2 | 1.38540282 | 4.4 | 20.2399266 | 1.02236423 | -0.5306242 | 0.25254721 | -0.1990282 | -0.0233396 | -0.4804031 | 0.00626768 | 0.01359369 | -0.1541072 | -0.2249231 |
| PFZ4D02 | 2 | 3.2758 | 43.1003 | 50.1989 | CCLD+ | 1572 | LabradorRetriever | 185480 | 4y9m | 120.4 | 22.6 | 384.585592 | 2 | 4.0840485 | 3.7 | 3.65190698 | 4.1 | 2.78929125 | 1 | 1 | 2.1 | 4.12208206 | 4.5 | 12.271715 | 1.3 | 1.59856652 | 5 | 26.2366939 | 3.02818817 | 0.40952031 | -0.3433553 | -0.5387958 | 0.99838525 | -0.2602519 | -0.1875959 | 0.19579657 | 0.04740846 | 0.05049637 |
| PFZ4C07 | 2 | 3.0295 | 33.2999 | 38.7844 | CCLD+ | 6619 | LabradorRetriever | 209288 | 2y5m | 110.8 | 19.85 | 300.209766 | 1.5 | 2.27753603 | 3.25 | 3.21191858 | 3.15 | 2.30288246 | 0.65 | 0.96155718 | 1.5 | 2.16849394 | 3.5 | 8.07163342 | 0.9 | 0.82829608 | 3.95 | 16.2589353 | -1.5480284 | -0.3386396 | 1.16407733 | 0.57674908 | -0.335448 | 0.11971018 | -0.0274285 | -0.2172617 | 0.05348027 | -0.0648612 |
| PFZ4C06 | 2 | 3.0241 | 33.1077 | 38.5605 | CCLD+ | 6174 | LabradorRetriever | 162018 | 9y11m | 116 | 20.1 | 307.468956 | 1.5 | 2.27753603 | 3.1 | 3.0651242 | 3.1 | 2.27624996 | 0.6 | 0.95457875 | 1.5 | 2.16849394 | 3.7 | 8.85507561 | 1.1 | 1.18579638 | 4 | 16.6794522 | -1.0490407 | 0.66847543 | 0.58468328 | -0.409818 | -0.478122 | -0.0697572 | -0.0807546 | -0.0315266 | -0.0234033 | -0.2306349 |
| PFZ4C04 | 1 | 3.1626 | 44.7015 | 44.7015 | CCLD+ | 685 | LabradorRetriever | 180464 | 6y6m | 123.3 | 25.3 | 477.042503 | 2 | 4.0840485 | 4 | 3.94493082 | 4.3 | 2.88756366 | 1 | 1 | 2.05 | 3.93675258 | 4.85 | 13.9037349 | 1.2 | 1.38540282 | 5.4 | 30.6732174 | 3.79701313 | 1.01779255 | -0.6009338 | -0.0328957 | 0.72634784 | 0.10404659 | 0.09115139 | -0.747349 | -0.181135 | 0.06587382 |
| PFZ4C02 | 1 | 2.9417 | 35.1987 | 35.1987 | CCLD+ | 1068 | LabradorRetriever | 148290 | 7y6m | 116.1 | 19.4 | 287.351522 | 1.8 | 3.29763957 | 2.8 | 2.77131856 | 3.4 | 2.43436207 | 0.6 | 0.95457875 | 1.7 | 2.75376573 | 4.1 | 10.5077732 | 1.1 | 1.18579638 | 4.8 | 24.1501433 | 0.13444689 | 0.74426335 | 1.02944547 | -0.6331298 | 0.5744634 | -0.1875188 | -0.3102757 | -0.6749069 | 0.23998847 | 0.22860805 |
| PFZ4B12 | 1 | 3.0463 | 39.4988 | 39.4988 | CCLD+ | 6984 | GermanShepherd | 210412 | 7y0m | 114.3 | 19.8 | 298.767841 | 1.5 | 2.27753603 | 3.5 | 3.4564268 | 3.6 | 2.53765114 | 0.8 | 0.97989872 | 1.5 | 2.16849394 | 3.5 | 8.07163342 | 1 | 1 | 3.9 | 15.8438657 | -0.8963663 | -0.2578382 | 0.11606511 | 0.5055486 | -0.5005118 | -0.3094987 | 0.07343577 | -0.0151235 | 0.26517154 | -0.0978894 |
| PFZ4B09 | 2 | n/a | n/a | n/a | CCLD+ | 3290 | GermanShepherd | 194303 | 4y11m | 114.9 | 22.8 | 391.108832 | 1.6 | 2.59635196 | 4.1 | 4.04255575 | 3.7 | 2.58870554 | 0.7 | 0.96806367 | 1.6 | 2.45281643 | 4.3 | 11.3760611 | 1 | 1 | 4.8 | 24.1501433 | 0.710607 | 0.27449158 | 0.82015324 | 0.48629169 | -0.6424205 | 0.36552676 | 0.47778194 | -0.828413 | 0.24252775 | -0.0755106 |
| PFZ4B08 | 2 | 3.0241 | 33.1077 | 38.5605 | CCLD+ | 6056 | LabradorRetriever | 204094 | 4y9m | 115.9 | 19.65 | 294.461905 | 1.6 | 2.59635196 | 3.2 | 3.1629948 | 3.1 | 2.27624996 | 0.9 | 0.99045801 | 1.7 | 2.75376573 | 3.4 | 7.69086795 | 1 | 1 | 4 | 16.6794522 | -0.7942635 | -0.1286337 | -0.2555468 | 0.33204396 | 0.33155326 | 0.14369529 | -0.110237 | 0.31003719 | -0.072718 | 0.16096159 |
| PFZ4B06 | 2 | 2.9051 | 28.9981 | 33.7741 | CCLD+ | 7046 | LabradorRetriever | 211076 | 6y4m | 110.75 | 18.6 | 265.155238 | 1.45 | 2.12606748 | 3 | 2.96722201 | 3.5 | 2.48620805 | 0.75 | 0.97416063 | 1.5 | 2.16849394 | 3.4 | 7.69086795 | 1.1 | 1.18579638 | 3.85 | 15.4342411 | -1.340126 | -0.8353399 | 0.71886934 | -0.1693426 | -0.4487088 | -0.5633466 | -0.0106466 | 0.16227389 | 0.14561429 | -0.0300979 |
| PFZ4B04 | 2 | 3.0755 | 34.9992 | 40.7635 | CCLD+ | 130 | LabradorRetriever | 173327 | na | 113.6 | 20.9 | 331.252573 | 1.7 | 2.9363736 | 2.8 | 2.77131856 | 3.2 | 2.3293998 | 0.7 | 0.96806367 | 1.8 | 3.0712503 | 4.4 | 11.8204936 | 1.1 | 1.18579638 | 4.5 | 21.1846554 | 0.19348224 | -0.0110624 | 1.05513658 | -0.5904066 | 0.64237229 | 0.20984394 | 0.00799289 | -0.4927463 | -0.2517355 | -0.4234046 |
| PFZ4A12 | 1 | 3.0509 | 39.6984 | 39.6984 | CCLD+ | 6088 | GermanShepherd | 199035 | 1y2m | 116.15 | 20.85 | 329.741392 | 1.5 | 2.27753603 | 3.05 | 3.01617712 | 3.2 | 2.3293998 | 0.75 | 0.97416063 | 1.6 | 2.45281643 | 3.4 | 7.69086795 | 0.9 | 0.82829608 | 3.85 | 15.4342411 | -1.2510091 | 0.3026343 | 0.04031997 | 0.64447298 | -0.0125442 | -0.0334462 | -0.0158173 | -0.0889792 | -0.3594433 | -0.0521937 |
| PFZ4A09 | 1 | 2.8688 | 32.4001 | 32.4001 | CCLD+ | 6667 | LabradorRetriever | 209735 | 7y5m | 116.8 | 20 | 304.55537 | 1.5 | 2.27753603 | 2.7 | 2.67331495 | 3.55 | 2.51197905 | 0.95 | 0.99534319 | 1.65 | 2.60121859 | 3.9 | 9.66728896 | 1.25 | 1.49030481 | 4.3 | 19.3170572 | -0.0175883 | -0.3150817 | -0.5225489 | -1.0705161 | 0.02643521 | -0.5094526 | 0.17392382 | -0.027626 | -0.2205237 | -0.0573171 |
| PFZ4A08 | 1 | 3.3565 | 54.3993 | 54.3993 | CCLD+ | 4044 | GermanShepherd | 194933 | 2y5m | 120.1 | 26.3 | 513.683391 | 2.2 | 4.95584873 | 4.45 | 4.38405921 | 4 | 2.73966596 | 1.3 | 1.02416244 | 2.05 | 3.93675258 | 4.6 | 12.7296747 | 1.2 | 1.38540282 | 4.85 | 24.663558 | 3.84572663 | -0.05726 | -0.8451691 | 0.62359784 | 0.69435011 | 0.60275558 | -0.9865158 | 0.03906312 | -0.1526082 | -0.2543094 |
| PFZ4A07 | 1 | 3.2518 | 48.9999 | 48.9999 | CCLD+ | 5297 | LabradorRetriever | 199607 | 6y4m | 117.15 | 19.85 | 300.209766 | 1.75 | 3.11434871 | 3.8 | 3.74960708 | 3.3 | 2.38209805 | 0.85 | 0.9853196 | 1.85 | 3.23616629 | 4.05 | 10.2950278 | 1.15 | 1.28388977 | 4.25 | 18.863815 | 0.62868223 | 0.22308769 | 0.01946126 | -0.1184607 | 0.40142283 | 0.25600579 | -0.0151684 | 0.51317126 | 0.38197425 | -0.2591891 |
| PFZ44F02 | 2 | 3.3167 | 44.9056 | 52.3016 | CCLD+ | 2969 | SaintBernard | 193275 | 9y4m | 120 | 22.35 | 376.505045 | 1.7 | 2.9363736 | 3.9 | 3.84728147 | 3.65 | 2.56322608 | 0.85 | 0.9853196 | 1.7 | 2.75376573 | 4.1 | 10.5077732 | 1.2 | 1.38540282 | 4.4 | 20.2399266 | 1.08995048 | 0.65931527 | -0.4843184 | -0.19745 | -0.3715398 | 0.00225702 | -0.022698 | 0.10190759 | 0.13064815 | -0.2355598 |
| PFZ42H02 | 2 | 3.3323 | 45.6041 | 53.1151 | CCLD+ | 1099 | BerneseMountainDog | 182719 | 2y7m | 118.6 | 23.1 | 400.991595 | 2 | 4.0840485 | 4.4 | 4.33528996 | 3.7 | 2.58870554 | 0.9 | 0.99045801 | 2 | 3.75548699 | 4.1 | 10.5077732 | 1.1 | 1.18579638 | 4.9 | 25.1824536 | 2.25253315 | 0.51011375 | 0.08968804 | 0.67911723 | 0.56188056 | 0.45013166 | -0.3362726 | 0.24221822 | 0.25303475 | 0.34754422 |
| PFZ42C04 | 1 | 3.4022 | 56.8804 | 56.8804 | CCLD+ | 8301 | CaneCorso | 218743 | 5y3m | 111.6 | 22.5 | 381.343569 | 1.75 | 3.11434871 | 4.05 | 3.9937463 | 4.2 | 2.83858716 | 0.9 | 0.99045801 | 1.9 | 3.40518431 | 3.9 | 9.66728896 | 1.1 | 1.18579638 | 4.5 | 21.1846554 | 1.24825672 | -0.9251327 | 0.79896605 | 0.74255569 | -0.0456783 | -0.3550617 | 0.12852026 | 0.25721069 | 0.05328202 | 0.07670825 |
| PFZ42C03 | 1 | 3.1542 | 44.3103 | 44.3103 | CCLD+ | 2545 | BerneseMountainDog | 191140 | 1y5m | 115.5 | 24.5 | 448.660726 | 1.8 | 3.29763957 | 3.7 | 3.65190698 | 4.1 | 2.78929125 | 0.7 | 0.96806367 | 1.8 | 3.0712503 | 4.1 | 10.5077732 | 1.2 | 1.38540282 | 4.7 | 23.1397494 | 1.58413287 | 0.27858494 | 0.84213416 | 0.00252211 | -0.4333251 | -0.4221697 | -0.256432 | -0.1717662 | -0.3554158 | 0.00326889 |
| PFZ41H07 | 2 | 3.3055 | 44.4067 | 51.7204 | CCLD+ | 2678 | GermanShepherd | 191844 | 7y1m | 120.5 | 21.1 | 337.330187 | 1.9 | 3.68018635 | 3.6 | 3.55418047 | 3.2 | 2.3293998 | 0.5 | 0.93887175 | 1.8 | 3.0712503 | 3.9 | 9.66728896 | 0.9 | 0.82829608 | 4.3 | 19.3170572 | 0.08524509 | 2.10167149 | 0.85214976 | 0.73512661 | 0.57305447 | 0.21984185 | -0.5743463 | -0.1521809 | 0.20181097 | -0.160829 |
| PFZ41G09 | 1 | 2.8055 | 30.0999 | 30.0999 | CCLD+ | 6246 | Mixed | 182949 | 3y9m | 122.3 | 21.4 | 346.545204 | 1.75 | 3.11434871 | 3.5 | 3.4564268 | 3.75 | 2.61409118 | 0.8 | 0.97989872 | 1.7 | 2.75376573 | 4.1 | 10.5077732 | 1.1 | 1.18579638 | 4.7 | 23.1397494 | 0.97123481 | 1.229055 | -0.6001583 | -0.0690345 | 0.16478695 | -0.1816313 | -0.0077513 | -0.4907789 | 0.21390464 | 0.09966087 |
| PFZ41F11 | 1 | 2.9723 | 36.4234 | 36.4234 | CCLD+ | 7679 | LabradorRetriever | 214776 | 2y2m | 113.7 | 19.9 | 301.654996 | 1.65 | 2.7637096 | 2.8 | 2.77131856 | 3.6 | 2.53765114 | 1.2 | 1.01672966 | 1.6 | 2.45281643 | 3.8 | 9.25761798 | 1 | 1 | 4.3 | 19.3170572 | -0.286844 | -1.2512476 | -0.5976578 | 0.20219858 | 0.49904051 | -0.1355338 | -0.1328947 | -0.5883305 | -0.0081682 | 0.10032449 |
| PFZ41E11 | 1 | 3.2895 | 50.8999 | 50.8999 | CCLD+ | 7666 | LabradorRetriever | 214710 | 4y10m | 111.9 | 22.4 | 378.114617 | 1.7 | 2.9363736 | 4 | 3.94493082 | 3.7 | 2.58870554 | 0.9 | 0.99045801 | 1.6 | 2.45281643 | 3.8 | 9.25761798 | 1.1 | 1.18579638 | 4.4 | 20.2399266 | 0.48666892 | -0.8710446 | 0.49088165 | 0.45473822 | -0.655014 | 0.12630557 | -0.2067794 | -0.0408225 | 0.19961885 | 0.08372895 |
| PFZ41D07 | 2 | 2.9333 | 29.9371 | 34.8677 | CCLD+ | 2336 | LabradorRetriever | 175672 | 6y6m | 113.4 | 18.9 | 273.379273 | 1.3 | 1.70335435 | 2.7 | 2.67331495 | 2.7 | 2.05873978 | 0.5 | 0.93887175 | 1.7 | 2.75376573 | 3.6 | 8.45972545 | 0.9 | 0.82829608 | 4.1 | 17.5368355 | -1.9864796 | 0.71114766 | 1.48412029 | -0.0348697 | 0.25451026 | 0.23170363 | 0.60031105 | -0.1045424 | -0.4292036 | -0.0283401 |
| PFZ41B09 | 1 | 3.0857 | 41.2134 | 41.2134 | CCLD+ | 4293 | LabradorRetriever | 196510 | 4y8m | 118.75 | 21.3 | 343.460393 | 1.55 | 2.43429579 | 3.9 | 3.84728147 | 3.75 | 2.61409118 | 0.9 | 0.99045801 | 1.8 | 3.0712503 | 3.6 | 8.45972545 | 1.1 | 1.18579638 | 4 | 16.6794522 | 0.32192436 | 0.3086429 | -0.5814912 | 0.49345766 | -0.1931775 | -0.3201071 | 0.34096761 | 0.64622897 | -0.0170055 | -0.1048626 |
| PFZ41A11 | 2 | 2.9021 | 28.8999 | 33.6598 | CCLD+ | 7426 | LabradorRetriever | 213625 | 4y8m | 114.2 | 18.3 | 257.050901 | 1.6 | 2.59635196 | 3.2 | 3.1629948 | 3.2 | 2.3293998 | 0.8 | 0.97989872 | 1.4 | 1.90089628 | 3.5 | 8.07163342 | 1 | 1 | 3.9 | 15.8438657 | -1.3031524 | -0.2495457 | 0.0910227 | 0.15682369 | -0.2160862 | -0.0106785 | -0.354174 | -0.1529302 | 0.48352662 | -0.0433938 |
| PFZ41A08 | 2 | 3.0521 | 34.1283 | 39.7492 | CCLD+ | 2777 | LabradorRetriever | 191835 | 7y8m | 117.5 | 19.2 | 281.722832 | 1.7 | 2.9363736 | 2.6 | 2.57527502 | 3.1 | 2.27624996 | 0.7 | 0.96806367 | 1.5 | 2.16849394 | 3.4 | 7.69086795 | 1.2 | 1.38540282 | 3.6 | 13.4677199 | -1.0945037 | 0.52511588 | -0.1036006 | -0.8050528 | -0.0745466 | -0.4837142 | -0.9672441 | 0.39592229 | -0.0450767 | -0.232875 |
| PFZ41A07 | 2 | 3.7249 | 65.8616 | 76.7090 | CCLD+ | 2161 | Rottweiler | 160211 | 3y8m | 115.7 | 25.3 | 477.042503 | 2.1 | 4.50925887 | 4.8 | 4.72529389 | 4.6 | 3.03266852 | 0.9 | 0.99045801 | 2.3 | 4.90386424 | 5.1 | 15.1188752 | 1.3 | 1.59856652 | 5.6 | 33.0233931 | 4.92252873 | -0.0548579 | 1.17556079 | 0.01471681 | 0.80263266 | 0.17888315 | 0.32955021 | -0.0361388 | 0.16760641 | 0.00768905 |
| PFZ40E02 | 2 | 3.4265 | 50.0000 | 58.2350 | CCLD+ | 3163 | Newfoundland | 179209 | 1y8m | 126.15 | 27.05 | 542.010023 | 1.85 | 3.48625065 | 4.35 | 4.28651516 | 4.2 | 2.83858716 | 1.05 | 1.00444978 | 1.85 | 3.23616629 | 3.95 | 9.8747784 | 1.2 | 1.38540282 | 4.75 | 23.6422077 | 2.80116095 | 1.36502726 | -1.663101 | 0.59174346 | -0.5337067 | -0.1070174 | -0.2744835 | 0.08391231 | -0.653164 | 0.22800137 |
| PFZ38H02 | 2 | 2.8664 | 27.7417 | 32.3107 | CCLD+ | 8474 | LabradorRetriever | 220511 | 9y10m | 114.5 | 20 | 304.55537 | 1.5 | 2.27753603 | 3 | 2.96722201 | 3.2 | 2.3293998 | 0.5 | 0.93887175 | 1.7 | 2.75376573 | 3.4 | 7.69086795 | 0.9 | 0.82829608 | 3.9 | 15.8438657 | -1.5017253 | 0.87863585 | 1.32847556 | 0.52004226 | 0.05715949 | -0.2856243 | 0.08818141 | 0.07144492 | -0.2991823 | -0.0631477 |
| PFZ38H01 | 1 | 3.1237 | 42.9098 | 42.9098 | CCLD+ | 8959 | LabradorRetriever | 222004 | 6y11m | 113.2 | 21.35 | 345.001157 | 1.65 | 2.7637096 | 3.3 | 3.26083482 | 3.65 | 2.56322608 | 1 | 1 | 1.8 | 3.0712503 | 3.85 | 9.46156628 | 1.15 | 1.28388977 | 4.4 | 20.2399266 | 0.48984486 | -0.9243921 | 0.11006136 | -0.0868639 | 0.18589649 | -0.1566787 | 0.06191032 | 0.14575695 | -0.1936175 | 0.08917317 |
| PFZ38G09 | 2 | 3.2364 | 41.4129 | 48.2337 | CCLD+ | 9371 | LabradorRetriever | 223038 | 4y8m | 118.8 | 20.2 | 310.395749 | 1.5 | 2.27753603 | 3 | 2.96722201 | 3.4 | 2.43436207 | 1 | 1 | 1.5 | 2.16849394 | 3.7 | 8.85507561 | 1.1 | 1.18579638 | 4.2 | 18.4160319 | -0.4518796 | 0.01951695 | -1.0549082 | -0.2922692 | -0.1446221 | -0.1499369 | 0.07151127 | -0.2851884 | -0.0417295 | 0.04737223 |
| PFZ38F03 | 2 | 3.1606 | 38.2999 | 44.6079 | CCLD+ | 8300 | LabradorRetriever | 218746 | 12y5m | 120.3 | 22.2 | 371.695949 | 1.6 | 2.59635196 | 4.35 | 4.28651516 | 3.5 | 2.48620805 | 0.85 | 0.9853196 | 1.8 | 3.0712503 | 3.8 | 9.25761798 | 1.15 | 1.28388977 | 4.45 | 20.7095576 | 0.9541513 | 0.7942192 | -0.5072967 | 0.2528762 | -0.4202898 | 0.24847828 | 0.40054386 | 0.57027536 | 0.12179331 | 0.12993781 |
| PFZ38D04 | 1 | 3.0809 | 40.9999 | 40.9999 | CCLD+ | 7847 | LabradorRetriever | 215118 | 4y11m | 114.1 | 21.8 | 359.015704 | 1.6 | 2.59635196 | 3.9 | 3.84728147 | 3.6 | 2.53765114 | 1 | 1 | 1.7 | 2.75376573 | 4.1 | 10.5077732 | 1.2 | 1.38540282 | 4.6 | 22.1512581 | 0.91846645 | -0.7501121 | -0.0043767 | -0.2507543 | -0.4091734 | 0.23578251 | 0.32143607 | 0.03446924 | 0.15874689 | 0.02028152 |
| PFZ38C04 | 2 | 2.8992 | 28.8039 | 33.5480 | CCLD+ | 7860 | LabradorRetriever | 215879 | 4y10m | 118.35 | 18.4 | 259.739036 | 1.6 | 2.59635196 | 2.8 | 2.77131856 | 3.05 | 2.24949993 | 0.8 | 0.97989872 | 1.65 | 2.60121859 | 3.45 | 7.88033049 | 1.1 | 1.18579638 | 3.95 | 16.2589353 | -0.9612149 | 0.47728973 | -0.4067527 | -0.4058046 | 0.38432535 | -0.1705736 | -0.2519092 | 0.31550622 | 0.00232374 | 0.06959875 |
| PFZ38C03 | 2 | 3.0868 | 35.4255 | 41.2601 | CCLD+ | 8440 | LabradorRetriever | 219674 | 7y9m | 117.6 | 18.5 | 262.440484 | 1.6 | 2.59635196 | 3 | 2.96722201 | 2.9 | 2.16851956 | 0.9 | 0.99045801 | 1.7 | 2.75376573 | 3.8 | 9.25761798 | 1 | 1 | 4.3 | 19.3170572 | -0.6546314 | 0.19645505 | -0.39946 | -0.1227497 | 0.7183127 | 0.43567477 | 0.11319625 | -0.1212553 | 0.1106584 | 0.09781602 |
| PFZ38C02 | 1 | 2.7809 | 29.2385 | 29.2385 | CCLD+ | 8861 | GoldenRetriever | 221211 | 6y9m | 115.25 | 18.05 | 250.388851 | 1.3 | 1.70335435 | 3.1 | 3.0651242 | 3.05 | 2.24949993 | 0.8 | 0.97989872 | 1.4 | 1.90089628 | 3.25 | 7.13361329 | 1 | 1 | 3.6 | 13.4677199 | -1.9751574 | -0.1071144 | -0.2631373 | 0.07664863 | -0.4643582 | -0.0865389 | 0.21264214 | 0.18397086 | 0.14731548 | -0.0846333 |
| PFZ38B04 | 1 | 3.1035 | 41.9999 | 41.9999 | CCLD+ | 8104 | LabradorRetriever | 210106 | 6y6m | 109.8 | 19.4 | 287.351522 | 1.6 | 2.59635196 | 3.1 | 3.0651242 | 3.1 | 2.27624996 | 0.9 | 0.99045801 | 1.5 | 2.16849394 | 3.5 | 8.07163342 | 1 | 1 | 4 | 16.6794522 | -1.2085581 | -1.2539838 | 0.53809146 | 0.2174665 | -0.0782795 | 0.23660637 | -0.3427615 | -0.0924277 | 0.12544723 | 0.06748614 |
| PFZ38B03 | 1 | 2.7895 | 29.5379 | 29.5379 | CCLD+ | 8449 | GoldenRetriever | 219751 | 7y4m | 115.7 | 20.3 | 313.335741 | 1.4 | 1.97988476 | 3 | 2.96722201 | 2.9 | 2.16851956 | 1.05 | 1.00444978 | 1.5 | 2.16849394 | 3.55 | 8.26476795 | 1.2 | 1.38540282 | 4.05 | 17.1054182 | -0.8139648 | -0.6386997 | -0.8119924 | -0.7987647 | -0.3978335 | 0.29251128 | 0.04850275 | 0.27271192 | -0.2719608 | 0.07696733 |
| PFZ38A01 | 1 | 3.1281 | 43.1094 | 43.1094 | CCLD+ | 9544 | Rottweiler | 223761 | 4y9m | 116.55 | 21.65 | 354.314669 | 1.7 | 2.9363736 | 3.95 | 3.89610924 | 3.35 | 2.4082833 | 1.15 | 1.01279956 | 1.7 | 2.75376573 | 3.8 | 9.25761798 | 1.15 | 1.28388977 | 4.1 | 17.5368355 | 0.58566186 | -0.5772726 | -0.8291685 | 0.19427337 | -0.1633312 | 0.39920154 | -0.1998436 | 0.4922232 | 0.14171421 | -0.1362628 |
| PFZ37H10 | 2 | 3.5818 | 57.8783 | 67.4109 | CCLD+ | 8871 | Newfoundland | 221353 | 6y0m | 113.2 | 23.05 | 399.336314 | 1.75 | 3.11434871 | 3.35 | 3.30974366 | 3.9 | 2.68970078 | 0.9 | 0.99045801 | 1.75 | 2.9104466 | 3.7 | 8.85507561 | 0.95 | 0.9123675 | 4.3 | 19.3170572 | 0.32135251 | -0.611184 | 0.37465373 | 0.99718955 | 0.1390917 | -0.2693558 | -0.3004586 | -0.3226919 | -0.3858692 | 0.07452328 |
| PFZ36B12 | 2 | n/a | n/a | n/a | CCLD+ | 3998 | GoldenRetriever | 177734 | 6y6m | 118.9 | 21.6 | 352.754214 | 1.85 | 3.48625065 | 3.65 | 3.60304707 | 3.65 | 2.56322608 | 0.9 | 0.99045801 | 1.75 | 2.9104466 | 4.05 | 10.2950278 | 1.15 | 1.28388977 | 4.65 | 22.6427668 | 1.19662311 | 0.39066045 | -0.3261262 | -0.072406 | 0.22487502 | 0.03156343 | -0.352385 | -0.1431041 | 0.25562292 | 0.13253951 |
| PFZ35H09 | 1 | 0.0000 | 79.0000 | 79.0000 | CCLD+ | 5361 | Newfoundland | 178000 | na | 125 | 28.1 | 582.881625 | 2.2 | 4.95584873 | 5.1 | 5.01758195 | 4.2 | 2.83858716 | 1 | 1 | 2.2 | 4.50489733 | 4.7 | 13.1943235 | 1.5 | 2.06467249 | 5.4 | 30.6732174 | 5.58216898 | 1.34236999 | -0.6464086 | -0.5987111 | -0.0597553 | 0.3807241 | -0.6900666 | 0.80402205 | -0.3092965 | 0.19732211 |
| PFZ35F03 | 1 | 2.8817 | 32.8854 | 32.8854 | CCLD+ | 1930 | AmericanPitBullTerrier | 186615 | 2y4m | 122.6 | 19.9 | 301.654996 | 1.6 | 2.59635196 | 3.7 | 3.65190698 | 3.6 | 2.53765114 | 0.7 | 0.96806367 | 1.85 | 3.23616629 | 3.95 | 9.8747784 | 1.25 | 1.49030481 | 4.4 | 20.2399266 | 0.79405826 | 1.49842422 | -0.3882706 | -0.6646804 | 0.02755125 | -0.4039292 | 0.42191577 | 0.61135144 | 0.24715101 | -0.0671368 |
| PFZ35D03 | 1 | 3.1689 | 44.9963 | 44.9963 | CCLD+ | 1186 | BouvierDesFlandres | 166425 | 4y2m | 117.35 | 26.3 | 513.683391 | 1.95 | 3.87945091 | 5.05 | 4.96887949 | 4.9 | 3.17521071 | 0.8 | 0.97989872 | 1.95 | 3.57829444 | 5.05 | 14.8725942 | 1.55 | 2.18933932 | 5.7 | 34.2315021 | 5.06349622 | 0.24666844 | 0.84313639 | -1.2931687 | -1.1296655 | -0.2902956 | 0.2592284 | -0.1259829 | 0.38714555 | 0.1110071 |
| PFZ2H10 | 1 | 3.0579 | 39.9977 | 39.9977 | CCLD+ | 6556 | LabradorRetriever | 186958 | 6y4m | 113.25 | 20.4 | 316.288928 | 1.55 | 2.43429579 | 3 | 2.96722201 | 3.5 | 2.48620805 | 0.7 | 0.96806367 | 1.45 | 2.0325981 | 3.8 | 9.25761798 | 1.25 | 1.49030481 | 4.3 | 19.3170572 | -0.3959487 | -0.2707956 | 0.65508899 | -0.9967649 | -0.7430652 | -0.4450719 | -0.2159069 | -0.1439979 | 0.06295132 | -0.0252252 |
| PFZ2F12 | 1 | 3.1690 | 45.0009 | 45.0009 | CCLD+ | 2330 | LabradorRetriever | 189713 | 5y11m | 120.5 | 20.5 | 319.255304 | 1.6 | 2.59635196 | 3.3 | 3.26083482 | 4.1 | 2.78929125 | 0.75 | 0.97416063 | 1.8 | 3.0712503 | 3.9 | 9.66728896 | 1.15 | 1.28388977 | 4.4 | 20.2399266 | 0.61687677 | 0.92724213 | -0.2895822 | -0.1635699 | 0.12244487 | -0.9139408 | 0.37090111 | 0.01497111 | 0.0379736 | -0.0336917 |
| PFZ2B12 | 2 | 2.9931 | 32.0009 | 37.2715 | CCLD+ | 770 | LabradorRetriever | 173463 | 3y3m | 114.5 | 21.2 | 340.388719 | 1.7 | 2.9363736 | 2.4 | 2.37908046 | 3.1 | 2.27624996 | 0.75 | 0.97416063 | 1.9 | 3.40518431 | 3.5 | 8.07163342 | 0.95 | 0.9123675 | 4.3 | 19.3170572 | -0.6232892 | 0.06824598 | 0.64024191 | 0.2411392 | 1.10325806 | -0.0519857 | -0.2816997 | -0.0432395 | -0.8709555 | 0.32521868 |
| PFZ29D05 | 2 | 3.0835 | 33.0306 | 38.4707 | CCLD+ | 9267 | LabradorRetriever | 222611 | 3y0m | 116.85 | 19.35 | 285.93938 | 1.55 | 2.43429579 | 2.85 | 2.82030717 | 3.35 | 2.4082833 | 0.9 | 0.99045801 | 1.6 | 2.45281643 | 3.6 | 8.45972545 | 1.1 | 1.18579638 | 4.15 | 17.9737061 | -0.6430172 | -0.0848285 | -0.4576693 | -0.3251257 | 0.14700951 | -0.2716415 | -0.0379009 | -0.0511946 | -0.0433399 | 0.10348384 |
| PFZ29D04 | 2 | 3.0755 | 34.9999 | 40.7644 | CCLD- | 3971 | GoldenRetriever | 166266 | 3y6m | 114.1 | 21.6 | 352.754214 | 1.6 | 2.59635196 | 3.65 | 3.60304707 | 3.05 | 2.24949993 | 1.25 | 1.02051363 | 1.6 | 2.45281643 | 4.25 | 11.1564069 | 1.3 | 1.59856652 | 4.8 | 24.1501433 | 0.9723138 | -1.2466264 | -0.5884062 | -1.1242417 | -0.365461 | 0.8939409 | 0.15845038 | -0.0860219 | 0.1214457 | 0.16045963 |
| PFZ29B05 | 1 | 3.0625 | 40.2000 | 40.2000 | CCLD- | 3789 | LabradorRetriever | 177578 | na | 120.2 | 21.65 | 354.314669 | 1.6 | 2.59635196 | 3.5 | 3.4564268 | 3.65 | 2.56322608 | 1.3 | 1.02416244 | 1.7 | 2.75376573 | 3.95 | 9.8747784 | 1.25 | 1.49030481 | 4.1 | 17.5368355 | 0.88037679 | -0.3479309 | -1.744038 | -0.4427093 | -0.109161 | -0.1369018 | 0.06919215 | 0.36695134 | -0.0967816 | -0.2843649 |
| PFZ26A03 | 1 | 3.0789 | 40.9140 | 40.9140 | CCLD+ | 8873 | LabradorRetriever | 218949 | 4y2m | 113.8 | 21.9 | 362.166116 | 1.65 | 2.7637096 | 3.2 | 3.1629948 | 4.55 | 3.00866813 | 0.85 | 0.9853196 | 1.8 | 3.0712503 | 3.65 | 8.65649744 | 1.1 | 1.18579638 | 4.6 | 22.1512581 | 0.71523784 | -0.5453369 | 0.43451049 | 0.38101073 | -0.0071407 | -1.1841409 | 0.17430589 | -0.2373009 | -0.2300074 | 0.41927402 |
| PFZ22H06 | 1 | 2.9348 | 34.9266 | 34.9266 | CCLD- | 8325 | GermanShepherd | 215195 | 1y9m | 110.8 | 20.7 | 325.227596 | 1.6 | 2.59635196 | 3.1 | 3.0651242 | 4 | 2.73966596 | 1.5 | 1.03758648 | 1.6 | 2.45281643 | 3.9 | 9.66728896 | 1.1 | 1.18579638 | 4.3 | 19.3170572 | 0.28420141 | -2.3600437 | -0.7439868 | 0.14635474 | 0.09573768 | -0.3111291 | 0.06643326 | -0.4270647 | 0.02908168 | 0.00377046 |
| PFZ22G08 | 2 | 2.6702 | 21.9539 | 25.5697 | CCLD+ | 7302 | SiberianHusky | 164240 | 9y3m | 119.5 | 16.4 | 208.51481 | 1.2 | 1.44789787 | 2.9 | 2.86928718 | 2.6 | 2.00302156 | 0.8 | 0.97989872 | 1.1 | 1.19955078 | 3.1 | 6.59325608 | 1 | 1 | 3.4 | 11.9922922 | -2.7686501 | 0.63315078 | -1.1482312 | -0.3348422 | -0.6636693 | 0.18457891 | 0.06611124 | 0.04945017 | 0.36175137 | -0.0799555 |
| PFZ22G06 | 1 | 2.5023 | 20.6384 | 20.6384 | CCLD+ | 8336 | StaffordshireBullTerrier | 210090 | 1y9m | 118.1 | 21.5 | 349.643147 | 1.6 | 2.59635196 | 3.65 | 3.60304707 | 3.6 | 2.53765114 | 0.9 | 0.99045801 | 1.75 | 2.9104466 | 3.6 | 8.45972545 | 1 | 1 | 4.15 | 17.9737061 | 0.06587231 | 0.25352104 | -0.4637202 | 0.70797061 | 0.02969573 | -0.0988597 | 0.16436625 | 0.17983738 | -0.1099869 | 0.04926587 |
| PFZ22A07 | 2 | n/a | n/a | n/a | CCLD+ | 3903 | Boerboel | 170916 | 3y2m | 124.1 | 23.45 | 412.669807 | 2.1 | 4.50925887 | 3.35 | 3.30974366 | 3.9 | 2.68970078 | 0.7 | 0.96806367 | 2.05 | 3.93675258 | 4.55 | 12.4998557 | 1.25 | 1.49030481 | 5 | 26.2366939 | 2.80955791 | 1.89692368 | 0.00256031 | -0.5908337 | 1.08537281 | -0.2889525 | -0.7082544 | -0.21647 | -0.1578612 | -0.074681 |
| PFZ21D08 | 2 | 3.3568 | 46.7200 | 54.4148 | CCLD+ | 6298 | SaintBernard | 208228 | 2y0m | 114.8 | 23.85 | 426.211657 | 1.9 | 3.68018635 | 4.5 | 4.43282298 | 4 | 2.73966596 | 0.9 | 0.99045801 | 1.8 | 3.0712503 | 4.05 | 10.2950278 | 1.15 | 1.28388977 | 4.7 | 23.1397494 | 1.92408016 | -0.2867952 | 0.40532003 | 0.63322806 | -0.4006008 | 0.14845636 | -0.3629196 | 0.1083573 | 0.27176055 | 0.12347958 |
| PFZ20D03 | 2 | 3.5481 | 56.0999 | 65.3395 | CCLD+ | 5376 | GreatDane | 172203 | 4y11m | 119.5 | 33.8 | 829.281507 | 2.2 | 4.95584873 | 7.5 | 7.35039454 | 4.6 | 3.03266852 | 0.9 | 0.99045801 | 2.2 | 4.50489733 | 5.4 | 16.630312 | 1.3 | 1.59856652 | 6.1 | 39.2843494 | 7.68068032 | 0.95010392 | 0.97116482 | 1.52767603 | -1.6545828 | 1.97430323 | 0.18957299 | -0.2193977 | -0.3574517 | 0.08422631 |
| PFZ20B01 | 2 | 3.5422 | 55.7894 | 64.9779 | CCLD+ | 2254 | GreatDane | 176128 | 2y7m | 124.7 | 30.65 | 688.011637 | 2 | 4.0840485 | 6 | 5.89345183 | 5.2 | 3.31538853 | 1 | 1 | 2.15 | 4.31146654 | 5.1 | 15.1188752 | 1.3 | 1.59856652 | 5.95 | 37.3481798 | 6.45941821 | 1.35750854 | -0.4191569 | 0.82033829 | -0.8992017 | 0.39413195 | 0.71853299 | -0.5463966 | -0.3717616 | 0.31851948 |
| PFZ1H03 | 2 | 2.8204 | 26.2993 | 30.6308 | CCLD+ | 771 | LabradorRetriever | 143885 | 6y6m | 115.7 | 21 | 334.284803 | 1.65 | 2.7637096 | 3.1 | 3.0651242 | 2.9 | 2.16851956 | 0.65 | 0.96155718 | 1.4 | 1.90089628 | 3.4 | 7.69086795 | 1.2 | 1.38540282 | 4.2 | 18.4160319 | -0.8259257 | 0.45520134 | 0.41778981 | -0.7594231 | -0.7341595 | 0.13147523 | -0.7719424 | 0.13230528 | -0.0682767 | 0.27417699 |
| PFZ1F09 | 2 | 3.0016 | 32.3003 | 37.6201 | CCLD+ | 1675 | LabradorRetriever | 186083 | 2y8m | 117.6 | 19.7 | 295.893909 | 1.7 | 2.9363736 | 3.3 | 3.26083482 | 3.1 | 2.27624996 | 0.6 | 0.95457875 | 1.9 | 3.40518431 | 4.1 | 10.5077732 | 1.1 | 1.18579638 | 4.8 | 24.1501433 | 0.37365871 | 1.10850504 | 0.93488852 | -0.4721209 | 0.70491818 | 0.28199681 | 0.28164166 | -0.0309348 | 0.09238126 | 0.2310442 |
| PFZ1F03 | 1 | 2.5858 | 22.9971 | 22.9971 | CCLD+ | 2972 | LabradorRetriever | 196786 | 9y9m | 118.25 | 18.8 | 270.624639 | 1.5 | 2.27753603 | 2.7 | 2.67331495 | 3.75 | 2.61409118 | 0.7 | 0.96806367 | 1.7 | 2.75376573 | 3.2 | 6.95160383 | 1.05 | 1.09115505 | 4 | 16.6794522 | -0.979452 | 0.66459895 | -0.0979139 | 0.0379377 | 0.22277975 | -1.0833325 | 0.1024109 | 0.19787759 | -0.174457 | 0.27581409 |
| PFZ1E08 | 2 | 2.8682 | 27.8007 | 32.3794 | CCLD+ | 100 | LabradorRetriever | 168670 | na | 121.6 | 20.6 | 322.234862 | 1.5 | 2.27753603 | 4.1 | 4.04255575 | 3.3 | 2.38209805 | 0.8 | 0.97989872 | 1.73 | 2.84727891 | 3.5 | 8.07163342 | 1.1 | 1.18579638 | 4.5 | 21.1846554 | 0.20560274 | 1.15775151 | -0.6841405 | 0.18103311 | -0.3395819 | 0.21081505 | 0.5398502 | 0.49821018 | 0.2205022 | 0.50060007 |
| PFZ19F06 | 2 | 2.7432 | 23.9996 | 27.9523 | CCLD+ | 2173 | EnglishSpringerSpaniel | 188534 | 11y3m | 120.5 | 17.2 | 228.361983 | 1.45 | 2.12606748 | 3.3 | 3.26083482 | 2.85 | 2.14127385 | 0.65 | 0.96155718 | 1.6 | 2.45281643 | 3.3 | 7.31750011 | 0.9 | 0.82829608 | 3.65 | 13.8501512 | -1.7728486 | 1.40654071 | -0.3359446 | 0.41873266 | 0.198627 | 0.10609083 | 0.20526258 | 0.3698688 | 0.31642997 | -0.0928917 |
| PFZ17D11 | 2 | 2.7450 | 24.0494 | 28.0104 | CCLD- | 8249 | BorderCollie | 218026 | 7y3m | 107.9 | 17.2 | 228.361983 | 1.1 | 1.21346471 | 2.8 | 2.77131856 | 3 | 2.2226299 | 1.3 | 1.02416244 | 1.2 | 1.41630569 | 3 | 6.24253709 | 1 | 1 | 3.1 | 9.94178206 | -2.7716947 | -2.5872444 | -0.6812148 | 0.13010751 | -0.6538788 | 0.02235635 | 0.2000144 | 0.23536074 | 0.04738447 | -0.1539956 |
| PFZ17B06 | 1 | 2.0153 | 10.1015 | 10.1015 | CCLD- | 3299 | Pug | 194883 | 4y4m | 114.3 | 13.8 | 149.978559 | 1.2 | 1.44789787 | 2.2 | 2.18272214 | 2.4 | 1.88979011 | 0.8 | 0.97989872 | 1.3 | 1.65012886 | 2.5 | 4.60644611 | 0.7 | 0.5284882 | 2.9 | 8.68296271 | -4.1629571 | -0.2045784 | -0.4294598 | 0.55945807 | 0.38444465 | 0.08244399 | -0.0386294 | 0.14424297 | 0.04495657 | 0.10843368 |
| PFZ16H02 | 2 | 3.4654 | 51.8999 | 60.4478 | CCLD+ | 7452 | GreatPyrenees | 214153 | 4y11m | 115.5 | 21.1 | 337.330187 | 1.45 | 2.12606748 | 3.45 | 3.40753954 | 3.35 | 2.4082833 | 0.95 | 0.99534319 | 1.6 | 2.45281643 | 3.5 | 8.07163342 | 1.1 | 1.18579638 | 4.1 | 17.5368355 | -0.4546939 | -0.405222 | -0.4128534 | 0.07410678 | -0.4448438 | 0.01612394 | 0.21110697 | 0.2457483 | -0.1748641 | 0.11594531 |
| PFZ16F11 | 1 | 2.9302 | 34.7459 | 34.7459 | CCLD+ | 6613 | Dalmatian | 177268 | 5y2m | 115.35 | 20.85 | 329.741392 | 1.55 | 2.43429579 | 3.2 | 3.1629948 | 3.25 | 2.35580426 | 0.9 | 0.99045801 | 1.6 | 2.45281643 | 3.55 | 8.26476795 | 0.85 | 0.74782682 | 4 | 16.6794522 | -0.9556178 | -0.1993373 | -0.207218 | 0.88648272 | 0.15422774 | 0.20536523 | 0.02213248 | -0.3137378 | -0.1973682 | -0.020302 |
| PFZ16B08 | 1 | 1.9718 | 9.4000 | 9.4000 | CCLD+ | 4366 | JackRussellTerrier | 195137 | 8y9m | 111.6 | 10 | 81.0961058 | 0.95 | 0.9011123 | 1.8 | 1.78945087 | 2.25 | 1.80317007 | 0.55 | 0.94705021 | 1.2 | 1.41630569 | 2.8 | 5.56432436 | 0.75 | 0.59787382 | 2.95 | 8.98956532 | -4.9842057 | 0.0385824 | 0.77692602 | -0.2188035 | 0.25810103 | -0.1024386 | 0.36388344 | -0.1007258 | 0.26292717 | -0.1270871 |
| PFZ16A09 | 2 | 1.5868 | 3.9406 | 4.5896 | CCLD- | 7368 | Pomeranian | 196563 | 6y6m | 108.2 | 9 | 66.320677 | 0.6 | 0.35452514 | 1.05 | 1.04948783 | 0.9 | 0.92626297 | 0.85 | 0.9853196 | 0.7 | 0.50616505 | 1.7 | 2.42191196 | 0.5 | 0.2895732 | 1.9 | 3.68018635 | -7.4288696 | -1.3432691 | -0.2703158 | -0.1324598 | 0.39288272 | 1.47903555 | -0.2547439 | 0.04100898 | -0.4138468 | 0.22791528 |
| PFZ15H08 | 1 | 3.1902 | 42.5999 | 42.5999 | CCLD+ | 7381 | Rottweiler | 208061 | 4y9m | 110.15 | 21.4 | 346.545204 | 1.5 | 2.27753603 | 3 | 2.96722201 | 4 | 2.73966596 | 0.85 | 0.9853196 | 1.7 | 2.75376573 | 3.75 | 9.0554518 | 1 | 1 | 4.2 | 18.4160319 | -0.3626261 | -1.1582205 | 0.79566247 | 0.48295452 | -0.0586677 | -0.6506424 | 0.3110445 | -0.357123 | -0.366202 | -0.0759064 |
| PFZ15H07 | 2 | 3.4368 | 50.4999 | 58.8172 | CCLD+ | 2704 | CaneCorso | 190537 | 1y9m | 120.6 | 23.95 | 429.629638 | 1.8 | 3.29763957 | 4.55 | 4.48158133 | 3.95 | 2.71472654 | 0.8 | 0.97989872 | 2 | 3.75548699 | 4.3 | 11.3760611 | 1.2 | 1.38540282 | 4.8 | 24.1501433 | 2.44471489 | 1.01178587 | -0.0030011 | 0.26361242 | -0.0678964 | 0.10660717 | 0.33674671 | 0.38941964 | 0.02650329 | -0.0603311 |
| PFZ15G09 | 2 | 3.0835 | 35.2999 | 41.1138 | CCLD+ | 2063 | Rottweiler | 187662 | 5y8m | 112.5 | 19.9 | 301.654996 | 1.7 | 2.9363736 | 3.1 | 3.0651242 | 2.7 | 2.05873978 | 0.6 | 0.95457875 | 1.9 | 3.40518431 | 4.3 | 11.3760611 | 1.2 | 1.38540282 | 5 | 26.2366939 | 0.38335884 | 0.21230979 | 1.76027554 | -1.2144634 | 0.66797737 | 0.77873259 | 0.20353534 | -0.0444268 | -0.0575078 | 0.27239298 |
| PFZ15D06 | 2 | 2.9129 | 29.2569 | 34.0756 | CCLD+ | 6666 | GoldenRetriever | 209438 | 7y8m | 113.05 | 17.9 | 246.431622 | 1.45 | 2.12606748 | 2.75 | 2.72232121 | 3.1 | 2.27624996 | 0.9 | 0.99045801 | 1.55 | 2.30857087 | 3.15 | 6.77148145 | 1 | 1 | 3.55 | 13.0907207 | -1.8540728 | -0.749772 | -0.1296854 | 0.13381529 | 0.12991132 | -0.2317692 | -0.1164015 | 0.33708708 | -0.0567181 | 0.00770926 |
| PFZ15D04 | 2 | 3.4875 | 53.0000 | 61.7291 | CCLD+ | 5440 | CaneCorso | 175020 | 0y11m | 108.5 | 23.6 | 417.723601 | 2 | 4.0840485 | 3.8 | 3.74960708 | 4.4 | 2.93623016 | 1 | 1 | 2 | 3.75548699 | 4.2 | 10.9384697 | 1.3 | 1.59856652 | 4.8 | 24.1501433 | 2.50140694 | -1.7634905 | 1.26258663 | -0.0913516 | 0.23199523 | -0.4865998 | -0.5945939 | 0.32146727 | -0.0569437 | 0.11504662 |
| PFZ15B11 | 1 | 2.9394 | 35.1089 | 35.1089 | CCLD+ | 6989 | Weimaraner | 210710 | 7y3m | 113.1 | 20.5 | 319.255304 | 1.6 | 2.59635196 | 3.6 | 3.55418047 | 3.8 | 2.63938458 | 0.9 | 0.99045801 | 1.6 | 2.45281643 | 3.8 | 9.25761798 | 1.2 | 1.38540282 | 4.1 | 17.5368355 | 0.14215259 | -0.8009951 | 0.16199919 | -0.203511 | -0.5500862 | -0.4115672 | -0.0446863 | 0.24965535 | 0.28382101 | -0.1917366 |
| PFZ14H03 | 2 | 1.7257 | 5.1982 | 6.0543 | CCLD- | 5996 | LhasaApso | 203982 | 5y10m | 114.1 | 9 | 66.320677 | 0.6 | 0.35452514 | 1.45 | 1.44462233 | 1.8 | 1.53314458 | 0.6 | 0.95457875 | 0.8 | 0.65312873 | 1.8 | 2.66403095 | 0.6 | 0.40117553 | 2.05 | 4.29398317 | -6.5680011 | 0.30083497 | -0.2533017 | 0.05588805 | -0.0728779 | 0.05934545 | 0.09635465 | 0.03161779 | -0.0098342 | 0.10247207 |
| PFZ14G12 | 2 | 2.8525 | 27.3017 | 31.7983 | CCLD+ | 1188 | LabradorRetriever | 183265 | 0y6m | 121.35 | 21 | 334.284803 | 1.45 | 2.12606748 | 3.3 | 3.26083482 | 3.1 | 2.27624996 | 0.65 | 0.96155718 | 1.65 | 2.60121859 | 3.45 | 7.88033049 | 1 | 1 | 3.75 | 14.6313188 | -0.9815677 | 1.5206436 | -0.3862049 | 0.28431635 | -0.2060568 | -0.0638954 | 0.15426081 | 0.32792763 | -0.3475954 | -0.2226836 |
| PFZ14F05 | 1 | 2.8302 | 30.9860 | 30.9860 | CCLD- | 8108 | StaffordshireBullTerrier | 214097 | 1y2m | 110.05 | 20.7 | 325.227596 | 1.45 | 2.12606748 | 3.5 | 3.4564268 | 3.4 | 2.43436207 | 1 | 1 | 1.6 | 2.45281643 | 3.5 | 8.07163342 | 0.9 | 0.82829608 | 3.9 | 15.8438657 | -0.9967423 | -1.4181354 | 0.26435919 | 0.97026265 | -0.2220692 | 0.18333446 | 0.27059844 | -0.0173416 | -0.0904867 | -0.057899 |
| PFZ14E12 | 2 | 3.1045 | 36.1014 | 42.0473 | CCLD+ | 6085 | LabradorRetriever | 170197 | 11y0m | 118 | 19.6 | 293.033208 | 1.55 | 2.43429579 | 3.15 | 3.11406339 | 3.05 | 2.24949993 | 0.65 | 0.96155718 | 1.75 | 2.9104466 | 3.5 | 8.07163342 | 1.05 | 1.09115505 | 4.05 | 17.1054182 | -0.817898 | 0.93440403 | 0.25913961 | -0.0517574 | 0.22876137 | -0.0381955 | 0.07427531 | 0.4101841 | -0.1502687 | 0.05117215 |
| PFZ14B11 | 2 | 3.0621 | 34.5002 | 40.1824 | CCLD+ | 447 | CaneCorso | 178713 | 5y11m | 119.2 | 19.9 | 301.654996 | 1.7 | 2.9363736 | 3 | 2.96722201 | 4 | 2.73966596 | 0.7 | 0.96806367 | 1.7 | 2.75376573 | 3.7 | 8.85507561 | 0.8 | 0.67100367 | 4.2 | 18.4160319 | -0.4244574 | 0.97448395 | 0.00383083 | 1.06970153 | 0.62678973 | -0.7693566 | -0.0249809 | -0.7062554 | 0.10165904 | -0.0264408 |
| PFZ14B05 | 1 | 2.5433 | 23.9497 | 23.9497 | CCLD+ | 2487 | SoftCoatedWheatenTerrier | 190653 | 5y9m | 120.6 | 17.6 | 238.607276 | 1.4 | 1.97988476 | 2.9 | 2.86928718 | 3.1 | 2.27624996 | 0.8 | 0.97989872 | 1.2 | 1.41630569 | 3 | 6.24253709 | 0.9 | 0.82829608 | 3.4 | 11.9922922 | -2.3358563 | 0.842239 | -1.2048711 | 0.39219918 | -0.3991712 | -0.3046124 | -0.2375459 | -0.154386 | 0.29811929 | -0.0234137 |
| PFZ13G10 | 1 | 3.3300 | 52.9977 | 52.9977 | CCLD+ | 155 | DobermanPinscher | 176738 | 10y11m | 110.1 | 25.2 | 473.449467 | 1.7 | 2.9363736 | 3.6 | 3.55418047 | 4 | 2.73966596 | 1 | 1 | 1.8 | 3.0712503 | 4.5 | 12.271715 | 1.5 | 2.06467249 | 4.9 | 25.1824536 | 2.45492791 | -1.6238597 | 0.78870402 | -1.6054212 | -0.9124885 | -0.1825612 | -0.0423448 | 0.17421685 | -0.577174 | -0.1503285 |
| PFZ13F12 | 1 | 3.2214 | 47.4999 | 47.4999 | CCLD- | 1319 | Bullmastiff | 173119 | 2y2m | 114.3 | 24.3 | 441.694891 | 2 | 4.0840485 | 5.4 | 5.30969802 | 4.5 | 2.98459562 | 1.6 | 1.04369816 | 2.1 | 4.12208206 | 4.5 | 12.271715 | 1.3 | 1.59856652 | 5.1 | 27.3128775 | 4.17881903 | -1.656418 | -0.5271869 | 0.60056435 | 0.08284425 | 0.42504216 | 0.16630355 | 0.67993343 | 0.61120233 | 0.19474179 |
| PFZ13E10 | 1 | 3.2193 | 47.4004 | 47.4004 | CCLD+ | 1954 | DobermanPinscher | 185793 | 4y2m | 118 | 24.9 | 462.748003 | 1.7 | 2.9363736 | 4.3 | 4.23773476 | 4.1 | 2.78929125 | 0.9 | 0.99045801 | 1.8 | 3.0712503 | 4.4 | 11.8204936 | 1.2 | 1.38540282 | 5.2 | 28.4110175 | 2.41580842 | 0.23893624 | 0.01424386 | 0.01775376 | -0.5893654 | 0.16964032 | 0.50147497 | -0.5049845 | -0.1078274 | 0.29061709 |
| PFZ13B12 | 1 | 2.0666 | 10.9769 | 10.9769 | CCLD+ | 6279 | PembrokeWelshCorgi | 208101 | 1y7m | 103.9 | 8.1 | 54.2372799 | 0.8 | 0.63572995 | 1.5 | 1.49393034 | 1.9 | 1.59460774 | 0.7 | 0.96806367 | 1.3 | 1.65012886 | 2.3 | 4.00866997 | 0.6 | 0.40117553 | 2.6 | 6.9565818 | -5.9502345 | -1.7786477 | 1.26265564 | 0.20363214 | 0.69509707 | 0.2964783 | 0.40087467 | 0.22575443 | -0.0537118 | 0.12111265 |
| PFZ13B11 | 2 | 3.6909 | 63.8999 | 74.4242 | CCLD+ | 505 | DoguedeBordeaux | 166187 | 3y2m | 114.9 | 25.5 | 484.267367 | 1.9 | 3.68018635 | 5 | 4.92017222 | 4.8 | 3.12796856 | 0.6 | 0.95457875 | 2.2 | 4.50489733 | 4.7 | 13.1943235 | 1.2 | 1.38540282 | 5 | 26.2366939 | 3.56310929 | 0.61755497 | 1.89782214 | 0.75602348 | -0.1239274 | -0.4132425 | 0.57439883 | 0.31703427 | 0.01257705 | -0.3948657 |
| PFZ13A09 | 1 | 3.4570 | 59.9649 | 59.9649 | CCLD+ | 1247 | SaintBernard | 177351 | 2y4m | 117.45 | 29.1 | 623.120036 | 2.1 | 4.50925887 | 5.8 | 5.69893513 | 5.1 | 3.26891417 | 0.95 | 0.99534319 | 2.15 | 4.31146654 | 5 | 14.6279342 | 1.3 | 1.59856652 | 5.4 | 30.6732174 | 5.61392921 | 0.13660093 | 0.64619068 | 0.92371542 | -0.6614171 | 0.1882971 | 0.05793972 | -0.000867 | -0.0561852 | -0.2580426 |
| PFZ12A06 | 2 | 1.8086 | 6.0691 | 7.0686 | CCLD+ | 3495 | BichonFrise | 195237 | 6y11m | 122.9 | 8.1 | 54.2372799 | 0.7 | 0.48478483 | 1.4 | 1.3952973 | 1.4 | 1.27712993 | 0.5 | 0.93887175 | 0.9 | 0.81780347 | 2.2 | 3.72236202 | 0.7 | 0.5284882 | 2.3 | 5.42384796 | -6.2574544 | 2.30324249 | -1.0265497 | -0.6492365 | 0.25683649 | 0.41546383 | 0.12044441 | 0.10851998 | 0.02489399 | -0.0358783 |
| PFZ11F06 | 1 | 3.0625 | 40.1999 | 40.1999 | CCLD+ | 1223 | Bulldog,NOS | 183612 | 3y2m | 118.9 | 26.8 | 532.487383 | 2 | 4.0840485 | 4.5 | 4.43282298 | 4.4 | 2.93623016 | 0.9 | 0.99045801 | 2.1 | 4.12208206 | 4.6 | 12.7296747 | 1.1 | 1.18579638 | 4.9 | 25.1824536 | 3.42957154 | 0.52848575 | 0.2372536 | 1.05965689 | 0.31328185 | 0.09958568 | -0.0911748 | -0.1807217 | -0.4531788 | -0.3204338 |
| PFZ11A07 | 1 | 2.9618 | 35.9999 | 35.9999 | CCLD+ | 5443 | ChesapeakeBayRetriever | 163152 | 10y9m | 113.3 | 21.3 | 343.460393 | 1.7 | 2.9363736 | 3.8 | 3.74960708 | 3.5 | 2.48620805 | 0.7 | 0.96806367 | 1.8 | 3.0712503 | 3.8 | 9.25761798 | 1 | 1 | 4.4 | 20.2399266 | 0.17481685 | 0.00822629 | 1.03642318 | 0.63774853 | 0.05560203 | 0.12077301 | 0.04745345 | 0.09439493 | 0.0978953 | 0.0748223 |
| PFZ10E11 | 1 | 3.1034 | 41.9981 | 41.9981 | CCLD+ | 80 | AiredaleTerrier | 173973 | na | 123.3 | 23.1 | 400.991595 | 1.75 | 3.11434871 | 3.85 | 3.79844745 | 3.55 | 2.51197905 | 0.7 | 0.96806367 | 1.7 | 2.75376573 | 4.2 | 10.9384697 | 1.2 | 1.38540282 | 4.7 | 23.1397494 | 1.36641002 | 1.71363909 | -0.3934301 | -0.3869068 | -0.3581221 | 0.10082374 | -0.0878217 | -0.1958244 | 0.03547724 | -0.0623679 |
| PFZ10C11 | 2 | 3.0940 | 35.6999 | 41.5797 | CCLD+ | 125 | ChesapeakeBayRetriever | 162786 | 4y11m | 111.3 | 20.3 | 313.335741 | 1.4 | 1.97988476 | 3.7 | 3.65190698 | 2.9 | 2.16851956 | 0.5 | 0.93887175 | 1.7 | 2.75376573 | 3.8 | 9.25761798 | 0.9 | 0.82829608 | 4.1 | 17.5368355 | -1.252935 | 0.39970626 | 1.91280649 | 0.54247805 | -0.310894 | 0.54224431 | 0.59725128 | 0.08099792 | -0.0210227 | -0.2686851 |
| n/a | 1 | 1.7632 | 6.5000 | 6.5000 | CCLD- | na | Affenpinscher | 240501 | 4y11m | 110.9 | 10.3 | 85.8037486 | 0.8 | 0.63572995 | 1.2 | 1.19781413 | 1.3 | 1.21014335 | 0.4 | 0.91999922 | 0.7 | 0.50616505 | 1.9 | 2.91529329 | 0.5 | 0.2895732 | 2.1 | 4.50925887 | -7.2204071 | 0.68849762 | 1.20346877 | -0.0769 | -0.0807857 | 0.60392537 | -0.3719759 | -0.1950583 | -0.2322576 | 0.0167501 |
| n/a | 2 | n/a | n/a | n/a | CCLD- | na | AustralianSheepDog | 166462 | 12y1m | 109.8 | 14.8 | 171.407419 | 1.4 | 1.97988476 | 2.3 | 2.28092265 | 2.3 | 1.83221374 | 0.9 | 0.99045801 | 1.3 | 1.65012886 | 4 | 10.0840271 | 1 | 1 | 3.2 | 10.6036273 | -2.7306906 | -1.3212481 | 0.2453068 | -0.8081214 | 0.37671112 | 0.71870943 | -0.1587429 | -0.2814724 | 0.37742146 | -1.0143182 |
| n/a | 2 | 2.5058 | 17.8000 | 20.7317 | CCLD- | na | Beagle | 215106 | 6y10m | 115.1 | 12.6 | 126.068728 | 1.1 | 1.21346471 | 2 | 1.98618499 | 2.2 | 1.77394965 | 0.8 | 0.97989872 | 1.1 | 1.19955078 | 2.7 | 5.2370099 | 0.7 | 0.5284882 | 2.9 | 8.68296271 | -4.5567283 | -0.0865585 | -0.6600443 | 0.1921571 | 0.27240457 | 0.27435998 | 0.02509764 | -0.2082509 | 0.18012759 | -0.032158 |
| n/a | 2 | 2.8040 | 25.8000 | 30.0493 | CCLD- | na | Boxer | 216248 | 3y11m | 116.3 | 19.1 | 278.928376 | 1.5 | 2.27753603 | 3.2 | 3.1629948 | 3.6 | 2.53765114 | 1.1 | 1.00871095 | 1.5 | 2.16849394 | 3.4 | 7.69086795 | 1 | 1 | 4 | 16.6794522 | -0.8167549 | -0.6258084 | -0.9918344 | 0.40565018 | -0.0834534 | -0.3102105 | 0.08279605 | -0.1231474 | 0.21725468 | 0.17001384 |
| n/a | 2 | 3.1531 | 38.0000 | 44.2586 | CCLD- | na | ChesapeakeBayRetriever | 238048 | 5y10m | 114.2 | 19.9 | 301.654996 | 1.7 | 2.9363736 | 3.3 | 3.26083482 | 3.5 | 2.48620805 | 0.9 | 0.99045801 | 1.7 | 2.75376573 | 4.2 | 10.9384697 | 1.2 | 1.38540282 | 4.6 | 22.1512581 | 0.59146398 | -0.5273969 | 0.26141368 | -0.6840434 | 0.17858714 | 0.03002274 | 0.01244002 | -0.2046551 | 0.31970596 | -0.0520514 |
| n/a | 1 | 1.3005 | 2.3800 | 2.3800 | CCLD- | na | Chihuahua | 224485 | 6y5m | 124.3 | 6.2 | 32.5593507 | 0.5 | 0.24485507 | 0.9 | 0.90094874 | 0.9 | 0.92626297 | 0.4 | 0.91999922 | 0.5 | 0.26627705 | 1.5 | 1.96582172 | 0.5 | 0.2895732 | 1.6 | 2.59635196 | -7.7951316 | 3.07186929 | -0.9971041 | -0.5716443 | 0.22624237 | 0.70498599 | -0.2316324 | -0.0156349 | -0.2100895 | 0.08408071 |
| n/a | 2 | 1.5334 | 3.5200 | 4.0997 | CCLD- | na | Chihuahua | 239769 | 8y5m | 109.1 | 7.9 | 51.7094737 | 0.6 | 0.35452514 | 1.2 | 1.19781413 | 1.4 | 1.27712993 | 0.4 | 0.91999922 | 0.6 | 0.3771297 | 1.6 | 2.18911215 | 0.4 | 0.19430468 | 1.7 | 2.9363736 | -7.7399478 | 0.33366468 | 1.34705198 | 0.18139317 | -0.1714921 | 0.37200038 | -0.2043537 | -0.1370259 | -0.0725424 | 0.02837445 |
| n/a | 2 | 1.8423 | 6.4500 | 7.5123 | CCLD- | na | Dachshund | 183201 | 6y1m | 101 | 6.2 | 32.5593507 | 0.8 | 0.63572995 | 1.2 | 1.19781413 | 1.3 | 1.21014335 | 0.9 | 0.99045801 | 1 | 1 | 2.3 | 4.00866997 | 0.7 | 0.5284882 | 2.5 | 6.42418764 | -6.5545713 | -2.8317865 | 0.90377207 | -0.4874579 | 0.58804608 | 1.09972145 | -0.0424432 | 0.13360055 | -0.0008996 | 0.15968158 |
| n/a | 2 | 3.2711 | 42.9000 | 49.9656 | CCLD- | na | GermanShepherd | 206841 | 6y0m | 121 | 23.4 | 410.991719 | 1.8 | 3.29763957 | 4.3 | 4.23773476 | 4.7 | 3.08045694 | 1.5 | 1.03758648 | 1.8 | 3.0712503 | 4.4 | 11.8204936 | 1.2 | 1.38540282 | 4.9 | 25.1824536 | 2.87068076 | -0.4788944 | -1.7860021 | 0.35808428 | -0.0678296 | -0.3410493 | 0.32222175 | -0.4060074 | 0.37093639 | 0.04207083 |
| PFZ24E03 | 1 | 2.7711 | 28.8999 | 28.8999 | CCLD- | 2911 | GoldenRetriever | 189603 | 12y9m | 105.8 | 19.6 | 293.033208 | 1.4 | 1.97988476 | 3.1 | 3.0651242 | 3 | 2.2226299 | 1.3 | 1.02416244 | 1.5 | 2.16849394 | 3.4 | 7.69086795 | 1.2 | 1.38540282 | 3.8 | 15.0300595 | -1.1492879 | -2.9090909 | 0.05268924 | -0.4976176 | -0.4951132 | 0.3313529 | -0.0687118 | 0.57138189 | -0.1386328 | 0.0421145 |
| n/a | 2 | 2.9587 | 30.8000 | 35.8728 | CCLD- | na | GoldenRetriever | 212024 | 3y3m | 114 | 22.1 | 368.506244 | 1.5 | 2.27753603 | 3.5 | 3.4564268 | 3.5 | 2.48620805 | 1.3 | 1.02416244 | 1.6 | 2.45281643 | 3.4 | 7.69086795 | 1.1 | 1.18579638 | 3.8 | 15.0300595 | -0.2575395 | -1.3944758 | -1.0329652 | 0.42919394 | -0.4436584 | 0.01450643 | -0.0422098 | 0.41881912 | -0.3513706 | -0.0301887 |
| n/a | 1 | 2.7447 | 28.0000 | 28.0000 | CCLD- | na | GoldenRetriever | 217185 | 4y2m | 114 | 20 | 304.55537 | 1.5 | 2.27753603 | 2.8 | 2.77131856 | 3.7 | 2.58870554 | 1.1 | 1.00871095 | 1.5 | 2.16849394 | 3.6 | 8.45972545 | 1.2 | 1.38540282 | 3.9 | 15.8438657 | -0.5162559 | -1.1725595 | -0.6838104 | -0.536364 | -0.3184744 | -0.6430705 | -0.1395367 | 0.04560578 | -0.1123535 | -0.1293991 |
| n/a | 2 | 2.7010 | 22.8000 | 26.5552 | CCLD- | na | Husky | 234453 | 7y7m | 110.5 | 19.1 | 278.928376 | 1.3 | 1.70335435 | 2.5 | 2.47719736 | 2.7 | 2.05873978 | 1 | 1 | 1.2 | 1.41630569 | 3.4 | 7.69086795 | 1 | 1 | 3.7 | 14.2380168 | -2.3500625 | -1.4442335 | -0.1905415 | -0.3010391 | -0.4555524 | 0.40572858 | -0.1334225 | -0.4023524 | -0.2391 | -0.0874713 |
| n/a | 2 | 3.4855 | 52.9000 | 61.6126 | CCLD- | na | IrishWolfhound | 224301 | 8y10m | 119.4 | 27.5 | 559.353181 | 1.8 | 3.29763957 | 4.2 | 4.14015688 | 4.7 | 3.08045694 | 1.6 | 1.04369816 | 1.8 | 3.0712503 | 4.4 | 11.8204936 | 1.3 | 1.59856652 | 4.7 | 23.1397494 | 3.35656725 | -0.9438826 | -1.7549861 | 0.23338004 | -0.7219786 | -0.2723825 | -0.0926672 | -0.2186236 | -0.686717 | -0.2630664 |
| n/a | 1 | 3.2357 | 48.2000 | 48.2000 | CCLD- | na | LabradorRetriever | 236787 | 10y0m | 113.2 | 23 | 397.684294 | 1.7 | 2.9363736 | 3.6 | 3.55418047 | 4 | 2.73966596 | 1.3 | 1.02416244 | 1.9 | 3.40518431 | 4.6 | 12.7296747 | 1.2 | 1.38540282 | 4.8 | 24.1501433 | 2.00587696 | -1.5048363 | -0.2315644 | -0.2214226 | 0.36325097 | 0.13302603 | 0.48289513 | -0.3662253 | -0.1872537 | -0.2802421 |
| n/a | 1 | 2.8580 | 32.0000 | 32.0000 | CCLD- | na | LabradorRetriever | 206639 | 12y3m | 118.8 | 22.9 | 394.390037 | 1.5 | 2.27753603 | 3.1 | 3.0651242 | 3.9 | 2.68970078 | 0.8 | 0.97989872 | 1.6 | 2.45281643 | 3.7 | 8.85507561 | 1.1 | 1.18579638 | 4.2 | 18.4160319 | 0.03040674 | 0.48600706 | -0.4222205 | 0.06788534 | -0.507721 | -0.6751006 | 0.11749374 | -0.2934676 | -0.5450308 | -0.0718168 |
| n/a | 1 | 3.2007 | 46.5000 | 46.5000 | CCLD- | na | LabradorRetriever | 234690 | 5y0m | 117.9 | 20.3 | 313.335741 | 1.7 | 2.9363736 | 3.8 | 3.74960708 | 3.6 | 2.53765114 | 1.4 | 1.03109256 | 1.8 | 3.0712503 | 4 | 10.0840271 | 1.1 | 1.18579638 | 4.2 | 18.4160319 | 0.87022399 | -0.7959143 | -1.4230555 | 0.32942121 | 0.49764924 | 0.2307282 | 0.15441861 | 0.27606485 | 0.31820922 | -0.1861014 |
| n/a | 2 | 3.0432 | 33.8000 | 39.3669 | CCLD- | na | LabradorRetriever | 220738 | 6y10m | 116.3 | 18 | 249.066439 | 1.5 | 2.27753603 | 3.7 | 3.65190698 | 3.1 | 2.27624996 | 1.1 | 1.00871095 | 1.5 | 2.16849394 | 3.6 | 8.45972545 | 1.2 | 1.38540282 | 4.1 | 17.5368355 | -0.4507922 | -0.6194602 | -0.9515326 | -0.5255801 | -0.4003724 | 0.30215219 | 0.12104844 | 0.50544309 | 0.64663203 | 0.11293407 |
| 435 | 1 | 3.2336 | 48.0999 | 48.0999 | CCLD- | 435 | LabradorRetriever | 178695 | 10y2m | 116.2 | 22.1 | 368.506244 | 2 | 4.0840485 | 3.6 | 3.55418047 | 3.7 | 2.58870554 | 1.3 | 1.02416244 | 1.9 | 3.40518431 | 4.4 | 11.8204936 | 1.3 | 1.59856652 | 4.9 | 25.1824536 | 2.41976736 | -0.927941 | -0.5841457 | -0.6692767 | 0.72842499 | 0.27574424 | -0.5875511 | 0.01950388 | 0.21883635 | 0.12244711 |
| n/a | 1 | 2.4768 | 19.9500 | 19.9500 | CCLD- | na | Mixed | 205236 | 10y4m | 111 | 18.6 | 265.155238 | 1.6 | 2.59635196 | 3.3 | 3.26083482 | 3.3 | 2.38209805 | 1 | 1 | 1.4 | 1.90089628 | 3.5 | 8.07163342 | 1.1 | 1.18579638 | 3.9 | 15.8438657 | -0.9903652 | -1.361686 | 0.00393513 | -0.059958 | -0.394952 | 0.00446839 | -0.4007081 | 0.07714764 | 0.49005039 | -0.0081489 |
| n/a | 1 | n/a | n/a | n/a | CCLD- | na | Mixed | 170259 | 11y7m | 109.8 | 21 | 334.284803 | 1.7 | 2.9363736 | 3 | 2.96722201 | 3.8 | 2.63938458 | 1.2 | 1.01672966 | 1.6 | 2.45281643 | 3.8 | 9.25761798 | 1.1 | 1.18579638 | 4.2 | 18.4160319 | 0.0346438 | -1.9944678 | -0.018223 | 0.10841905 | 0.07942714 | -0.2834106 | -0.3872275 | -0.3061306 | -0.0526763 | -0.0269378 |
| n/a | 1 | n/a | n/a | n/a | CCLD- | na | Mixed | 153529 | 11y9m | 120.9 | 24.8 | 459.206745 | 1.8 | 3.29763957 | 4.1 | 4.04255575 | 3.8 | 2.63938458 | 1.4 | 1.03109256 | 2.2 | 4.50489733 | 5.5 | 17.1468601 | 1.5 | 2.06467249 | 5.7 | 34.2315021 | 4.8680267 | -0.2563997 | -0.904514 | -1.9498128 | 0.75664652 | 0.85860572 | 1.12625239 | -0.1974762 | -0.276907 | -0.2504737 |
| n/a | 1 | 3.0345 | 39.0000 | 39.0000 | CCLD- | na | Mixed | 242721 | 12y0m | 103.7 | 20.5 | 319.255304 | 1.8 | 3.29763957 | 2.8 | 2.77131856 | 3.3 | 2.38209805 | 1.3 | 1.02416244 | 1.8 | 3.0712503 | 4.1 | 10.5077732 | 1 | 1 | 4.3 | 19.3170572 | -0.0287811 | -3.1079612 | 0.94479471 | 0.28596091 | 1.02552626 | 0.49312572 | -0.4119335 | -0.3700134 | -0.1888848 | -0.1653884 |
| n/a | 2 | 2.0977 | 9.9000 | 11.5305 | CCLD- | na | Mixed | 229489 | 12y9m | 122 | 14.3 | 160.522715 | 1 | 1 | 1.7 | 1.69100321 | 2 | 1.65519363 | 0.6 | 0.95457875 | 1 | 1 | 2.5 | 4.60644611 | 0.9 | 0.82829608 | 2.7 | 7.51049292 | -4.6623443 | 1.6788593 | -1.1427517 | -0.6969393 | -0.1960832 | -0.0112603 | -0.1872443 | 0.09031309 | -0.2584533 | -0.0784907 |
| n/a | 1 | n/a | n/a | n/a | CCLD- | na | Mixed | 181147 | 14y2m | 116.5 | 25.2 | 473.449467 | 2.1 | 4.50925887 | 4.6 | 4.53033433 | 4.2 | 2.83858716 | 1 | 1 | 2.2 | 4.50489733 | 5.5 | 17.1468601 | 1.7 | 2.58251392 | 5.8 | 35.4616408 | 5.89031574 | -0.3598044 | 0.7531837 | -2.6064878 | 0.10430849 | 0.3573463 | 0.03776482 | 0.40064575 | 0.29449037 | -0.182805 |
| n/a | 1 | 2.6028 | 23.5000 | 23.5000 | CCLD- | na | Mixed | 218865 | 15y0m | 117.3 | 21 | 334.284803 | 1.5 | 2.27753603 | 2.9 | 2.86928718 | 3 | 2.2226299 | 1.2 | 1.01672966 | 1.5 | 2.16849394 | 3.2 | 6.95160383 | 1.1 | 1.18579638 | 3.5 | 12.7191512 | -1.0958384 | -0.6338218 | -1.4947877 | -0.0370852 | -0.1842821 | 0.09767873 | -0.4235752 | 0.43870464 | -0.4912143 | -0.072081 |
| n/a | 2 | 2.4076 | 15.6000 | 18.1693 | CCLD- | na | Mixed | 222374 | 2y11m | 115.2 | 12.6 | 126.068728 | 1.1 | 1.21346471 | 2.2 | 2.18272214 | 2.4 | 1.88979011 | 0.6 | 0.95457875 | 1.1 | 1.19955078 | 2.7 | 5.2370099 | 0.8 | 0.67100367 | 3.1 | 9.94178206 | -4.3551171 | 0.50440943 | 0.04711124 | -0.05464 | -0.1288167 | -0.0834141 | 0.07654552 | -0.0952641 | 0.35010722 | 0.03981792 |
| n/a | 1 | 1.6669 | 5.4000 | 5.4000 | CCLD- | na | Mixed | 207863 | 2y5m | 111.8 | 11.1 | 98.9741012 | 0.7 | 0.48478483 | 1.1 | 1.09895209 | 2 | 1.65519363 | 0.8 | 0.97989872 | 0.8 | 0.65312873 | 2.2 | 3.72236202 | 0.7 | 0.5284882 | 2.3 | 5.42384796 | -6.031148 | -0.7947351 | -0.5482201 | -0.247087 | 0.06792394 | -0.0367775 | -0.0078512 | -0.3068966 | -0.2828382 | -0.0020002 |
| n/a | 2 | 2.7293 | 23.6000 | 27.4869 | CCLD- | na | Mixed | 211109 | 4y0m | 108.2 | 21.5 | 349.643147 | 1.6 | 2.59635196 | 3.2 | 3.1629948 | 3.6 | 2.53765114 | 1 | 1 | 1.5 | 2.16849394 | 3.9 | 9.66728896 | 1.1 | 1.18579638 | 4.3 | 19.3170572 | -0.2182712 | -1.8496032 | 0.63420193 | -0.0180112 | -0.4439472 | 0.00654195 | -0.1728552 | -0.4582544 | -0.0401444 | -0.0779557 |
| n/a | 1 | 2.8580 | 32.0000 | 32.0000 | CCLD- | na | Mixed | 214498 | 4y7m | 114 | 20.3 | 313.335741 | 1.4 | 1.97988476 | 3 | 2.96722201 | 2.9 | 2.16851956 | 1 | 1 | 1.5 | 2.16849394 | 3.4 | 7.69086795 | 1.1 | 1.18579638 | 3.7 | 14.2380168 | -1.3083609 | -0.801454 | -0.5070519 | -0.2326583 | -0.3476328 | 0.25343603 | -0.0549659 | 0.3311979 | -0.3350765 | -0.1114205 |
| n/a | 1 | 3.3677 | 55.0000 | 55.0000 | CCLD- | na | Mixed | 180101 | 4y8m | 117.4 | 25.8 | 495.201577 | 2.2 | 4.95584873 | 4.6 | 4.53033433 | 4.7 | 3.08045694 | 1.6 | 1.04369816 | 2.1 | 4.12208206 | 5.1 | 15.1188752 | 1.5 | 2.06467249 | 5.5 | 31.8373022 | 5.5756704 | -1.2185432 | -0.7418892 | -0.8886003 | 0.40507032 | 0.13917138 | -0.5208288 | -0.0094425 | 0.29887744 | -0.0201052 |
| n/a | 1 | n/a | n/a | n/a | CCLD- | na | Mixed | 158630 | 5y3m | 120 | 22.8 | 391.108832 | 2.1 | 4.50925887 | 3.6 | 3.55418047 | 4.4 | 2.93623016 | 1.6 | 1.04369816 | 2 | 3.75548699 | 4.6 | 12.7296747 | 1.3 | 1.59856652 | 4.9 | 25.1824536 | 3.43975177 | -0.7812177 | -1.5340846 | -0.3522213 | 1.14864629 | -0.280601 | -0.6240636 | -0.1525832 | 0.22119645 | -0.0974348 |
| n/a | 2 | 2.7188 | 23.3000 | 27.1375 | CCLD- | na | Mixed | 185942 | 5y6m | 114.7 | 19.4 | 287.351522 | 1.3 | 1.70335435 | 2.9 | 2.86928718 | 2.8 | 2.11389732 | 1.1 | 1.00871095 | 1.4 | 1.90089628 | 3.3 | 7.31750011 | 1 | 1 | 3.7 | 14.2380168 | -1.806749 | -0.8691238 | -0.9493082 | 0.0141455 | -0.2622673 | 0.3957593 | 0.13508123 | 0.0432216 | -0.2422904 | 0.01302255 |
| n/a | 2 | 3.1019 | 36.0000 | 41.9292 | CCLD- | na | Mixed | 205990 | 6y2m | 114.4 | 21.4 | 346.545204 | 1.5 | 2.27753603 | 3.6 | 3.55418047 | 4 | 2.73966596 | 1.4 | 1.03109256 | 1.6 | 2.45281643 | 3.8 | 9.25761798 | 1 | 1 | 4.3 | 19.3170572 | 0.26763319 | -1.488419 | -1.1121499 | 0.75137144 | -0.1735212 | -0.1402274 | 0.4594894 | -0.395129 | 0.05244829 | 0.06196537 |
| n/a | 2 | n/a | n/a | n/a | CCLD- | na | Mixed | 170004 | 7y7m | 109.1 | 20 | 304.55537 | 1.5 | 2.27753603 | 2.8 | 2.77131856 | 3.4 | 2.43436207 | 1.2 | 1.01672966 | 1.5 | 2.16849394 | 3.4 | 7.69086795 | 1.1 | 1.18579638 | 3.9 | 15.8438657 | -1.0220023 | -2.1465035 | -0.1824738 | -0.0761762 | -0.1805582 | -0.1756663 | -0.2203197 | 0.02915562 | -0.2146317 | 0.09786936 |
| n/a | 2 | 2.9381 | 30.1000 | 35.0575 | CCLD- | na | Mixed | 218306 | 9y11m | 117 | 19.4 | 287.351522 | 1.5 | 2.27753603 | 2.9 | 2.86928718 | 3.8 | 2.63938458 | 1.2 | 1.01672966 | 1.5 | 2.16849394 | 3.6 | 8.45972545 | 1 | 1 | 3.9 | 15.8438657 | -0.685553 | -0.7502972 | -1.3207145 | 0.32940596 | 0.09715464 | -0.5456471 | 0.08952186 | -0.3780855 | 0.06559993 | -0.1101606 |
| n/a | 1 | 3.4698 | 60.7000 | 60.7000 | CCLD- | na | SaintBernard | 231278 | 6y6m | 118.3 | 28.3 | 590.826961 | 2.1 | 4.50925887 | 5 | 4.92017222 | 5.4 | 3.40761298 | 1.8 | 1.05494495 | 2.6 | 6.19703746 | 5.6 | 17.6697108 | 1.4 | 1.82505908 | 5.8 | 35.4616408 | 7.34422366 | -1.2002371 | -0.5478346 | 0.12283141 | 1.48408351 | 0.09121719 | 1.078951 | 0.13548192 | -0.6407146 | -0.3079097 |
| n/a | 1 | 2.6900 | 26.2000 | 26.2000 | CCLD- | na | ShetlandSheepDog | 226093 | 3y6m | 111.6 | 17.8 | 243.810152 | 1.4 | 1.97988476 | 2.7 | 2.67331495 | 3.1 | 2.27624996 | 1.1 | 1.00871095 | 1.3 | 1.65012886 | 3 | 6.24253709 | 0.8 | 0.67100367 | 3.4 | 11.9922922 | -2.5247026 | -1.4235225 | -0.5977987 | 0.84699217 | -0.0098566 | -0.0188692 | -0.1984301 | -0.2670513 | 0.05796884 | 0.04711241 |
| n/a | 2 | 2.1893 | 11.4000 | 13.2776 | CCLD- | na | ShetlandSheepDog | 218136 | 7y1m | 118.8 | 11.5 | 105.894201 | 0.9 | 0.80744378 | 1.3 | 1.29659373 | 1.9 | 1.59460774 | 0.7 | 0.96806367 | 0.9 | 0.81780347 | 2.2 | 3.72236202 | 0.6 | 0.40117553 | 2.3 | 5.42384796 | -5.7889437 | 0.82834501 | -1.1730538 | 0.05222562 | 0.3383014 | 0.08052609 | -0.1226758 | -0.2891892 | -0.2022747 | -0.0236196 |
| n/a | 2 | 1.9439 | 7.7000 | 8.9682 | CCLD- | na | ShihTzu | 231688 | 10y0m | 113.7 | 7.2 | 43.3159392 | 0.6 | 0.35452514 | 1.1 | 1.09895209 | 1.6 | 1.40732767 | 0.8 | 0.97989872 | 0.8 | 0.65312873 | 1.9 | 2.91529329 | 0.5 | 0.2895732 | 2.1 | 4.50925887 | -6.7725575 | -0.3559008 | -0.8856489 | 0.0554229 | 0.48227041 | 0.39971096 | 0.1090973 | -0.2162255 | -0.0821267 | 0.15088135 |
| PFZ44B11 | 2 | 3.0833 | 35.2951 | 41.1082 | CCLD- | 9282 | SiberianHusky | 218045 | 10y6m | 117 | 23.3 | 407.645313 | 1.6 | 2.59635196 | 3.6 | 3.55418047 | 3.9 | 2.68970078 | 1.2 | 1.01672966 | 1.5 | 2.16849394 | 3.5 | 8.07163342 | 1.1 | 1.18579638 | 3.8 | 15.0300595 | 0.17254461 | -0.7172899 | -1.2793628 | 0.60430067 | -0.7591511 | -0.3486757 | -0.3418183 | 0.08000226 | -0.2907321 | -0.1849723 |
| n/a | 1 | 2.7146 | 27.0000 | 27.0000 | CCLD- | na | StaffordshireBullTerrier | 222325 | 3y8m | 117.6 | 15.6 | 189.528645 | 1.3 | 1.70335435 | 2.6 | 2.57527502 | 3.2 | 2.3293998 | 1 | 1 | 1.5 | 2.16849394 | 3.2 | 6.95160383 | 1 | 1 | 3.5 | 12.7191512 | -2.0376042 | -0.2451783 | -1.1727283 | -0.0993414 | 0.29751415 | -0.4868069 | 0.32118781 | 0.22193977 | 0.20205919 | -0.0404732 |
| n/a | 2 | 3.1019 | 36.0000 | 41.9292 | CCLD- | na | StaffordshireBullTerrier | 217711 | 7y5m | 120.6 | 17.5 | 236.025885 | 1.5 | 2.27753603 | 3.4 | 3.3586452 | 3.5 | 2.48620805 | 0.6 | 0.95457875 | 1.6 | 2.45281643 | 3.4 | 7.69086795 | 0.9 | 0.82829608 | 3.8 | 15.0300595 | -1.2923374 | 1.52329632 | -0.1008332 | 0.64109841 | 0.04415571 | -0.5499193 | 0.26210906 | 0.09432201 | 0.50534107 | -0.1053791 |
| 5807 | 1 | 2.9616 | 30.8999 | 35.9892 | CCLD+ | 5807 | LabradorRetriever | 203201 | 10y1m | 114.1 | 19.5 | 290.185744 | 1.62 | 2.66265856 | 3.3 | 3.26083482 | 3 | 2.2226299 | 0.7 | 0.96806367 | 1.6 | 2.45281643 | 3.6 | 8.45972545 | 1.1 | 1.18579638 | 4.1 | 17.5368355 | -0.7973439 | 0.06430077 | 0.61295497 | -0.2378706 | -0.1412789 | 0.19595188 | -0.2627947 | 0.2548856 | 0.16506027 | 0.02159694 |
| 5546 | 2 | 2.9767 | 36.5999 | 36.5999 | CCLD+ | 5546 | LabradorRetriever | 145239 | 7y1m | 121 | 23.2 | 404.311936 | 1.7 | 2.9363736 | 3 | 2.96722201 | 3.4 | 2.43436207 | 0.7 | 0.96806367 | 1.8 | 3.0712503 | 4.1 | 10.5077732 | 1.3 | 1.59856652 | 4.6 | 22.1512581 | 1.05429398 | 1.21685413 | -0.095189 | -1.1564553 | -0.0222106 | -0.2107872 | -0.1974364 | 0.06264165 | -0.6406504 | -0.0474613 |
| 1908 | 2 | 3.0404 | 33.6999 | 39.2503 | CCLD+ | 1908 | LabradorRetriever | 186286 | 5y5m | 114.6 | 17.4 | 233.457868 | 1.4 | 1.97988476 | 2.85 | 2.82030717 | 2.65 | 2.03095243 | 0.6 | 0.95457875 | 1.45 | 2.0325981 | 3.35 | 7.50325478 | 0.85 | 0.74782682 | 3.75 | 14.6313188 | -2.4623502 | 0.52458462 | 0.64599036 | 0.24773928 | 0.03993052 | 0.29854546 | 0.06115298 | -0.1523804 | 0.12257271 | -0.0590803 |
| na | 2 | 2.7305 | 23.6330 | 27.5253 | CCLD- | 3630 | LabradorRetriever | 195584 | 8y10m | 113.7 | 19.7 | 295.893909 | 1.5 | 2.27753603 | 3.1 | 3.0651242 | 4.1 | 2.78929125 | 1.1 | 1.00871095 | 1.5 | 2.16849394 | 4 | 10.0840271 | 1.25 | 1.49030481 | 4.6 | 22.1512581 | 0.38967211 | -1.248438 | -0.4299207 | -0.83554 | -0.4497147 | -0.7004412 | 0.33171082 | -0.4770486 | 0.32209901 | 0.15584074 |


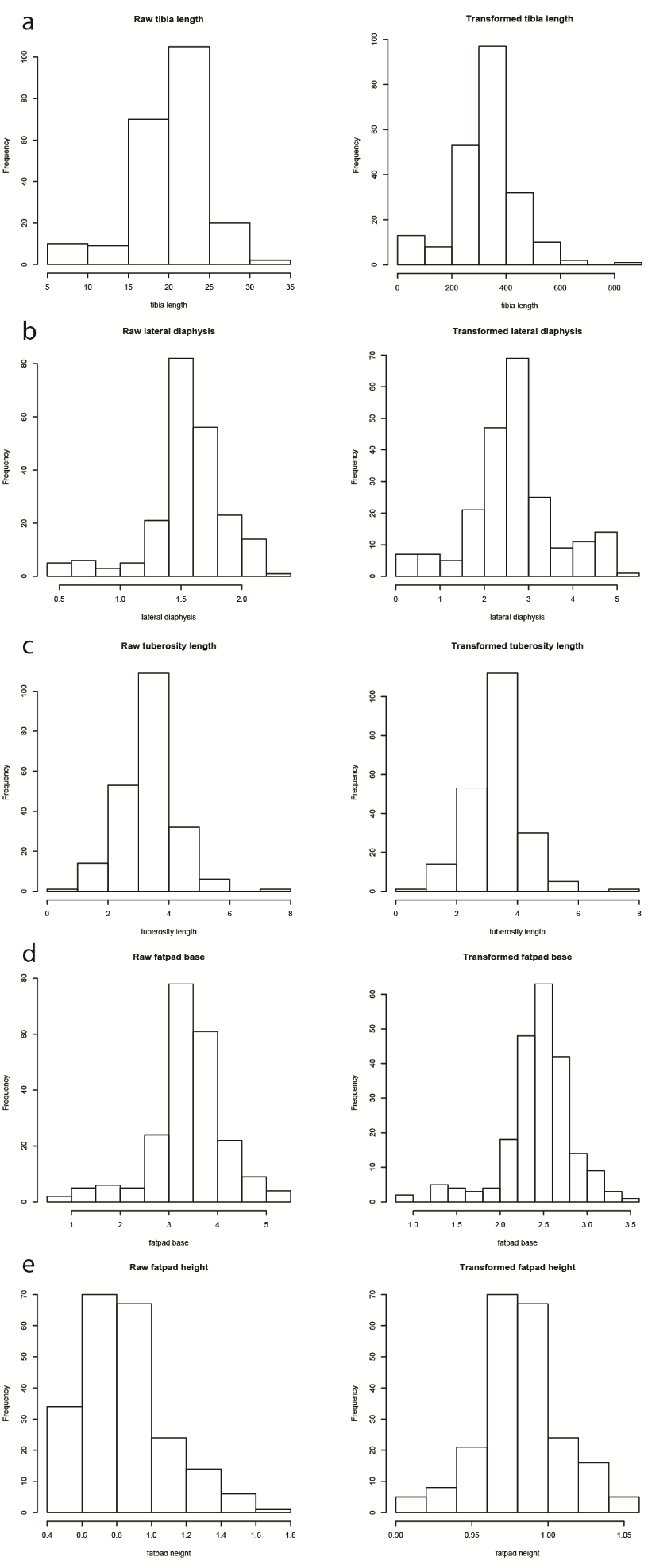


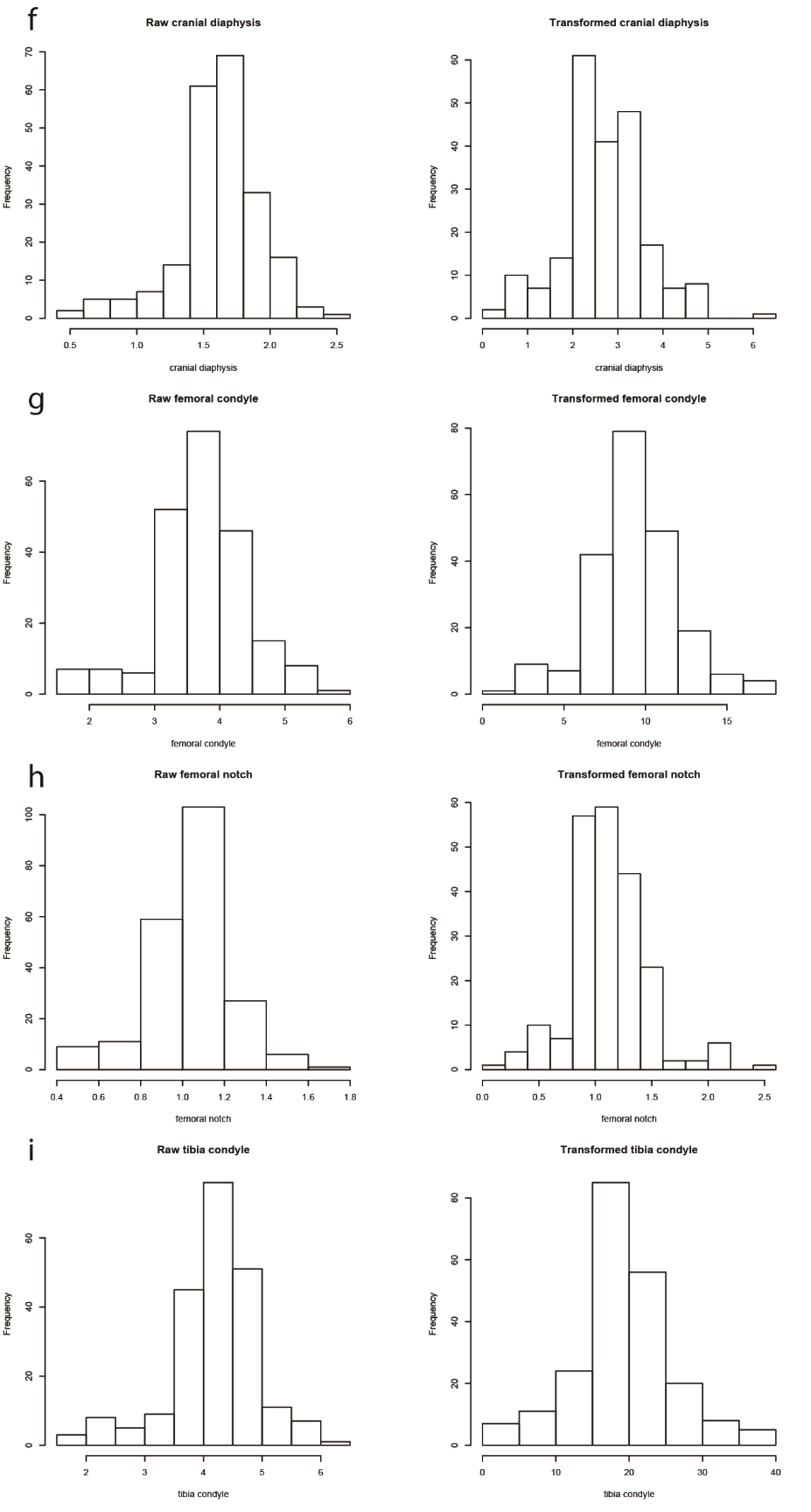


Figure A in S1 file.

Figure B in S1 file.

Figure C in S1 file.

Figure D in S1 file.

Figure E in S1 file.

Figure F in S1 file.

Figure G in S1 file.


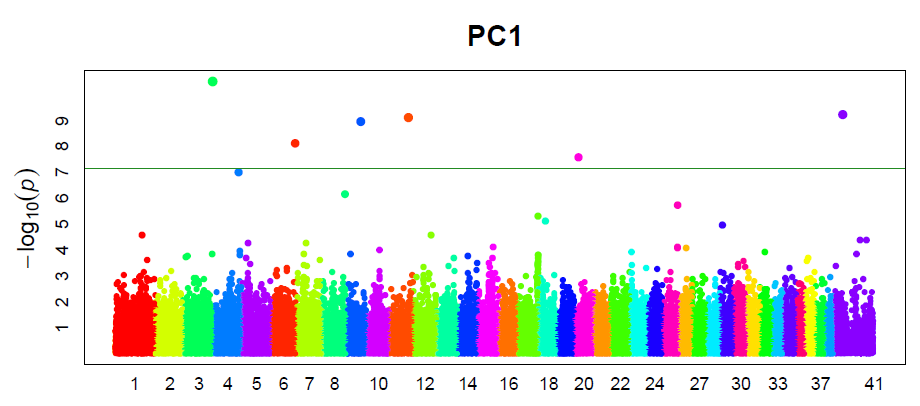


Figure H in S1 file.


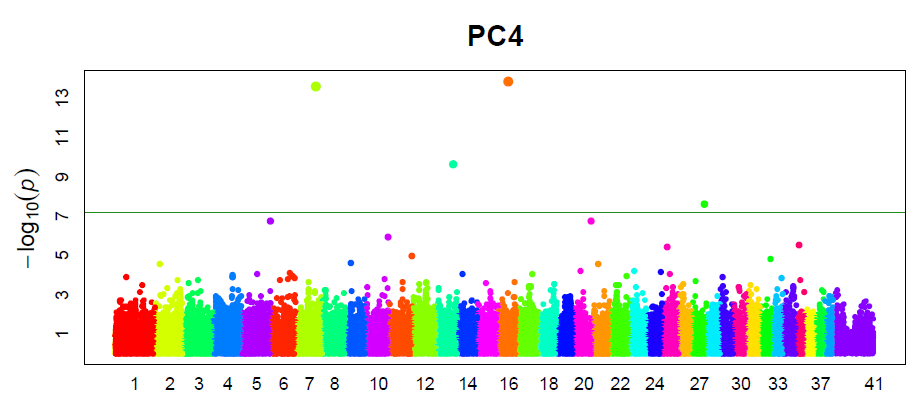


Figure I in S1 file.

Figure J in S1 file.


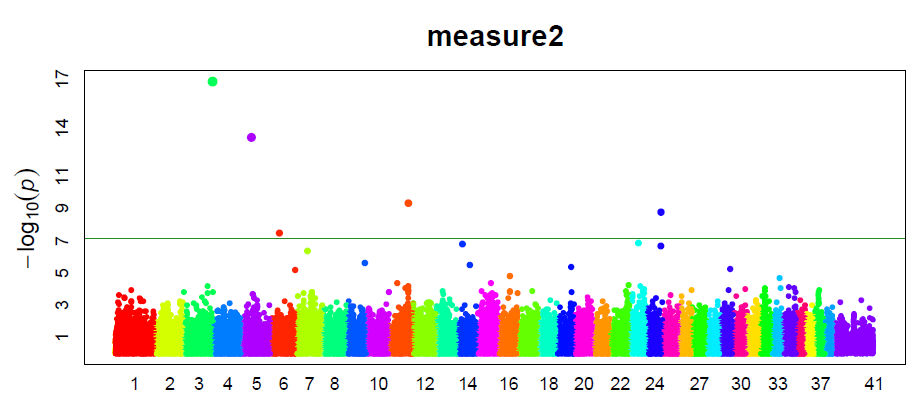


Figure K in S1 file.

Figure L in S1 file.

Figure M in S1 file.

Figure N in S1 file.

Figure O in S1 file.

Figure P in S1 file.

Figure Q in S1 file.

Figure R in S1 file.
